# Community assembly and diversification in Indo-Pacific coral reef fishes

**DOI:** 10.1002/ece3.19

**Published:** 2011-11

**Authors:** Nicolas Hubert, Emmanuel Paradis, Henrich Bruggemann, Serge Planes

**Affiliations:** 1 Laboratoire ECOMAR, Faculté des Sciences et Technologies, Université de La Réunion15 Avenue René Cassin, BP 7151, 97715 Saint-Denis Cedex 9, Réunion, France; 2 Institut de Recherche pour le DéveloppementUR226—ISE-M, 361 rue Jean-François Breton, BP 5095, 34196 Montpellier cedex 5, France; 3 USR 3278 CNRS-EPHE, CRIOBE–CBETM, Université de Perpignan Via Domitia52 Avenue Paul Alduy, 66860 Perpignan cedex, France

**Keywords:** Chaetodontidae, coral reef fishes, Labridae, lineage through time plot, Phylogenetic community structure, Pomacentridae, sea levels, theories of coexistence

## Abstract

Theories of species coexistence have played a central role in ecology and evolutionary studies of the origin and maintenance of biodiversity in highly diverse communities. The concept of niche and associated theories predict that competition for available ecological space leads to a ceiling in species richness that influences further diversification patterns. By contrast, the neutral theory supports that speciation is stochastic and diversity independent. We examined the phylogenetic community structure and diversification rates in three families and 14 sites within coral reef fish communities from the Indian and Pacific oceans. Using the phylogenetic relationships among 157 species estimated with 2300 bp of mitochondrial DNA, we tested predictions in terms of species coexistence from the neutral and niche theories. At the regional scale, our findings suggest that phylogenetic community structure shifts during community assembly to a pattern of dispersion as a consequence of allopatric speciation in recent times but overall, variations in diversification rates did not relate with sea level changes. At the local scale, the phylogenetic community structure is consistent with a neutral model of community assembly since no departure from a random sorting of species was observed. The present results support a neutral model of community assembly as a consequence of the stochastic and unpredictable nature of coral reefs favoring generalist and sedentary species competing for living space rather than trophic resources. As a consequence, the observed decrease in diversification rates may be seen as the result of a limited supply of living space as expected in a finite island model.

## Introduction

The heterogeneous distribution of biodiversity on Earth has called considerable attention in ecology and evolutionary biology (MacArthur and Wilson 1967; Nelson and Platnick 1981). Because ecosystems are dynamic and species permanently adapt to changing landscapes, understanding the evolutionary mechanisms structuring communities is a challenge (Ricklefs and Schluter 1993; Leibold et al. 2004). Species that live together in a local community do so because they are present in the larger regional species pool and have characteristics that permit their existence at a given locality and their coexistence with other species in the community (Webb et al. 2006). In this context, species richness results from the interaction of biotic and abiotic factors determined at both local and regional scales (Ricklefs 1987; Ricklefs and Schluter 1993; Cornell 1993; Holt 1993; Caley and Schluter 1997; Losos and Schluter 2000; Whittaker et al. 2001; Ricklefs 2004).

According to the niche theory of species coexistence (Hutchinson 1959; Cornell and Lawton 1992; Leibold 1998), species occupy parts of the ecological space available and coexist locally if they are able to partition it in a way that minimizes ecological overlap otherwise they mutually exclude (limiting similarity hypothesis, Hutchinson 1959; Saturation hypothesis, Terborgh and Faaborg 1980; regional similarity hypothesis, Mouquet and Loreau 2002). In this context, one may expect that factors influencing the breadth of the ecological space available locally will influence species richness (species-energy hypothesis and primary production, Currie 1991; habitat heterogeneity, Fraser 1998; Guéguan et al. 1998).

Until recently, community assembly was seen as the result of species immigration from elsewhere in the regional pool followed by habitat filtering and biotic interactions at the local scale (Loreau and Mouquet 1999; Mouquet and Loreau 2003; Leibold et al. 2004; Holt 2009). By contrast, the ecological filling of niches through diversification has been traditionally considered to act on much larger scales than those involved in community assembly and to concern the evolution of the regional pools of species (Qian and Ricklefs 2000; Chase 2003; Wiens and Donoghue 2004; Weir and Schluter 2007). Worth noting, the scarcity of documented cases of community assembly through adaptive radiation supported the hypothesis that speciation contributes to community assembly only in particular ecosystems (Losos et al. 1998; Ricklefs and Bermingham 2001; Gillespie 2004; Duponchelle et al. 2008). The neutral theory of biodiversity challenged this view by proposing a model that explicitly assumes both an equivalent per capita fitness of species and a significant contribution of speciation in increasing species richness at the local scale (ecological drift model, Hubbell 2001). According to the ecological drift model, immigration and speciation counterbalance species loss by extinction at local scales and niche partitioning has no stabilizing effect on species richness (Mouquet and Loreau 2002; Kraft et al. 2008; Levine and HilleRisLambers 2009).

Clades diversify in an ecological context, yet most macroevolutionary models do not directly relate the dynamic of community assembly to large-scale evolutionary patterns (Kraft et al. 2007; McPeek 2008; Ackerly 2010; Ricklefs 2010). The neutral and niche theories of coexistence, however, are likely to affect patterns of diversification differently (Table 1). In a niche context, a lineage may initially diversify in a relatively empty ecological space and speciation rate should slow down as more niches become occupied (Schluter 2000). By contrast, the neutral model predicts no such decrease in net diversification since speciation and extinction, as stochastic and diversity independent processes, will be constant (Hubbell 2001; Ricklefs 2003). Yet, studies that explored the dynamic of community assembly and its impact on diversification gathered results either in silico (Chave et al. 2002; Mouquet and Loreau 2003; Allouche and Kadmon 2009; Chisholm and Lichstein 2009; Vergnon et al. 2009) or from communities of sessile organisms with limited dispersal abilities (Webb 2000; Cavender–Bares et al. 2004; Ackerly et al. 2006; Hardy and Senterre 2007; Kraft et al. 2007; Levine and HilleRisLambers 2009; review in Vamosi et al. 2009).

**Table 1:** Summary of the predictions tested in the present study

	Theory	Assumptions	Predictions	References
Community assembly	Niche	(1) Species have ecological preferences and differential fitness	Phylogenetic relatedness depart from expected under random species sorting	Hutchinson (1959); Pianka (1976); Anderson et al. (1981); Webb (2000); Webb et al. (2002); Hardy and Senterre (2007); Holt (2009)
		(2) Habitat filtering and/or competition drive community assembly		
	Neutral	(1) Species have equivalent individual fitness	Phylogenetic relatedness does not depart from expected under random species sorting	Sale (1977, 1978); Hubbell (2001); Webb (2000); Webb et al. (2002); Hardy and Senterre (2007)
		(2) Community assembly is a random process		
Diversification	Niche	(1) The ecological space is limited in nature	Diversification rates are diversity dependent	Ricklefs (1987); Schluter (2000); Rüber and Zardoya (2005); McPeek (2008); Phillimore and Price (2008); Rabosky (2009); Pigot et al. (2010); Ricklefs (2010)
		(2) Species richness is ceiled by competition		
	Neutral	(1) The ecological space does not limit species coexistence	Diversification rates are diversity independent	Hubbell (2001); McPeek (2008)
		(2) Species accumulation is a stochastic process		

Coral reefs are among the most diverse and structurally complex ecosystems (Sale 1991). Currently exhibiting more than 5000 species (Froese and Pauly 2011), that is, 15% of the world's ichthyofauna, that coexist and aggregate in some of the largest vertebrate communities, the Indo-Pacific coral reef fish remarkably reflects this complexity (Bellwood and Hughes 2001; Mora et al. 2003; Froese and Pauly 2011). Community assembly in coral reef fish has been subject of intensive debate for decades (Sale 1977, 1978; Anderson et al. 1981; Fraser and Currie 1996; Sale 1998; Almany 2004). Since coral reef fish tends to be generalists forming heterospecific feeding schools, several authors opposed to the conventional niche theory, a stationary model of coexistence, which emphasizes the role of stochastic recruitment and mortality in determining local coexistence of species (lottery model, Sale 1977, 1978; Chesson and Warner 1981; Warner and Chesson 1985). Although not explicitly assuming equivalent individual fitness among species, the lottery model makes predictions about community assembly that are very similar to the neutral theory since ecological overlap has no influence on coexistence (Table 1). By contrast, large-scale patterns of species richness in coral reefs are reliably predicted by ecological factors including primary productivity and disturbance (Fraser and Currie 1996), a pattern generally interpreted as a consequence of a niche-based model of community assembly (Hutchinson 1959; Terborgh and Faaborg 1980; Mouquet and Loreau 2002).

In this context, we focused on several predictions from the neutral and niche theories related with community assembly and diversification pattern in coral reef fish from the Indian and Pacific oceans (Table 1). We inferred phylogenetic relationships among 157 species from the families Chaetodontidae, Pomacentridae, and Labridae, being among the most diverse tropical reef fish lineages (Bellwood and Hughes 2001; Mora et al. 2003), to assess the phylogenetic structure of communities and pattern of diversification underlying those communities. The results are discussed in light of the predictions drawn from the neutral versus niche-based theory to unravel mechanisms underlying community assembly for coral reef fish.

## Materials and Methods

### Sampling design, specimens storage, and species identification

We focused our study on the phylogenetic diversity of a limited set of abundant families in order to avoid potential bias due to the inclusion of rare taxa with long branches that can lead to spurious phylogenetic structure (Kembel and Hubbell 2006; Hardy and Senterre 2007; Vamosi et al. 2009). We selected the families Pomacentridae, Chaetodontidae, and Labridae that are among the most speciose and whose taxonomy is regularly updated (Froese and Pauly 2011). Several phylogenies have been published during the last decade for these families (Westneat and Alfaro 2005; Fessler and Wesneat 2007; Cooper et al. 2009). Nevertheless, these phylogenies were inferred from different sets of molecular markers and samplings were focussed on taxonomic rather than geographical coverage. In this context, we gathered new phylogenetic data based on the same set of molecular markers for all families and covering all the species in the sampled communities in order to limit bias in our phylogenetic inferences.

**Figure 1:**
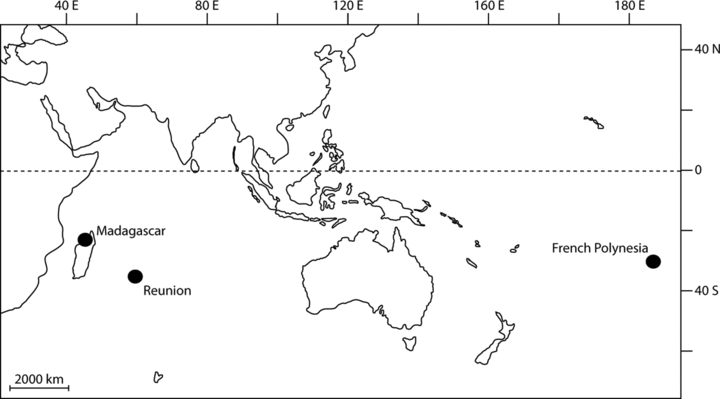
Location of the sampled communities.

The sampling was conducted in sites across both inner reefs and outer slopes within Madagascar, Réunion, and French Polynesia (Fig. 1; Appendixes 1 and 2). At each site, specimens were caught by rotenone poisoning performed in sampling plot measuring 20 × 20 m^2^. Poisoning was conducted with a constant amount of rotenone and duration. All species were collected; fresh specimens were labeled and photographed. Typically, a sample of white muscle was fixed in 90% ethanol and fresh specimens were conserved in a 10% formaldehyde solution. Identifications were done independently by three of us and further confirmed through examination of morphological characters (color, meristic counts). Recent molecular studies regularly emphasized the presence of cryptic diversity in coral reef fish including in the three selected families (McCafferty et al. 2002; Kuriiwa et al. 2007; Drew and Barber 2009; Steinke et al. 2009; Leray et al. 2010). In this context, several specimens from different sites and islands were sampled for each species and the three mitochondrial markers were sequenced in order to consider biological units and preclude taxonomic bias in the community analyses. The presence of the 157 sampled species was recorded in each of the 14 geographical and ecological sampling units and habitat characteristics such as depth and location on the inner reef or outer slope were registered (Appendixe 1). Worth noting, specimens in French Polynesia were collected during the Moorea biocode project between 2006 and 2008 across more than 30 sites (http://mooreabiocode.org/). This project was designed to collect all the species from the island but not all the species for each site were recorded as in Reunion and Madagascar. Since habitat type and depth were recorded for each specimen, however, we listed the species from each kind of habitat (inner reef and outer slope) and treated each as a single site in our analyses.

### DNA extraction, PCR primers, and sequencing

Genomic DNA was extracted using the QIAGEN DNeasy 96 Blood and Tissue Kit (QIAGEN Sciences, Germantown, USA) according to manufacturer specifications and further used with no dilution for amplification and sequencing. Three fragments of the mitochondrial genome were targeted for phylogenetic analyses and given the community-based approach developed here, several arguments justify this choice: (1) previous phylogenetic studies of the families Pomacentridae, Labridae, and Chaetodontidae did not detect significant discrepancies between mitochondrial and nuclear sequences (Westneat and Alfaro 2005; Fessler and Westneat 2007; Cooper et al. 2009), (2) mitochondrial genes generally exhibit higher substitution rates than nuclear genes (e.g., Moore 1995; Moriyama and Powell 1997; Saccone et al. 1999), (3) ease of sequencing because of large libraries of primers available for the amplification of homologous sequences for a vast array of taxa (e.g., Ivanova et al. 2007). In this context, a 650-bp segment from the 5′ region of the cytochrome oxydase I gene (COI) was first amplified using the primers FF2d-5′-TTCTCCACCAACCACAARGAYATYGG-3′ and FR1d-5′-CACCTCAGGGTGTCCGAARAAYCARAA-3′ (Ivanova et al. 2007). Second, another 550-bp segment from the large ribosomal subunit 16S was amplified using the primers 16SarL-5′-ACGCCTGTTTATCAAAAACAT-3′ and 16SbrH-5-′CCGGTCTGAACTCAGATCACGT-3′ (Palumbi et al. 1991). Finally, 1071-bp segment corresponding to the entire cytochrome b (Cytb) was amplified using either the primers CytbL14724-5′-TGACTTGAARAACCAYCGTTG-3′ (Palumbi et al. 1991) and CytbH15915-5′-AACTGCCAGTCATCTCCGGTTTACAAGAC-3′ (Irwing et al. 1991) or Glufish-F-5′-AACCACCGTTGTTATTCAACTACAA-3′ and TruccytB-R-5′-CCGACTTCCGGATTACAAGACCG-3′ (Sevilla et al. 2007). When amplifications of the entire Cytb failed with these two sets of primers, a shorter 550-bp fragment was amplified using the primers CytTHAL-5′-AACGGAGCATCNTTCTTCTTT-3′ (Bernardi et al. 2004) and CB3A-5′-GGCAAATAGGAARTATCATTC-3′ (Kocher et al. 1989).

Polymerase chain reaction (PCR) amplifications were performed in 27 µl of solution including 10.7 µl of ultrapure water, 2.5 µl of 10× PCR buffer, 1.5 µl of MgCl_2_ (25 mM), 2.5 µl of each primers (10 mM), 3 µl of each dNTP (2 mM), 0.3 µl of *Taq* DNA polymerase (5 U/µl), and 4 µl of template DNA. The PCR conditions consisted of 94°C (5 min), 10 cycles of 94°C (1 min), 64–54°C or 60–50°C decreasing 1°C per cycle (1 min), 72°C (1 min 30 sec) followed by 25 cycles of 94°C (1 min), 54°C or 50°C (1 min), 72°C (1 min 30 sec), with a final extension at 72°C (5 min).

All sequences generated for this publication have been deposited in GenBank (Appendixe 1) and BOLD (project IPCOM). GenBank accession numbers for the outgroup sequences obtained from published mitochondrial genomes are also provided Appendix 2.

### Phylogenetic reconstructions and divergence time estimates

Protein-coding regions were aligned manually while 16S sequences were first aligned through multiple alignments using Clustal W (Thompson et al. 1994) and manually refined. Aligned datasets ranged between 2319 bp for the Chaetodontidae and 2334 bp for the Pomacentridae and reached 2333 bp in the Labridae. Phylogenetic relationships within each family were assessed using the BIONJ algorithm (Gascuel 1997) as implemented in PAUP*4.0b10 (Swofford 2002) and statistical support assessed with bootstrap proportion (BP) (Felsenstein 1985) through 2000 replicates. Once a tree was computed for each family, a final cladogram based on the topologies inferred for each family was build and a new alignment including a single, randomly picked individual for each 157 species was used for branch length optimization of the final cladogram in maximum likelihood (ML) using PhyML (Guindon and Gascuel 2003). ML optimizations were conducted under the GTR+I+Γ model with parameters optimized simultaneously during branch length computations. All trees generated in the present study have been submitted to Treebase and are available at http://purl.org/phylo/treebase/phylows/study/TB2:S11274.

The heterogeneity in substitution rate across lineages was first assessed through the likelihood procedure implemented in r8s (Sanderson 2003). Divergence times were reconstructed under the assumption of a molecular clock following the likelihood approach implemented in the Langley–Fitch method (LF method, Langley and Finch 1974) and then by relaxing the assumption of constant rate across the tree in using *k* separate rate parameters. Likelihood scores computed for the clock-like and the nonclock models were then compared with a likelihood ratio test (LRT). Since a model with two rate parameters (*k* = 2; nonclock) was compared with a single rate parameter (*k* = 1; clock-like) model, the LRT had one degree of freedom.

The heterogeneity in absolute substitution rate among lineages was further explored through the penalized likelihood model developed by Sanderson (2002) and also implemented in r8s. This approach combines a parametric model having different substitution rates on each branch with a nonparametric roughness penalty that limit the heterogeneity of the substitution rates across the tree. Thus, it assumes that substitution rates tend to be correlated on contiguous branches and the optimality criterion becomes the log likelihood of the parametric model minus the roughness penalty (Sanderson 2002). The relative contribution of each component is determined by a smoothing parameter (*S*) whose optimal values are determined through a cross-validation procedure that measures the fit between observed and predicted numbers of substitution in terminal branches according to the model. The *S* value maximizing the performance of the predictions is determined by a cross-validation score (*CV*) that corresponds to the squared deviations between observed and inferred values and standardized by the observed ones. This method requires several reference ages across the phylogeny to produce robust inferences (Britton et al. 2007), therefore several age estimates based on published molecular phylogenies of Chaetodontidae, Pomacentridae, and Labridae were used as calibration points (Table 2).

**Table 2:** Clade ages reported in previous molecular phylogenetic studies of Chaetodontidae, Pomacentridae, and Labridae

Clade age	Millions years ago (Ma)	Source
Chaetodontidae	37	Fessler and Westneat (2007)
Chaetodontidae, Bannerfishes^1^	24	Fessler and Westneat (2007)
Chaetodontidae, *Chaetodon*, clade 2	14	Fessler and Westneat (2007)
Chaetodontidae, *Chaetodon*, clade 3	14.5	Fessler and Westneat (2007)
Chaetodontidae, *Chaetodon*, clade 4	14	Fessler and Westneat (2007)
Pomacentridae, *Dascyllus*	16	McCafferty et al. (2002)
Pomacentridae, *D. flavicaudus*	11.8	McCafferty et al. (2002)
Pomacentridae, *D. carneus* and *D. trimaculatus*	3.9	McCafferty et al. (2002)
Labridae, Cheilinae^2^	13	Smith et al. (2008)
Labridae, *Thalassoma*^3^	10	Bernardi et al. (2004)
Labridae, *Macropharyngodon bipartitus*	11.3	Read et al. (2006)
Labridae, *M. meleagris*	11.3	Read et al. (2006)

1 *Heniochus, Forcipiger*, and *Hemitaurichthys*.

2 *Pseudodax, Cirrhilabrus, Pseudocheilinus, Oxycheilinus, Wetmorella, Epibulus*, and *Cheilinus*.

3 Except *T. ballieui* and *T. septemfasciata*.

### Diversification rates and phylogenetic community structure

We first checked for potential departures from a stationary model of diversification through a lineage through time (LTT) plot analyses (Harvey et al. 1994) using APE 2.6-3 (Paradis et al. 2004) and based on the chronogram with the optimal smoothing parameter. The LTT plot corresponds to a lineage accumulation curve showing time versus number of lineages that we used to check the plausibility of models of macroevolution by comparing observed and expected LTT plot under a given speciation model (Nee et al. 1994). The goodness-of-fit of several alternative time-dependent birth–death models of diversification (Nee 2006) was determined through the Akaike Information Criterion (AIC) as implemented in APE. We first fitted to each family a birth–death model (Nee et al. 1994) in order to estimate speciation and extinction rates separately. In all three cases, the estimated extinction rate was equal to zero. Giving this result and the notorious difficulties to estimate extinctions with molecular phylogenies (Kubo and Iwasa 1995; Stadler 2009; Paradis 2011), we focused on a Yule model considering speciation as a proxy for diversification. We also considered a time-dependent extension of the Yule model as implemented in APE that allows the user to specify any arbitrary model.

We analyzed the phylogenetic structure of communities through an additive partitioning of phylogenetic diversity within and between sites following the model proposed by Hardy and Senterre (2007) and implemented in Spacodi (Hardy 2007). Providing that a “community” is defined as any assemblage of species spatially localized (e.g., all the fish species in a transect of 100 m in a reef or 20 × 20 m^2^ plots as here) and picked from a regional pool (e.g., an archipelago or an ocean), partitioning the species phylogenetic distances among communities from a regional pool may document the processes ruling community assembly and species coexistence (Webb 2000; Webb et al. 2002; Hardy and Senterre 2007). This hypothesis relies on the assumption that phylogenetic distance mirrors ecological divergence (i.e., niche conservatism), a prerequisite supported in marine fish by recent phylogenetic studies (Streelman et al. 2002; Rüber et al. 2003; Rüber and Zardoya 2005; Fessler and Westneat 2007). According to Hardy and Senterre (2007), the measure of the mean phylogenetic distance between distinct species (Δ^P^) can be evaluated at different levels with Δ^P^_S_, the average within sites, and Δ^P^_T_, the average among all sites, and both estimates can be used to estimate the gain of phylogenetic distance between species occurring in different sites compared with species occurring in the same site with Π_ST_ = (Δ^P^_T_–Δ^P^_S_)/Δ^P^_T_. Then, Π_ST_ can be computed after randomizing the species in the tree in order to test for significant departure from a model of random association of species according to their phylogenetic relatedness. If partial randomizations are performed according to threshold of phylogenetic divergence (e.g., less than 2% or 5 Millions years [Myr]), changes in community phylogenetic structure through time may be detected.

The Π_ST_ index was computed at two spatial scales, among islands (i.e., regional scale influenced by geographic isolation among islands and oceans) or between inner reef and outer slope within island (i.e., local scale influenced by individual interactions, competition, and habitat choice), through two distinct analyses. In the first analysis, we assessed the phylogenetic community structure among islands by calculating Π_ST_ using the entire species pools (i.e., all the plots) from Madagascar, Reunion, and French Polynesia (regional scale). This analysis aimed at estimating the influence of large-scale geographic isolation on community assembly likely influenced by historical factors. In the second analysis, we estimated the phylogenetic community structure between contiguous habitats within islands by calculating Π_ST_ using species list from the inner reef and outer slope following occurrence of species in the sampled 20 × 20 m^2^ plots (local scale). This second analysis aimed at detecting departures from a random model of community assembly as a consequence of biotic interactions (e.g., Π_ST_ < 0 as a consequence of competitive exclusion) or ecological constraints (e.g., Π_ST_ > 0 as a consequence of habitat filtering). In both analyses, the Π_ST_ index was plotted against time and computed every 5 Myr at the regional scale and each 10 Myr at the local scale.

## Results

**Figure 2:**
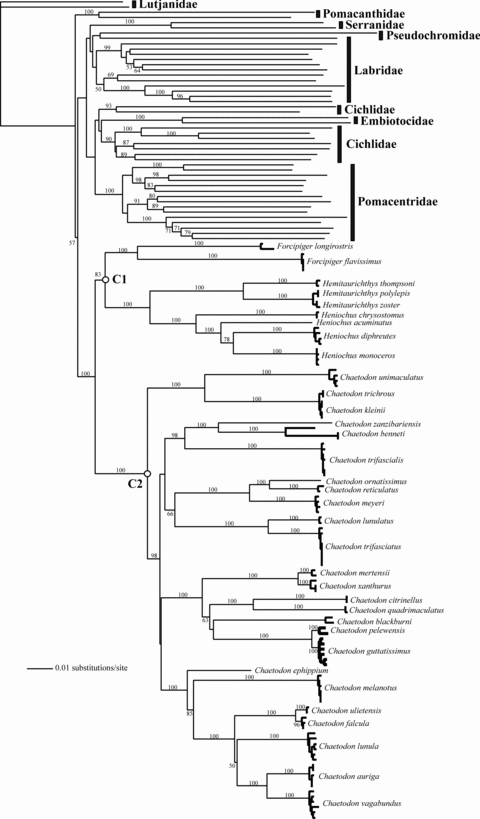
Phylogenetic relationships within Chaetodontidae among 109 specimens belonging to 33 species and four genera with 46 outgroup taxa. This BIONJ tree is based on the 16S+COI+Cytb dataset including 593 bp with 276 variable and 236 informative sites in 16S, 655 bp with 308 variable and 276 informative sites in COI, 1071 bp with 607 variable and 554 informative sites in Cytb. The bootstrap percentages are derived from 2000 replicates and labeled at nodes. The tree presented here is very similar to the phylogeny published by Fessler and Westneat (2007) excepting the position of *C. unimaculatus, C. trichrous, C. kleinii* within the genus *Chaetodon*.

**Figure 3:**
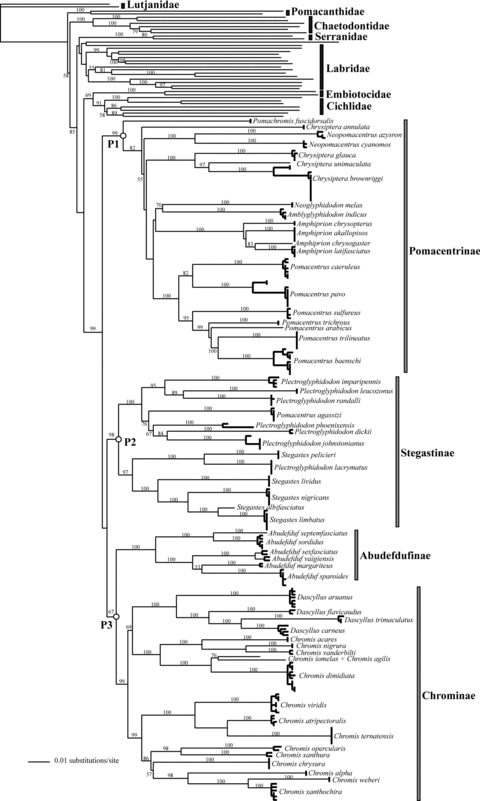
Phylogenetic relationships within Pomacentridae among 220 specimens belonging to 58 species and 12 genera with 37 outgroup taxa. This BIONJ tree is based on the 16S+COI+Cytb dataset including 608 bp with 293 variable and 264 informative sites in 16S, 655 bp with 311 variable and 285 informative sites in COI, 1071 bp with 595 variable and 541 informative sites in Cytb. The bootstrap percentages are derived from 2000 replicates and labeled at nodes. The tree presented here is divided in four highly supported clades. *Chromis* is paraphyletic with respect to *Dascyllus*. *Stegastes* and *Plectroglyphidodon* are closely related but are not reciprocally monophyletic. The topology supported here (Pomacentrinae [Stegastinae {Abudefdufinae + Chrominae}]) only differed from Cooper et al. (2009) who placed the Pomacentrinae as follows: (Stegastinae [Abudefdufinae {Chrominae + Pomacentrinae}]).

**Figure 4:**
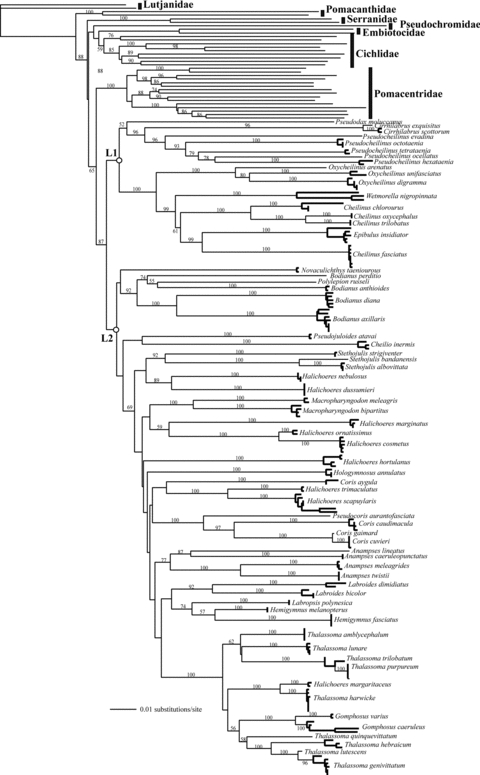
Phylogenetic relationships within Labridae among 184 specimens belonging to 65 species and 24 genera with 33 outgroup taxa. This BIONJ tree is based on the 16S+COI+Cytb dataset including 607 bp with 297 variable and 268 informative sites in 16S, 655 bp with 314 variable and 281 informative sites in COI, 1071 bp with 598 variable and 547 informative sites in Cytb. The bootstrap percentages are derived from 2000 replicates and labeled at nodes. Deep nodes were poorly supported with basal branches coalescing quickly, a pattern previously reported for both mitochondrial and nuclear data (Wesneat and Alfaro 2005). The genera *Cheilinus, Epibulus, Wetmorella, Oxycheilinus*, and *Pseudocheilinus* are closely related (clade L1) and the clade including *Pseudojuloides* and *Cheilio* sort in basal position of the larger clade L2 including *Stethojulis, Halichoeres, Coris, Macropharyngodon, Hologymnosus, Pseudocoris, Labroides, Labropsis, Hemigymnus, Thalassoma*, and *Gomphosus* in agreement with the phylogeny from Westneat and Alfaro (2005).

The 2300 bp from the two mitochondrial genes and ribosomal subunit provided well-supported phylogenies (Figs. 2, 3, 4). The topologies obtained for the three families were grouped into a single cladogram and a single individual for each of the 157 species was randomly picked from each of the three datasets. This composite cladogram including the 157 species and its mirrored dataset was further used for ML branch-length optimization using the GTR+I+Γ model (General Time-Reversible model; log*L* = –78462.14; base frequencies A = 0.249, C = 0.291, G = 0.186, T = 0.274; GTR relative rate parameters A–C = 1.83, A–G = 10.00, A–T = 2.11, C–G = 0.74, C–T = 13.35, G–T = 1.00; proportion of invariant site I = 0.474; gamma distribution shape parameter γ = 0.758).

**Figure 5:**
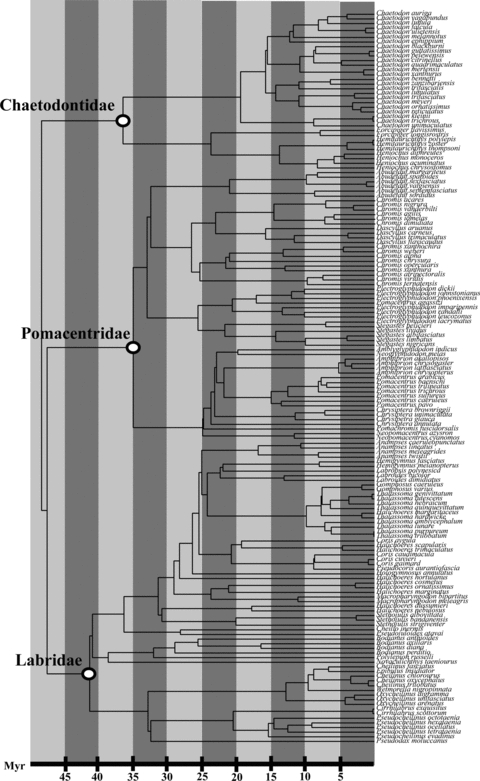
Chronogram of the Chaetodontidae, Pomacentridae, and Labridae families following the clade ages reported in Table 2 and a smoothing parameter *S* = 1000 for the penalized likelihood function (Sanderson 2002).

Together with previous estimates of clade ages (Table 2), the 157 species phylogram obtained from the ML analysis was used to explore the heterogeneity of rates of substitution. The LRT was significant for all the lineages examined except the Chaetodontidae (Table 3). In this context, we used a relaxed molecular clock model estimated through penalized likelihood analyses. The cross-validation procedure indicated that the *CV* scores were lower and best for intermediate values of the smoothing parameter (*S* = 1, *CV* = 3793; *S* = 3.2, *CV* = 3694; *S* = 10, *CV* = 3525; *S* = 32, *CV* = 3325; *S* = 100, *CV* = 3166; *S* = 1000, *CV* = 3040), while a large number of failures (more than 30) were observed in iterations involving smoothing parameter value of *S* = 10,000 and above. This result is consistent with previous observations that intermediate values of *S* between 100 and 1000 often provide the best fit (Sanderson 2002). Thus, we used the chronogram estimated with a smoothing parameter *S* = 1000 for the analyses of community phylogenetic structure and diversification (Fig. 5).

**Table 3:** Results of the Likelihood ratio test of constant rate of substitution

	log *L*			
			
	Clock	Nonclock	LR statistic	*P*-value^1^
Chaetodontidae	−413.34	−413.26	0.16	0.681
C1	−61.37	−59.22	4.30	0.035
C2	−258.68	−258.2	0.96	0.327
Pomacentridae	−758.34	−741.50	33.68	<0.001
P1	−185.91	−180.32	11.18	<0.001
P2	−149.90	−149.89	0.02	<0.001
P3	−393.7	−362.49	62.42	<0.001
Labridae	−1114.52	−947.64	333.76	<0.001
L1	−293.51	−282.72	21.58	<0.001
L2	−587.93	−568.13	39.60	<0.001

1 Degree of freedom = 1 (comparing clock-like and nonclock model with one and two rates of substitution, respectively).

**Figure 6:**
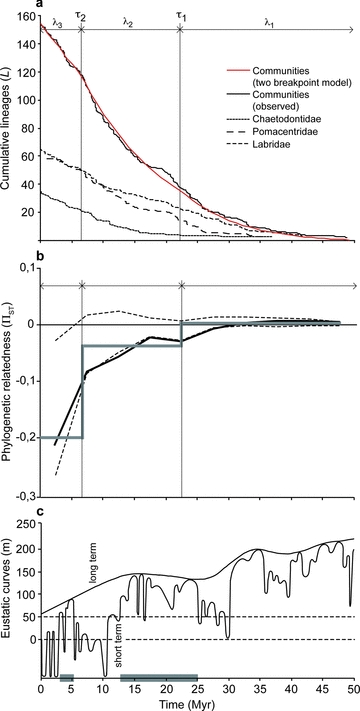
Patterns of diversification and phylogenetic structure in communities from Madagascar, Réunion, and French Polynesia with species age. (A) Phylogenetic relatedness as a function of species age. (B) Cumulative number of lineages through time (LTT) plot for Chaetodontidae, Pomacentridae, Labridae, and all together (communities). Dashed lines define the 95% confidence interval. Observed values for each diversification regime (λ_1_, λ_2_, λ_3_) and 10 Myr windows in light and black lines, respectively. Best-fitted model of diversification for the entire community including three diversification rates (λ_1_, λ_2_, λ_3_) and two breakpoints (τ_1_, τ_2_) in red. (C) Cenozoic long- and short-term eustatic curves (modified from Haq et al. 1987). Times of sea levels 50 m higher than present are considered as high sea levels periods (bars on the time axis) in the sea level model of diversification in Table 4. Changes before 30 Myr are not considered due to the low diversity in extant lineages between 50 Myr and 30 Myr.

The three families provided very similar net diversification rates, overlapping with the estimate from the pooled data, suggesting common diversification regimes (Table 4; Fig. 6). We then tested several alternative models of diversification fitted by ML. Sea levels dramatically fluctuated during the last 50 Myr leading to alternative phases of fragmentation and connectivity (Haq et al. 1987; Fig. 6). Surprisingly, no effect was detected as diversification rates (λ) did not differ between periods of low or high sea levels and a logistic time-dependent model of diversification provided a better fit. Some variations were detected, however, and models incorporating breakpoints provided the best fit, the one including three rates of diversification and two breakpoints being the most likely following AIC scores (Table 4). According to this model, the community diversification rate slowed down twice through time at 22.1 Ma and 6.4 Ma (Fig. 6).

**Table 4:** Parameters estimates for distinct time-dependent models of diversification. AIC = –2log *L*+ 2*k, L* the likelihood score, *k* the number of parameter of the model; Sea levels 50, model of diversification including periods of sea levels 50 m higher than present (λ_>50_, according diversification rate) and remaining periods considered as low sea levels times (λ_<50_, according diversification rate)

Taxon	Model	Parameter estimates ± SD	Log *L*	*k*	AIC
Chaetodontidae	Yule	λ = 0.096 ± 0.012	22.08	1	−42.16
Pomacentridae	Yule	λ = 0.065 ± 0.006	32.84	1	−63.68
Labridae	Yule	λ = 0.059 ± 0.005	36.12	1	−70.24
Communities	Yule	λ = 0.067 ± 0.004	62.27	1	−122.54
Communities	Sea levels 50	λ_>50_ = 0.066 ± 0.007	62.30	2	−120.60
		λ_<50_ = 0.069 ± 0.009			
Communities	Logistic	*a* = –0.028 ± 0.008	67.87	2	−131.74
		*b* = –1.607 ± 0.302			
Communities	One breakpoint	λ_1_ = 0.116 ± 0.018	68.84	3	−131.68
		λ_2_ = 0.058 ± 0.006			
		τ = 21.28 ± 0.002			
Communities	Two breakpoints	λ_3_ = 0.112 ± 0.019	71.68	5	−133.36
		λ_2_ = 0.074 ± 0.008			
		λ_1_ = 0.042 ± 0.006			
		τ_1_ = 22.09 ± 0.003			
		τ_2_ = 6.42 ± 0.003			

The analysis of phylogenetic community structure supported a pattern of phylogenetic clustering when considering the 157 species as shown by a low, but significantly different from zero, positive Π_ST_ among islands (Π_ST_[50] = 0.004; Table 5; Fig. 6). This result is consistent with a long-term effect of geographic isolation on communities’ composition. However, the very low Π_ST_ value indicates that most of the phylogenetic diversity was shared among islands. Nevertheless, the use of partial randomization with respect to absolute divergence thresholds detected a nested but marked pattern of phylogenetic dispersion for the most recently formed species (Π_ST_[5] = –0.214; Table 5; Fig. 6). Species diverged on average by 3.467 Myr within islands but only by 2.854 Myr on average among islands. This result is consistent with a model of allopatric speciation in recent times as the fragmentation of a species range distribution through vicariance leads to sister-species with nonoverlapping distribution, each sister-species co-occurring with phylogenetically more distantly related species (e.g., Barraclough et al. 1998; Barraclough and Vogler 2000). This model recently received support from the study of the evolution of species range distribution arguing that allopatric speciation followed by range change as a consequence of major shifts in species range distribution seems to be the rule in coral reef fish (Quenouille et al. 2011). Worth noting, this model predicts that range overlap is a function of divergence time between species (i.e., higher range overlap between species with higher divergence times), a prediction consistent with the pattern of phylogenetic dispersion detected only in recently formed species. Interestingly, this time-dependent model of community phylogenetic structure matches the shifts in diversification rates since a pattern of phylogenetic dispersion appeared at 22.1 Myr and further clearly establish at 6.4 Myr (Fig. 6).

**Table 5:** Phylogenetic community structure among islands (Réunion, Madagascar, and French Polynesia). Π_ST_ estimates among islands using the chronogram from Figure 5 and species occurrence from Appendix 1. Δ^P^_S_ = mean divergence between distinct species from different sites (here, among species pools of each islands), Δ^P^_T_ = mean divergence between distinct species from the total pool of species (here, all the 157 species sampled in the three islands). Partial randomizations were conducted according to 10 thresholds (each 5 Myr). Obs = mean observed from data, Exp = mean expected after partial randomization, SD = standard deviation, CIinf = inferior 95% confidence interval, CIsup = superior 95% confidence interval, <exp = *P*-value of the one-sided test of Obs < Exp, >exp = *P*-value of the one-sided test of Obs > Exp, ^*^significant one-sided test

	Obs	Exp	SD	CIinf	CIsup	<exp	>exp
Δ^P^_S_(5)	3.467	3.335	0.147	3.036	3.505	0.884	0.216
Δ^P^_T_(5)	2.854	2.866	0.053	2.766	2.976	0.429	0.571
Π_ST_(5)	−0.214	−0.164	0.070	−0.267	−0.029	0.307	0.693
Δ^P^_S_(10)	7.284	7.090	0.190	6.690	7.446	0.856	0.144
Δ^P^_T_(10)	6.721	6.830	0.108	6.616	7.038	0.158	0.842
Π_ST_(10)	−0.083	−0.038	0.026	−0.088	0.017	0.023^*^	0.977
Δ^P^_S_(15)	9.960	9.816	0.189	9.432	10.180	0.772	0.228
Δ^P^_T_(15)	9.406	9.666	0.141	9.372	9.928	0.038^*^	0.962
Π_ST_(15)	−0.058	−0.015	0.018	−0.049	0.024	0.006^*^	0.994
Δ^P^_S_(20)	13.558	13.369	0.197	12.968	13.741	0.827	0.173
Δ^P^_T_(20)	13.237	13.279	0.178	12.946	13.603	0.407	0.593
Π_ST_(20)	−0.024	−0.006	0.009	−0.022	0.012	0.014^*^	0.986
Δ^P^_S_(25)	17.054	16.359	0.279	15.769	16.898	1.000	0.000^*^
Δ^P^_T_(25)	16.548	16.162	0.283	15.599	16.700	0.910	0.090
Π_ST_(25)	−0.030	−0.012	0.009	−0.028	0.005	0.010^*^	0.990
Δ^P^_S_(30)	21.641	21.071	0.256	20.518	21.557	0.992	0.008^*^
Δ^P^_T_(30)	21.476	21.140	0.261	20.575	21.643	0.900	0.100
Π_ST_(30)	−0.007	0.003	0.005	−0.005	0.014	0.004^*^	0.996
Δ^P^_S_(35)	25.236	25.011	0.209	24.592	25.402	0.872	0.128
Δ^P^_T_(35)	25.274	25.120	0.205	24.676	25.518	0.788	0.212
Π_ST_(35)	0.001	0.004	0.004	−0.003	0.013	0.259	0.741
Δ^P^_S_(40)	28.346	28.358	0.238	27.855	28.772	0.470	0.530
Δ^P^_T_(40)	28.488	28.444	0.239	27.946	28.908	0.546	0.454
Π_ST_(40)	0.005	0.003	0.004	−0.004	0.012	0.710	0.290
Δ^P^_S_(45)	31.577	31.779	0.256	31.256	32.234	0.209	0.791
Δ^P^_T_(45)	31.737	31.843	0.263	31.319	32.345	0.340	0.660
Π_ST_(45)	0.005	0.002	0.003	−0.003	0.010	0.839	0.161
Δ^P^_S_(50)^1^	42.585	42.699	0.114	42.461	42.920	0.159	0.841
Δ^P^_T_(50)^1^	42.738	42.701	0.102	42.497	42.896	0.632	0.368
Π_ST_ (50)^1^	0.004	0.001	0.002	−0.002	0.004	0.976	0.024^*^

1 With all species and clades being younger than 50 Ma, correspond to the estimate for the entire dataset including the 157 species.

**Figure 7:**
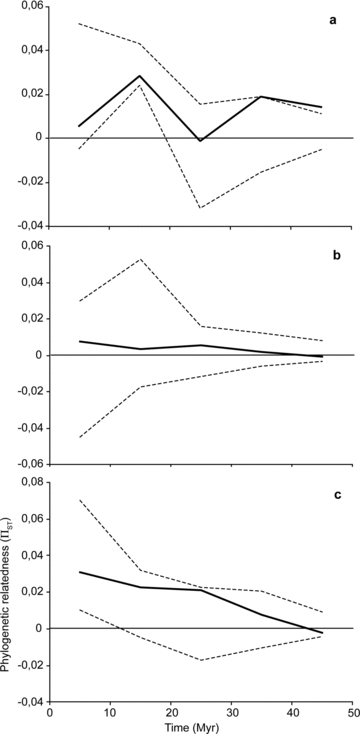
Phylogenetic structure between communities from inner reefs and outer slopes within islands as a function of species age. (A) Réunion. (B) Madagascar. (C) French Polynesia. Communities sampled in inner reefs and outer slopes separated in two distinct groups and communities from each islands analyzed separately. Dashed lines define the 95% confidence interval.

We further explored phylogenetic community structure among communities sampled in the 20 × 20 m^2^ plots in the inner reef and outer slope of Réunion, Madagascar and French Polynesia. Overall, the communities sampled harbored very low similarity since plots distant by a few hundred meters within the same habitat differed to the same extent as those from distinct habitats (Table 6). This pattern was consistently repeated in Réunion and Madagascar and was not unexpected since it has been repeatedly described in the past (Sale 1977, 1978; Sale and Dybdhal 1978; Anderson et al. 1981; Sale and Williams 1982; Sale et al. 1994). We were not able to estimate variability within habitat in French Polynesia, however, according to the sampling designed (see Materials and Methods section). By contrast with the first analysis of phylogenetic community structure among islands, all the communities sampled did not depart from the expectation of random associations at the local scale except in Réunion (Π_ST_[50]; Table 7; Fig. 7). Nevertheless, this pattern was not stable in Réunion and only observed for the two larger divergence thresholds (Table 7). Globally, the Π_ST_ estimates are consistent with a random assembly of species at the local scale in each island. Worth noting, this pattern was remarkably stable across the three islands.

**Table 6:** Summary statistics of community structure from inner reefs and outer slopes sites in Reunion, Madagascar, and French Polynesia according to Appendix 1. Similarity given by the Sørensen index defined as with 2*c*/(*A*+*B*), *c* being the number of common species between two samples, *A* and *B* being the number of species in samples a and b, respectively (Sørensen 1957)

	Réunion	Madagascar	French Polynesia
Inner reef			
Total no. of sites	3	3	1
Total no. of species	39	43	42
Mean no. of species per site	19.7 ± 10.9	17.7 ± 3.56	–
Mean similarity	0.24 ± 0.14	0.22 ± 0.03	–
Outer slope			
Total no. of sites	2	4	1
Total no. of species	48	75	73
Mean no. of species per site	34	30 ± 6.5	–
Mean similarity	0.58	0.31 ± 0.07	–
Total			
Total no of species	67	86	90
Mean similarity^1^	0.23 ± 0.14	0.20 ± 0.08	0.43

1 Mean similarity from pairwise comparisons of sites from inner reef and outer slope.

**Table 7:** Phylogenetic community structure between inner reef and outer slope sites within island (Réunion, Madagascar, and French Polynesia). Π_ST_ estimates using the chronogram from Figure 5 and species occurence from Appendix 1. Δ^P^_S_ = mean divergence between distinct species from different sites (here, among communities from the inner reef and outer slope), Δ^P^_T_ = mean divergence between distinct species from the total pool of species (here, all the species sampled in the island). Partial randomizations were conducted according to five thresholds (each 10 Myr). Obs = mean observed from data, Exp = mean expected after partial randomization, SD = standard deviation, CIinf = inferior 95% confidence interval, CIsup = superior 95% confidence interval, <exp = *P*-value of the one-sided test of Obs < Exp, >exp = *P*-value of the one-sided test of Obs > Exp, ^*^significant one-sided test

		Obs	Exp	SD	CIinf	CIsup	<exp	>exp
Madagascar	Δ^P^_S_(10)	7.548	7.669	0.093	7.491	7.825	0.101	0.899
	Δ^P^_T_(10)	7.602	7.570	0.139	7.357	7.858	0.601	0.399
	Π_ST_(10)	0.007	−0.014	0.021	−0.045	0.029	0.839	0.171
	Δ^P^_S_(20)	12.144	12.092	0.254	11.569	12.610	0.590	0.410
	Δ^P^_T_(20)	12.183	12.267	0.292	11.734	12.878	0.438	0.562
	Π_ST_(20)	0.003	0.014	0.017	−0.018	0.052	0.280	0.720
	Δ^P^_S_(30)	22.043	22.269	0.291	21.632	22.775	0.217	0.783
	Δ^P^_T_(30)	22.157	22.290	0.260	21.753	22.773	0.298	0.702
	Π_ST_(30)	0.005	0.001	0.007	−0.012	0.016	0.732	0.268
	Δ^P^_S_(40)	28.838	28.884	0.247	28.361	29.325	0.395	0.605
	Δ^P^_T_(40)	28.893	28.914	0.247	28.456	29.387	0.468	0.532
	Π_ST_(40)	0.002	0.001	0.005	−0.006	0.012	0.640	0.360
	Δ^P^_S_(50)^1^	42.096	42.211	0.192	41.773	42.530	0.259	0.741
	Δ^P^_T_(50)^1^	42.062	42.214	0.167	41.884	42.556	0.184	0.816
	Π_ST_(50)^1^	−0.001	<0.001	0.003	−0.003	0.008	0.470	0.530
	Δ^P^_S_(10)	7.156	7.191	0.035	7.156	7.241	0.116	0.984
	Δ^P^_T_(10)	7.197	7.321	0.136	7.197	7.559	0.148	0.952
	Π_ST_(10)	0.006	0.017	0.019	−0.005	0.052	0.300	0.700
	Δ^P^_S_(20)	14.650	14.588	0.172	14.159	14.875	0.587	0.413
	Δ^P^_T_(20)	15.079	15.070	0.185	14.637	15.387	0.477	0.523
	Π_ST_(20)	0.029	0.032	0.005	0.024	0.043	0.260	0.740
	Δ^P^_S_(30)	20.367	20.642	0.155	20.314	20.933	0.051	0.949
Réunion	Δ^P^_T_(30)	20.335	20.391	0.234	19.981	20.882	0.425	0.575
	Π_ST_(30)	−0.001	−0.012	0.012	−0.032	0.016	0.815	0.185
	Δ^P^_S_(40)	27.105	28.126	0.267	27.491	28.565	0.002^*^	0.998
	Δ^P^_T_(40)	27.630	28.087	0.308	27.456	28.688	0.067	0.933
	Π_ST_(40)	0.019	−0.001	0.009	−0.015	0.019	0.977	0.023^*^
	Δ^P^_S_(50)^1^	42.079	42.618	0.157	42.286	42.889	0.005^*^	0.995
	Δ^P^_T_(50)^1^	42.678	42.623	0.142	42.371	42.970	0.672	0.323
	Π_ST_(50)^1^	0.014	<0.001	0.004	−0.005	0.011	0.985	0.015^*^
French Polynesia	Δ^P^_S_(10)	7.203	6.893	0.206	6.467	7.290	0.925	0.075
	Δ^P^_T_(10)	7.432	7.198	0.165	6.805	7.528	0.958	0.042
	Π_ST_(10)	0.031	0.042	0.017	0.010	0.070	0.214	0.786
	Δ^P^_S_(20)	13.400	13.613	0.223	13.153	14.026	0.184	0.816
	Δ^P^_T_(20)	13.711	13.769	0.208	13.389	14.176	0.400	0.600
	Π_ST_(20)	0.022	0.011	0.010	−0.005	0.032	0.883	0.117
	Δ^P^_S_(30)	19.749	20.274	0.325	19.563	20.835	0.066	0.934
	Δ^P^_T_(30)	20.172	20.260	0.251	19.748	20.755	0.365	0.635
	Π_ST_(30)	0.021	−0.001	0.010	−0.017	0.023	0.973	0.027
	Δ^P^_S_(40)	26.845	27.287	0.405	26.373	27.973	0.140	0.860
	Δ^P^_T_(40)	27.047	27.331	0.350	26.625	28.023	0.213	0.787
	Π_ST_(40)	0.008	0.002	0.008	−0.011	0.021	0.810	0.190
	Δ^P^_S_(50)^1^	42.804	42.910	0.143	42.592	43.156	0.212	0.788
	Δ^P^_T_(50)^1^	42.699	42.912	0.130	42.667	43.188	0.047	0.953
	Π_ST_(50)^1^	−0.002	<0.001	0.003	−0.004	0.009	0.245	0.755

1 With all species and clades being younger than 50 Ma, correspond to the global estimate for the entire community.

## Discussion

### Phylogenetic community structure and spatial scales

Initially proposed by Webb (2000), the analysis of phylogenetic community structure is based on the assumption that, because organisms interact via their phenotypes, and because phenotypes are not randomly distributed with respect to phylogeny, we should expect that the phylogenetic composition of a community is partially the product of species interactions (Webb et al. 2002; Emerson and Gillespie 2008; Vamosi et al. 2009; Ackerly 2010). In a niche-based view (Table 1), fluctuations in species richness within communities is likely to affect species interactions since diversity influences the probability of having better competitors in the community (Schluter and McPhail 1992; Gillespie 2004; Ackerly et al. 2006; Ackerly 2010). In this context, ecological mechanisms behind species interactions including habitat filtering, competitive exclusion, mutualism, or facilitation might themselves apply differently across descendant clades (McPeek 2008; Vamosi et al. 2009). Here, the use of a threshold-based approach confirmed that phylogenetic community structure might not be constant and helped detect nested patterns.

Shifts in phylogenetic community structure, however, raise the question of the ecological mechanisms driving community assembly, and previous authors pointed to the importance of spatial scale in detecting phylogenetic community structure (Cavender-Barres et al. 2006; Swenson et al. 2006; Vamosi et al. 2009). The main challenge of considering several geographic scales consists in disentangling the drivers preventing species co-occurrence ranging from geographic isolation resulting from historical factors at the regional scale (e.g., allopatric speciation) to ecological mechanisms at local scale (e.g., habitat filtering, competitive exclusion). As a consequence, both competitive exclusion and allopatric speciation can lead to the confinement of species in alternative sites depending on scale and influence phylogenetic community structure (Vamosi et al. 2009).

Several observations support that the present pattern of phylogenetic dispersion among islands for the most recently derived species in the phylogeny result from allopatric speciation and not from competitive exclusion. First, evolutionary imprints of ecological mechanisms derived from individual interactions such as competitive exclusion are supposed to be effective among contiguous patches of alternative habitats (Losos et al. 1998; Ricklefs and Bermingham 2001; Gillespie 2004; Gavrilets and Vose 2005). The lack of significant structure, here, at the local scale argues for a distinct origin of the regional structure. Second, Indian and Pacific oceans have been repeatedly isolated during the last 5 Myr and particularly during the climatic fluctuations of the Pleistocene as sea levels frequently dropped by more than 100 m during glacial times leading to the formation of a land bridge across the Sunda shelf (Indonesia, Malaysia and Philippines archipelago) and isolating marine communities on each side (Randall 1998; Barber et al. 2000; Rocha and Bowen 2008). Finally, recent studies on dispersal and recruitment in coral reef fish argue for much restricted dispersal than previously thought despite the long-lasting pelagic larval stages making long-distance dispersal unlikely in coral reef fish (e.g., Doherty et al. 1995; Jones et al. 1999; Almany et al. 2007).

The shift in phylogenetic community structure among islands toward phylogenetic dispersion emphasizes that marine barriers to dispersal fluctuate in effectiveness through times (Rocha and Bowen 2008). A relationship between species range overlap and species divergence times has been recently detected in Pomacentrid fish and Labrid fish as expected following a model of allopatric speciation and evolution of species ranges through major shifts (Quenouille et al. 2011). The present pattern is consistent with this model since most of the lineages exhibiting alternative distribution (i.e., nonoverlapping ranges) at the regional scale are younger than 5 Myr and this pattern of phylogenetic dispersion abruptly disappears when considering older lineages. Worth noting, Quenouille et al. (2011) pointed to a large shift in species range overlap between 4 Myr and 5 Myr leading to an increased co-occurrence of lineages regionally, as described here. Sea levels fluctuations, particularly the last episode of marine highstand between 4 Myr and 5 Myr (Fig. 6), proceeding to variations in the extent of emerged lands and land bridges constitute a good example of such fluctuations that can influence the effectiveness of barrier to dispersal.

The present study confirms the importance of scales when considering communities phylogenetic structure since alternative patterns may be nested on distinct spatial and temporal scales (Vamosi et al., 2009). The pattern detected at the local scale confirmed a scale dependency of the phylogenetic structure since all three islands supported independently a model of random community assembly, with no phylogenetic structure between the inner reef and the outer slope, by contrast with the pattern of phylogenetic dispersion among islands. Overall, the assembly of species in communities across habitats departs from a deterministic model of species assembly through phylogenetic relatedness and competitive interactions, a result consistent with a model of random association (Sale 1977, 1978; Chesson and Warner 1981; Warner and Chesson 1985).

Habitat filtering relies on ecological trade-offs for traits associated with the use of alternative resources differentially distributed in landscapes (Schluter and McPhail 1992). The lack of significant departure of the community phylogenetic structure from a model of random assembly may either mean that life-history strategies are not correlated with phylogenetic distance (i.e., phylogenetic conservatism of traits does not hold for coral reef fish), or that mechanisms promoting species coexistence are not based on niche specialization. The first hypothesis seems very unlikely since recent phylogenetic studies suggest that traits such as trophic strategies are generally conserved and major trophic shifts in the phylogeny correspond to large evolutionary steps seldom crossed during lineages’ evolution (Streelman et al. 2002; Rüber et al. 2003; Alfaro et al. 2007; Fessler and Westneat 2007). By contrast, ecosystems with benign but frequent and unpredictable perturbations may be expected to favor species with the broader ecological requirements since ecological specialization reduces the opportunity to find suitable sites that are randomly available (Sale 1977, 1978). Thus, the present pattern of phylogenetic structure may be seen as an ecological consequence of a random model of community assembly promoting communities of generalist species rather than the lack of imprint of ecological mechanisms on community assembly and phylogenetic structure.

### Community assembly and diversification

Communities are the product of biotic and landscapes fluctuations over evolutionary times (Ricklefs 1987; Ricklefs and Schluter 1993). Lineages diversify and supply communities with species that arrange themselves in assemblies as a consequence of ecological constraints (i.e., habitat preferences, competitive exclusion, stochastic perturbations) and macroevolutionary processes (i.e., speciation, extinction) proceeding to the sorting of species in communities (Johnson and Stichcombe 2007; Emerson and Gillespie 2008). Ecological and evolutionary processes were thought to act on distinct spatial scale; however, due to large differences in the timescales involved in speciation (i.e., Million years) and biotic interactions (i.e., a few tens of generations).

However, several examples highlighted that the ecological dynamics of community assembly might contribute to determine the evolutionary processes that drive diversification (Harvey and Pagel 1991; Ricklefs and Schluter 1993; Barraclough and Nee 2001; McPeek 2008; Rabosky 2009). First, a lineage may become extinct as a consequence of physical perturbations (e.g., climate change, volcanic activity, sea level fluctuations) or interactions with other lineages (McPeek 2008), and the extinction of a lineage may open new ecological opportunities and foster the diversification of other lineages (Schluter 2000; Gavrilets and Vose 2005). Second, a clade may initially diversify in relatively empty ecological space as a consequence of the colonization of novel habitats or the evolution of key innovations and foster community assembly through adaptive radiation (Sanderson and Donoghue 1994; Losos et al. 1998; Schluter 2000; Ricklefs and Bermingham 2001; Gillespie 2004; Gavrilets and Vose 2005; Ackerly et al. 2006; Ackerly 2010).

Sea level changes are such major physical perturbations able to foster communities rearrangements through macroevolutionary processes (i.e., speciation and extinction). The phylogenetic community structure detected here among islands is consistent with an important step of diversification through allopatric speciation during the last 5 Myr. Sea level changes have been hypothesized to promote allopatric speciation through vicariance on each side of the Sunda shelf during glacial times with low sea levels (Barber et al. 2000; Rocha and Bowen 2008), a hypothesis that predicts temporal changes in diversification rates through increased rates of speciation related to sea level changes. Likewise, sea level rises may be expected to increase the extinction rate, as a 150-m highstand will submerge most oceanic islands from the Indian and Pacific oceans and rarefy suitable habitats for coral reef fish in open areas (Briggs 1974). Slowdowns in diversification rate may result from either a decreasing of speciation or increasing of extinction through time, both scenarios being difficult to distinguish in practice (Kubo and Iwasa 1995; Rabosky and Lovette 2008; Stadler 2009). The likelihood analysis of alternative diversification patterns, however, found no evidence for an influence of long-lasting sea level changes on diversification (Table 4).

By contrast, the present pattern of diversification provided an intriguing insight to the interplay between community assembly and diversification. In a niche-based view (Table 1), diversification can be seen as the filling of landscapes ecological space through the production of new species that insert in the ecological system (Webb et al. 2002). Following this hypothesis, speciation rate can be high as lineages ecologically diversify and fill the available ecological space but speciation rate should slowdown as more niches become occupied (Schluter 2000; Gavrilets and Vose 2005; McPeek 2008; Phillimore and Price 2008; Rabosky 2009). Decreases in diversification rate have been recently described, including in marine fish, and have been systematically described as a consequence of the saturation of the ecological space and increased biotic interactions including competitive exclusion (Johns and Avise 1998; Rüber et al. 2003; Barber and Bellwood 2005; Rüber and Zardoya 2005; Read et al. 2006; Alfaro et al. 2007; Philimore and Price 2008; reviewed in McPeek 2008 and Rabosky 2009). In our study, a decrease in diversification rate, consistent with a niche-based view of diversification, was found in agreement with previous studies (Table 1). According to the niche theory, competitive exclusion promote resource partitioning and ecological specialization (Schluter 2000), a trend that may be expected to produce frequent ecological shifts during lineage evolution to foster the filling of the ecological space (Losos et al. 1998; Ricklefs and Bermingham 2001; Gillespie 2004; Gavrilets and Vose 2005; Duponchelle et al. 2008). Nevertheless, this expectation is challenged by recent phylogenetic studies on marine fish supporting that ecological shifts have been rather rare during the diversification of several lineages of coral reef fish as exemplified by the scarcity of trophic shifts (Streelman et al. 2002; Rüber et al. 2003; Alfaro et al. 2007; Fessler and Westneat 2007). Similarly, our results on phylogenetic community structure at the local scale challenge this view since the lack of significant departure from a random community assembly model rather supports a neutral than a niche model of species coexistence. The lottery model of community assembly predicts that ecological specialization is not favored in a stochastic environment. By contrast, the unpredictability of the supply of living space favors sedentary species, which breed often, produce numerous clutches of dispersive eggs or larvae, and have broad ecological requirements (Sale 1977, 1978). In this context, the supply of suitable living space is more restrictive than the amount of trophic resources by itself (Almany 2004). In this case, competition may occur without leaving an evolutionary imprint of competitive exclusion in community phylogenetic structure, since competition occurs for living space and not for resources. Nevertheless, this model may conduct to a pattern of decreasing diversification still, providing that the supply of living space is limited as expected in a finite island model (MacArthur and Wilson 1967).

## Conclusion

Overall, the phylogenetic community structure at local scale is consistent with the prediction from the neutral model of community assembly as proposed initially by Sale (1977) for coral reef fish and Hubbell (2001) for tropical forest trees. Nevertheless, Sale's lottery model of community assembly predicts that the stochastic and unpredictable nature of coral reefs prevents species loss in communities by favoring generalist and sedentary species with restricted individual living space that minimize interactions among adult individuals. Likewise, stochastic perturbations will likely prevent communities to reach equilibrium between extinction and immigration in interacting species assemblage, and competitive exclusion may take tens of generations to complete dominance in such a system (Sale 1977). By contrast, Hubbell's neutral model does not advocate such stabilizing effect of stochastic perturbations on coexistence.

According to both the lottery and the ecological drift model, species–area relationships are a consequence of spatially limited supply of living space. Nevertheless, the implications for speciation and extinction rates are not trivial. The present pattern suggests that available living space is not only of importance for coexistence but also has evolutionary consequences on diversification. The decrease in diversification rates in noninteracting species assemblages may result from the saturation of space without consideration for the available ecological space. In this context, species richness may increase through diversification up to a stationary state in communities as a function of species-area relationships. According to this hypothesis, the heterogeneous distribution of species richness in Indo-Pacific coral reef fish communities may be a simple consequence of species–area relationships.

## Appendix

**Appendix 1:** Sampling records for the 157 species of Chaetodontidae, Pomacentridae, and Labridae included in the present study. Data include habitat, depth, geo-references, and date. Depth provided in meters

Units	Region	Locality	Site	Site code	Genus	Species	Habitat	Min	Max	Latitude	Longitude	Date
MAD01	Madagascar	Nosy Be	Nosy Tanikely	MGNW-01	*Chaetodon*	*falcula*	Outer slope	3	10	13°47722¢S	48°23861¢E	07/05/2008
MAD01	Madagascar	Nosy Be	Nosy Tanikely	MGNW-01	*Chaetodon*	*trifasciatus*	Outer slope	3	10	13°47722¢S	48°23861¢E	07/05/2008
MAD01	Madagascar	Nosy Be	Nosy Tanikely	MGNW-01	*Heniochus*	*acuminatus*	Outer slope	3	10	13°47722¢S	48°23861¢E	07/05/2008
MAD01	Madagascar	Nosy Be	Nosy Tanikely	MGNW-01	*Anampses*	*twistii*	Outer slope	3	10	13°47722¢S	48°23861¢E	07/05/2008
MAD01	Madagascar	Nosy Be	Nosy Tanikely	MGNW-01	*Bodianus*	*axillaris*	Outer slope	3	10	13°47722¢S	48°23861¢E	07/05/2008
MAD01	Madagascar	Nosy Be	Nosy Tanikely	MGNW-01	*Cheilinus*	*oxycephalus*	Outer slope	3	10	13°47722¢S	48°23861¢E	07/05/2008
MAD01	Madagascar	Nosy Be	Nosy Tanikely	MGNW-01	*Cheilinus*	*oxycephalus*	Outer slope	3	10	13°47722¢S	48°23861¢E	07/05/2008
MAD01	Madagascar	Nosy Be	Nosy Tanikely	MGNW-01	*Cheilio*	*inermis*	Outer slope	3	10	13°47722¢S	48°23861¢E	07/05/2008
MAD01	Madagascar	Nosy Be	Nosy Tanikely	MGNW-01	*Epibulus*	*insidiator*	Outer slope	3	10	13°47722¢S	48°23861¢E	07/05/2008
MAD01	Madagascar	Nosy Be	Nosy Tanikely	MGNW-01	*Epibulus*	*insidiator*	Outer slope	3	10	13°47722¢S	48°23861¢E	07/05/2008
MAD01	Madagascar	Nosy Be	Nosy Tanikely	MGNW-01	*Gomphosus*	*caeruleus*	Outer slope	3	10	13°47722¢S	48°23861¢E	07/05/2008
MAD01	Madagascar	Nosy Be	Nosy Tanikely	MGNW-01	*Halichoeres*	*hortulanus*	Outer slope	3	10	13°47722¢S	48°23861¢E	07/05/2008
MAD01	Madagascar	Nosy Be	Nosy Tanikely	MGNW-01	*Labroides*	*dimidiatus*	Outer slope	3	10	13°47722¢S	48°23861¢E	07/05/2008
MAD01	Madagascar	Nosy Be	Nosy Tanikely	MGNW-01	*Oxycheilinus*	*digramma*	Outer slope	3	10	13°47722¢S	48°23861¢E	07/05/2008
MAD01	Madagascar	Nosy Be	Nosy Tanikely	MGNW-01	*Thalassoma*	*hebraicum*	Outer slope	3	10	13°47722¢S	48°23861¢E	07/05/2008
MAD01	Madagascar	Nosy Be	Nosy Tanikely	MGNW-01	*Thalassoma*	*lunare*	Outer slope	3	10	13°47722¢S	48°23861¢E	07/05/2008
MAD01	Madagascar	Nosy Be	Nosy Tanikely	MGNW-01	*Wetmorella*	*nigropinnata*	Outer slope	3	10	13°47722¢S	48°23861¢E	07/05/2008
MAD01	Madagascar	Nosy Be	Nosy Tanikely	MGNW-01	*Abudefduf*	*septemfasciatus*	Outer slope	3	10	13°47722¢S	48°23861¢E	07/05/2008
MAD01	Madagascar	Nosy Be	Nosy Tanikely	MGNW-01	*Amblyglyphidodon*	*indicus*	Outer slope	3	10	13°47722¢S	48°23861¢E	07/05/2008
MAD01	Madagascar	Nosy Be	Nosy Tanikely	MGNW-01	*Amphiprion*	*akallopisos*	Outer slope	3	10	13°47722¢S	48°23861¢E	07/05/2008
MAD01	Madagascar	Nosy Be	Nosy Tanikely	MGNW-01	*Chromis*	*atripectoralis*	Outer slope	3	10	13°47722¢S	48°23861¢E	07/05/2008
MAD01	Madagascar	Nosy Be	Nosy Tanikely	MGNW-01	*Chromis*	*dimidiata*	Outer slope	3	10	13°47722¢S	48°23861¢E	07/05/2008
MAD01	Madagascar	Nosy Be	Nosy Tanikely	MGNW-01	*Chromis*	*ternatensis*	Outer slope	3	10	13°47722¢S	48°23861¢E	07/05/2008
MAD01	Madagascar	Nosy Be	Nosy Tanikely	MGNW-01	*Chromis*	*xanthochira*	Outer slope	3	10	13°47722¢S	48°23861¢E	07/05/2008
MAD01	Madagascar	Nosy Be	Nosy Tanikely	MGNW-01	*Chrysiptera*	*brownriggii*	Outer slope	3	10	13°47722¢S	48°23861¢E	07/05/2008
MAD01	Madagascar	Nosy Be	Nosy Tanikely	MGNW-01	*Dascyllus*	*trimaculatus*	Outer slope	3	10	13°47722¢S	48°23861¢E	07/05/2008
MAD01	Madagascar	Nosy Be	Nosy Tanikely	MGNW-01	*Neoglyphidodon*	*melas*	Outer slope	3	10	13°47722¢S	48°23861¢E	07/05/2008
MAD01	Madagascar	Nosy Be	Nosy Tanikely	MGNW-01	*Plectroglyphidodon*	*lacrymatus*	Outer slope	3	10	13°47722¢S	48°23861¢E	07/05/2008
MAD01	Madagascar	Nosy Be	Nosy Tanikely	MGNW-01	*Pomacentrus*	*baenschi*	Outer slope	3	10	13°47722¢S	48°23861¢E	07/05/2008
MAD01	Madagascar	Nosy Be	Nosy Tanikely	MGNW-01	*Pomacentrus*	*caeruleus*	Outer slope	3	10	13°47722¢S	48°23861¢E	07/05/2008
MAD01	Madagascar	Nosy Be	Nosy Tanikely	MGNW-01	*Pomacentrus*	*pavo*	Outer slope	3	10	13°47722¢S	48°23861¢E	07/05/2008
MAD01	Madagascar	Nosy Be	Nosy Tanikely	MGNW-01	*Pomacentrus*	*sulfureus*	Outer slope	3	10	13°47722¢S	48°23861¢E	07/05/2008
MAD01	Madagascar	Nosy Be	Nosy Tanikely	MGNW-01	*Pomacentrus*	*trichrous*	Outer slope	3	10	13°47722¢S	48°23861¢E	07/05/2008
MAD01	Madagascar	Nosy Be	Bay d'Ambanoro	MGNW-07	*Chaetodon*	*bennetti*	Outer slope	4	10	13°40806¢S	48°30111¢E	10/05/2008
MAD01	Madagascar	Nosy Be	Bay d'Ambanoro	MGNW-07	*Hemigymnus*	*melanopterus*	Outer slope	4	10	13°40806¢S	48°30111¢E	10/05/2008
MAD01	Madagascar	Nosy Be	Nosy Hotel	MGNW-20	*Halichoeres*	*nebulosus*	Outer slope	3	6	13°42444¢S	48°36417¢E	15/05/2008
MAD01	Madagascar	Nosy Be	Nosy Hotel	MGNW-20	*Hemigymnus*	*fasciatus*	Outer slope	3	6	13°42444¢S	48°36417¢E	15/05/2008
MAD02	Madagascar	Nosy Be	Nosy Tanikely	MGNW-10	*Chaetodon*	*lunula*	Outer slope	10	15	13°48333¢S	48°23333¢E	11/05/2008
MAD02	Madagascar	Nosy Be	Nosy Tanikely	MGNW-10	*Chaetodon*	*trifascialis*	Outer slope	10	15	13°48333¢S	48°23333¢E	11/05/2008
MAD02	Madagascar	Nosy Be	Nosy Tanikely	MGNW-10	*Chaetodon*	*trifasciatus*	Outer slope	10	15	13°48333¢S	48°23333¢E	11/05/2008
MAD02	Madagascar	Nosy Be	Nosy Tanikely	MGNW-10	*Heniochus*	*acuminatus*	Outer slope	10	15	13°48333¢S	48°23333¢E	11/05/2008
MAD02	Madagascar	Nosy Be	Nosy Tanikely	MGNW-10	*Anampses*	*caeruleopunctatus*	Outer slope	10	15	13°48333¢S	48°23333¢E	11/05/2008
MAD02	Madagascar	Nosy Be	Nosy Tanikely	MGNW-10	*Anampses*	*twistii*	Outer slope	10	15	13°48333¢S	48°23333¢E	11/05/2008
MAD02	Madagascar	Nosy Be	Nosy Tanikely	MGNW-10	*Cheilinus*	*fasciatus*	Outer slope	10	15	13°48333¢S	48°23333¢E	11/05/2008
MAD02	Madagascar	Nosy Be	Nosy Tanikely	MGNW-10	*Cheilinus*	*oxycephalus*	Outer slope	10	15	13°48333¢S	48°23333¢E	11/05/2008
MAD02	Madagascar	Nosy Be	Nosy Tanikely	MGNW-10	*Epibulus*	*insidiator*	Outer slope	10	15	13°48333¢S	48°23333¢E	11/05/2008
MAD02	Madagascar	Nosy Be	Nosy Tanikely	MGNW-10	*Halichoeres*	*marginatus*	Outer slope	10	15	13°48333¢S	48°23333¢E	11/05/2008
MAD02	Madagascar	Nosy Be	Nosy Tanikely	MGNW-10	*Halichoeres*	*dussumieri*	Outer slope	10	15	13°48333¢S	48°23333¢E	11/05/2008
MAD02	Madagascar	Nosy Be	Nosy Tanikely	MGNW-10	*Halichoeres*	*hortulanus*	Outer slope	10	15	13°48333¢S	48°23333¢E	11/05/2008
MAD02	Madagascar	Nosy Be	Nosy Tanikely	MGNW-10	*Labroides*	*dimidiatus*	Outer slope	10	15	13°48333¢S	48°23333¢E	11/05/2008
MAD02	Madagascar	Nosy Be	Nosy Tanikely	MGNW-10	*Oxycheilinus*	*digramma*	Outer slope	10	15	13°48333¢S	48°23333¢E	11/05/2008
MAD02	Madagascar	Nosy Be	Nosy Tanikely	MGNW-10	*Stethojulis*	*albovittata*	Outer slope	10	15	13°48333¢S	48°23333¢E	11/05/2008
MAD02	Madagascar	Nosy Be	Nosy Tanikely	MGNW-10	*Thalassoma*	*amblycephalum*	Outer slope	10	15	13°48333¢S	48°23333¢E	11/05/2008
MAD02	Madagascar	Nosy Be	Nosy Tanikely	MGNW-10	*Thalassoma*	*hardwicke*	Outer slope	10	15	13°48333¢S	48°23333¢E	11/05/2008
MAD02	Madagascar	Nosy Be	Nosy Tanikely	MGNW-10	*Thalassoma*	*hebraicum*	Outer slope	10	15	13°48333¢S	48°23333¢E	11/05/2008
MAD02	Madagascar	Nosy Be	Nosy Tanikely	MGNW-10	*Thalassoma*	*hebraicum*	Outer slope	10	15	13°48333¢S	48°23333¢E	11/05/2008
MAD02	Madagascar	Nosy Be	Nosy Tanikely	MGNW-10	*Abudefduf*	*sordidus*	Outer slope	10	15	13°48333¢S	48°23333¢E	11/05/2008
MAD02	Madagascar	Nosy Be	Nosy Tanikely	MGNW-10	*Abudefduf*	*sparoides*	Outer slope	10	15	13°48333¢S	48°23333¢E	11/05/2008
MAD02	Madagascar	Nosy Be	Nosy Tanikely	MGNW-10	*Amblyglyphidodon*	*indicus*	Outer slope	10	15	13°48333¢S	48°23333¢E	11/05/2008
MAD02	Madagascar	Nosy Be	Nosy Tanikely	MGNW-10	*Amphiprion*	*akallopisos*	Outer slope	10	15	13°48333¢S	48°23333¢E	11/05/2008
MAD02	Madagascar	Nosy Be	Nosy Tanikely	MGNW-10	*Chromis*	*ternatensis*	Outer slope	10	15	13°48333¢S	48°23333¢E	11/05/2008
MAD02	Madagascar	Nosy Be	Nosy Tanikely	MGNW-10	*Chromis*	*xanthochira*	Outer slope	10	15	13°48333¢S	48°23333¢E	11/05/2008
MAD02	Madagascar	Nosy Be	Nosy Tanikely	MGNW-10	*Chrysiptera*	*annulata*	Outer slope	10	15	13°48333¢S	48°23333¢E	11/05/2008
MAD02	Madagascar	Nosy Be	Nosy Tanikely	MGNW-10	*Chrysiptera*	*brownriggii*	Outer slope	10	15	13°48333¢S	48°23333¢E	11/05/2008
MAD02	Madagascar	Nosy Be	Nosy Tanikely	MGNW-10	*Dascyllus*	*carneus*	Outer slope	10	15	13°48333¢S	48°23333¢E	11/05/2008
MAD02	Madagascar	Nosy Be	Nosy Tanikely	MGNW-10	*Dascyllus*	*trimaculatus*	Outer slope	10	15	13°48333¢S	48°23333¢E	11/05/2008
MAD02	Madagascar	Nosy Be	Nosy Tanikely	MGNW-10	*Neoglyphidodon*	*melas*	Outer slope	10	15	13°48333¢S	48°23333¢E	11/05/2008
MAD02	Madagascar	Nosy Be	Nosy Tanikely	MGNW-10	*Neopomacentrus*	*cyanomos*	Outer slope	10	15	13°48333¢S	48°23333¢E	11/05/2008
MAD02	Madagascar	Nosy Be	Nosy Tanikely	MGNW-10	*Plectroglyphidodon*	*leucozonus*	Outer slope	10	15	13°48333¢S	48°23333¢E	11/05/2008
MAD02	Madagascar	Nosy Be	Nosy Tanikely	MGNW-10	*Pomacentrus*	*caeruleus*	Outer slope	10	15	13°48333¢S	48°23333¢E	11/05/2008
MAD02	Madagascar	Nosy Be	Nosy Tanikely	MGNW-10	*Pomacentrus*	*pavo*	Outer slope	10	15	13°48333¢S	48°23333¢E	11/05/2008
MAD02	Madagascar	Nosy Be	Nosy Tanikely	MGNW-10	*Pomacentrus*	*sulfureus*	Outer slope	10	15	13°48333¢S	48°23333¢E	11/05/2008
MAD02	Madagascar	Nosy Be	Nosy Tanikely	MGNW-10	*Pomacentrus*	*trichrous*	Outer slope	10	15	13°48333¢S	48°23333¢E	11/05/2008
MAD02	Madagascar	Nosy Be	Nosy Tanikely	MGNW-39	*Chaetodon*	*zanzibariensis*	Outer slope	20	25	13°45716¢S	48°24840¢E	21/05/2008
MAD03	Madagascar	Nosy Be	Ampasindava	MGNW-12	*Chaetodon*	*trifasciatus*	Inner reef	1	4	13°38111¢S	48°35028¢E	12/05/2008
MAD03	Madagascar	Nosy Be	Ampasindava	MGNW-12	*Cheilinus*	*fasciatus*	Inner reef	1	4	13°38111¢S	48°35028¢E	12/05/2008
MAD03	Madagascar	Nosy Be	Ampasindava	MGNW-12	*Cheilinus*	*oxycephalus*	Inner reef	1	4	13°38111¢S	48°35028¢E	12/05/2008
MAD03	Madagascar	Nosy Be	Ampasindava	MGNW-12	*Epibulus*	*insidiator*	Inner reef	1	4	13°38111¢S	48°35028¢E	12/05/2008
MAD03	Madagascar	Nosy Be	Ampasindava	MGNW-12	*Stethojulis*	*strigiventer*	Inner reef	1	4	13°38111¢S	48°35028¢E	12/05/2008
MAD03	Madagascar	Nosy Be	Ampasindava	MGNW-12	*Amphiprion*	*akallopisos*	Inner reef	1	4	13°38111¢S	48°35028¢E	12/05/2008
MAD03	Madagascar	Nosy Be	Ampasindava	MGNW-12	*Chromis*	*atripectoralis*	Inner reef	1	4	13°38111¢S	48°35028¢E	12/05/2008
MAD03	Madagascar	Nosy Be	Ampasindava	MGNW-12	*Pomacentrus*	*trilineatus*	Inner reef	1	4	13°38111¢S	48°35028¢E	12/05/2008
MAD03	Madagascar	Nosy Be	Ampasindava	MGNW-12	*Chrysiptera*	*unimaculata*	Inner reef	1	4	13°38111¢S	48°35028¢E	12/05/2008
MAD03	Madagascar	Nosy Be	Ampasipohy	MGNW-13	*Halichoeres*	*dussumieri*	Inner reef	1	4	13°42000¢S	48°30806¢E	12/05/2008
MAD03	Madagascar	Nosy Be	Ampasipohy	MGNW-13	*Amphiprion*	*akallopisos*	Inner reef	1	4	13°42000¢S	48°30806¢E	12/05/2008
MAD03	Madagascar	Nosy Be	Ampasipohy	MGNW-13	*Chrysiptera*	*unimaculata*	Inner reef	1	4	13°42000¢S	48°30806¢E	12/05/2008
MAD03	Madagascar	Nosy Be	Nosy Sakatia	MGNW-14	*Chrysiptera*	*unimaculata*	Inner reef	1	4	13°42000¢S	48°30806¢E	12/05/2008
MAD03	Madagascar	Nosy Be	Ampasindava	MGNW-15	*Cheilio*	*inermis*	Inner reef	1	4	13°42000¢S	48°30806¢E	12/05/2008
MAD03	Madagascar	Nosy Be	Ampasindava	MGNW-15	*Amphiprion*	*akallopisos*	Inner reef	1	4	13°42000¢S	48°30806¢E	12/05/2008
MAD03	Madagascar	Nosy Be	Ampasindava	MGNW-15	*Chromis*	*atripectoralis*	Inner reef	1	4	13°42000¢S	48°30806¢E	12/05/2008
MAD03	Madagascar	Nosy Be	Ampasindava	MGNW-15	*Chromis*	*viridis*	Inner reef	1	4	13°42000¢S	48°30806¢E	12/05/2008
MAD03	Madagascar	Nosy Be	Ampasindava	MGNW-15	*Dascyllus*	*aruanus*	Inner reef	1	4	13°42000¢S	48°30806¢E	12/05/2008
MAD03	Madagascar	Nosy Be	Ampasindava	MGNW-15	*Stegastes*	*nigricans*	Inner reef	1	4	13°42000¢S	48°30806¢E	12/05/2008
MAD03	Madagascar	Nosy Be	Ampasindava	MGNW-23	*Cheilio*	*inermis*	Inner reef	1	4	13°41389¢S	48°30556¢E	16/05/2008
MAD03	Madagascar	Nosy Be	Ampasindava	MGNW-23	*Stethojulis*	*strigiventer*	Inner reef	1	4	13°41389¢S	48°30556¢E	16/05/2008
MAD03	Madagascar	Nosy Be	Ampasindava	MGNW-23	*Chrysiptera*	*annulata*	Inner reef	1	4	13°41389¢S	48°30556¢E	16/05/2008
MAD04	Madagascar	Nosy Be	Nosy-Kivindry	MGNW-16	*Anampses*	*meleagrides*	Outer slope	3	10	13°50389¢S	47°95167¢E	13/05/2008
MAD04	Madagascar	Nosy Be	Nosy-Kivindry	MGNW-16	*Bodianus*	*anthioïdes*	Outer slope	3	10	13°50389¢S	47°95167¢E	13/05/2008
MAD04	Madagascar	Nosy Be	Nosy-Kivindry	MGNW-16	*Bodianus*	*diana*	Outer slope	3	10	13°50389¢S	47°95167¢E	13/05/2008
MAD04	Madagascar	Nosy Be	Nosy-Kivindry	MGNW-16	*Bodianus*	*perditio*	Outer slope	3	10	13°50389¢S	47°95167¢E	13/05/2008
MAD04	Madagascar	Nosy Be	Nosy-Kivindry	MGNW-16	*Labroides*	*bicolor*	Outer slope	3	10	13°50389¢S	47°95167¢E	13/05/2008
MAD04	Madagascar	Nosy Be	Nosy-Kivindry	MGNW-16	*Macropharyngodon*	*bipartitus*	Outer slope	3	10	13°50389¢S	47°95167¢E	13/05/2008
MAD04	Madagascar	Nosy Be	Nosy-Kivindry	MGNW-16	*Amphiprion*	*latifasciatus*	Outer slope	3	10	13°50389¢S	47°95167¢E	13/05/2008
MAD04	Madagascar	Nosy Be	Nosy-Kivindry	MGNW-17	*Chaetodon*	*kleinii*	Outer slope	3	10	13°50389¢S	47°95167¢E	13/05/2008
MAD04	Madagascar	Nosy Be	Nosy-Kivindry	MGNW-17	*Chaetodon*	*kleinii*	Outer slope	3	10	13°50389¢S	47°95167¢E	13/05/2008
MAD04	Madagascar	Nosy Be	Nosy-Kivindry	MGNW-17	*Heniochus*	*acuminatus*	Outer slope	3	10	13°50389¢S	47°95167¢E	13/05/2008
MAD04	Madagascar	Nosy Be	Nosy-Kivindry	MGNW-17	*Bodianus*	*diana*	Outer slope	3	10	13°50389¢S	47°95167¢E	13/05/2008
MAD04	Madagascar	Nosy Be	Nosy-Kivindry	MGNW-17	*Labroides*	*dimidiatus*	Outer slope	3	10	13°50389¢S	47°95167¢E	13/05/2008
MAD04	Madagascar	Nosy Be	Nosy-Kivindry	MGNW-17	*Thalassoma*	*amblycephalum*	Outer slope	3	10	13°50389¢S	47°95167¢E	13/05/2008
MAD04	Madagascar	Nosy Be	Nosy-Kivindry	MGNW-17	*Thalassoma*	*amblycephalum*	Outer slope	3	10	13°50389¢S	47°95167¢E	13/05/2008
MAD04	Madagascar	Nosy Be	Nosy-Kivindry	MGNW-17	*Thalassoma*	*amblycephalum*	Outer slope	3	10	13°50389¢S	47°95167¢E	13/05/2008
MAD04	Madagascar	Nosy Be	Nosy-Kivindry	MGNW-17	*Thalassoma*	*lunare*	Outer slope	3	10	13°50389¢S	47°95167¢E	13/05/2008
MAD04	Madagascar	Nosy Be	Nosy-Kivindry	MGNW-17	*Thalassoma*	*lunare*	Outer slope	3	10	13°50389¢S	47°95167¢E	13/05/2008
MAD04	Madagascar	Nosy Be	Nosy-Kivindry	MGNW-17	*Thalassoma*	*lunare*	Outer slope	3	10	13°50389¢S	47°95167¢E	13/05/2008
MAD04	Madagascar	Nosy Be	Nosy-Kivindry	MGNW-17	*Chromis*	*ternatensis*	Outer slope	3	10	13°50389¢S	47°95167¢E	13/05/2008
MAD04	Madagascar	Nosy Be	Nosy-Kivindry	MGNW-17	*Chromis*	*weberi*	Outer slope	3	10	13°50389¢S	47°95167¢E	13/05/2008
MAD04	Madagascar	Nosy Be	Nosy-Kivindry	MGNW-17	*Neopomacentrus*	*cyanomos*	Outer slope	3	10	13°50389¢S	47°95167¢E	13/05/2008
MAD04	Madagascar	Nosy Be	Nosy-Kivindry	MGNW-17	*Pomacentrus*	*caeruleus*	Outer slope	3	10	13°50389¢S	47°95167¢E	13/05/2008
MAD04	Madagascar	Nosy Be	Nosy Hotel	MGNW-20	*Halichoeres*	*nebulosus*	Inner reef	3	6	13°42444¢S	48°36417¢E	15/05/2008
MAD04	Madagascar	Nosy Be	Nosy Hotel	MGNW-20	*Hemigymnus*	*fasciatus*	Inner reef	3	6	13°42444¢S	48°36417¢E	15/05/2008
MAD05	Madagascar	Nosy Be	Nosy Tanikely	MGNW-19	*Chaetodon*	*trifascialis*	Inner reef	0	2	13°48056¢S	48°23333¢E	14/05/2008
MAD05	Madagascar	Nosy Be	Nosy Tanikely	MGNW-19	*Anampses*	*meleagrides*	Inner reef	0	2	13°48056¢S	48°23333¢E	14/05/2008
MAD05	Madagascar	Nosy Be	Nosy Tanikely	MGNW-19	*Anampses*	*twistii*	Inner reef	0	2	13°48056¢S	48°23333¢E	14/05/2008
MAD05	Madagascar	Nosy Be	Nosy Tanikely	MGNW-19	*Coris*	*caudimacula*	Inner reef	0	2	13°48056¢S	48°23333¢E	14/05/2008
MAD05	Madagascar	Nosy Be	Nosy Tanikely	MGNW-19	*Halichoeres*	*scapularis*	Inner reef	0	2	13°48056¢S	48°23333¢E	14/05/2008
MAD05	Madagascar	Nosy Be	Nosy Tanikely	MGNW-19	*Hemigymnus*	*fasciatus*	Inner reef	0	2	13°48056¢S	48°23333¢E	14/05/2008
MAD05	Madagascar	Nosy Be	Nosy Tanikely	MGNW-19	*Hologymnosus*	*annulatus*	Inner reef	0	2	13°48056¢S	48°23333¢E	14/05/2008
MAD05	Madagascar	Nosy Be	Nosy Tanikely	MGNW-19	*Stethojulis*	*bandanensis*	Inner reef	0	2	13°48056¢S	48°23333¢E	14/05/2008
MAD05	Madagascar	Nosy Be	Nosy Tanikely	MGNW-19	*Thalassoma*	*hardwicke*	Inner reef	0	2	13°48056¢S	48°23333¢E	14/05/2008
MAD05	Madagascar	Nosy Be	Nosy Tanikely	MGNW-19	*Thalassoma*	*hardwicke*	Inner reef	0	2	13°48056¢S	48°23333¢E	14/05/2008
MAD05	Madagascar	Nosy Be	Nosy Tanikely	MGNW-19	*Thalassoma*	*hardwicke*	Inner reef	0	2	13°48056¢S	48°23333¢E	14/05/2008
MAD05	Madagascar	Nosy Be	Nosy Tanikely	MGNW-19	*Thalassoma*	*hebraicum*	Inner reef	0	2	13°48056¢S	48°23333¢E	14/05/2008
MAD05	Madagascar	Nosy Be	Nosy Tanikely	MGNW-19	*Thalassoma*	*hebraicum*	Inner reef	0	2	13°48056¢S	48°23333¢E	14/05/2008
MAD05	Madagascar	Nosy Be	Nosy Tanikely	MGNW-19	*Thalassoma*	*hebraicum*	Inner reef	0	2	13°48056¢S	48°23333¢E	14/05/2008
MAD05	Madagascar	Nosy Be	Nosy Tanikely	MGNW-19	*Thalassoma*	*hebraicum*	Inner reef	0	2	13°48056¢S	48°23333¢E	14/05/2008
MAD05	Madagascar	Nosy Be	Nosy Tanikely	MGNW-19	*Thalassoma*	*hebraicum*	Inner reef	0	2	13°48056¢S	48°23333¢E	14/05/2008
MAD05	Madagascar	Nosy Be	Nosy Tanikely	MGNW-19	*Amphiprion*	*latifasciatus*	Inner reef	0	2	13°48056¢S	48°23333¢E	14/05/2008
MAD05	Madagascar	Nosy Be	Nosy Tanikely	MGNW-19	*Dascyllus*	*aruanus*	Inner reef	0	2	13°48056¢S	48°23333¢E	14/05/2008
MAD05	Madagascar	Nosy Be	Nosy Tanikely	MGNW-19	*Plectroglyphidodon*	*lacrymatus*	Inner reef	0	2	13°48056¢S	48°23333¢E	14/05/2008
MAD05	Madagascar	Nosy Be	Nosy Tanikely	MGNW-19	*Pomacentrus*	*trilineatus*	Inner reef	0	2	13°48056¢S	48°23333¢E	14/05/2008
MAD05	Madagascar	Nosy Be	Nosy Tanikely	MGNW-19	*Stegastes*	*nigricans*	Inner reef	0	2	13°48056¢S	48°23333¢E	14/05/2008
MAD06	Madagascar	Nosy Be	Nosy Vorona	MGNW-03	*Chaetodon*	*auriga*	Inner reef	0	2	13°70717¢S	48°60780¢E	08/05/2008
MAD06	Madagascar	Nosy Be	Nosy Vorona	MGNW-03	*Heniochus*	*acuminatus*	Inner reef	0	2	13°70717¢S	48°60780¢E	08/05/2008
MAD06	Madagascar	Nosy Be	Nosy Vorona	MGNW-03	*Cheilinus*	*fasciatus*	Inner reef	0	2	13°70717¢S	48°60780¢E	08/05/2008
MAD06	Madagascar	Nosy Be	Nosy Vorona	MGNW-03	*Hemigymnus*	*fasciatus*	Inner reef	0	2	13°70717¢S	48°60780¢E	08/05/2008
MAD06	Madagascar	Nosy Be	Nosy Vorona	MGNW-03	*Oxycheilinus*	*digramma*	Inner reef	0	2	13°70717¢S	48°60780¢E	08/05/2008
MAD06	Madagascar	Nosy Be	Nosy Vorona	MGNW-03	*Abudefduf*	*sexfasciatus*	Inner reef	0	2	13°70717¢S	48°60780¢E	08/05/2008
MAD06	Madagascar	Nosy Be	Nosy Vorona	MGNW-03	*Abudefduf*	*sparoides*	Inner reef	0	2	13°70717¢S	48°60780¢E	08/05/2008
MAD06	Madagascar	Nosy Be	Nosy Vorona	MGNW-03	*Amphiprion*	*latifasciatus*	Inner reef	0	2	13°70717¢S	48°60780¢E	08/05/2008
MAD06	Madagascar	Nosy Be	Nosy Vorona	MGNW-03	*Chromis*	*xanthochira*	Inner reef	0	2	13°70717¢S	48°60780¢E	08/05/2008
MAD06	Madagascar	Nosy Be	Nosy Vorona	MGNW-03	*Dascyllus*	*aruanus*	Inner reef	0	2	13°70717¢S	48°60780¢E	08/05/2008
MAD06	Madagascar	Nosy Be	Nosy Vorona	MGNW-03	*Dascyllus*	*trimaculatus*	Inner reef	0	2	13°70717¢S	48°60780¢E	08/05/2008
MAD06	Madagascar	Nosy Be	Nosy Vorona	MGNW-03	*Pomacentrus*	*pavo*	Inner reef	0	2	13°70717¢S	48°60780¢E	08/05/2008
MAD06	Madagascar	Nosy Be	Nosy Vorona	MGNW-03	*Chrysiptera*	*unimaculata*	Inner reef	0	2	13°70717¢S	48°60780¢E	08/05/2008
MAD06	Madagascar	Nosy Be	Nosy Vorona	MGNW-04	*Chaetodon*	*auriga*	Inner reef	2	3	13°70717¢S	48°60780¢E	08/05/2008
MAD06	Madagascar	Nosy Be	Nosy Vorona	MGNW-04	*Chaetodon*	*melannotus*	Inner reef	2	3	13°70717¢S	48°60780¢E	08/05/2008
MAD06	Madagascar	Nosy Be	Nosy Vorona	MGNW-04	*Halichoeres*	*dussumieri*	Inner reef	2	3	13°70717¢S	48°60780¢E	08/05/2008
MAD06	Madagascar	Nosy Be	Nosy Vorona	MGNW-04	*Halichoeres*	*nebulosus*	Inner reef	2	3	13°70717¢S	48°60780¢E	08/05/2008
MAD06	Madagascar	Nosy Be	Nosy Vorona	MGNW-04	*Labroides*	*dimidiatus*	Inner reef	2	3	13°70717¢S	48°60780¢E	08/05/2008
MAD06	Madagascar	Nosy Be	Nosy Vorona	MGNW-04	*Oxycheilinus*	*digramma*	Inner reef	2	3	13°70717¢S	48°60780¢E	08/05/2008
MAD06	Madagascar	Nosy Be	Nosy Vorona	MGNW-04	*Thalassoma*	*lunare*	Inner reef	2	3	13°70717¢S	48°60780¢E	08/05/2008
MAD06	Madagascar	Nosy Be	Nosy Vorona	MGNW-04	*Abudefduf*	*vaigiensis*	Inner reef	2	3	13°70717¢S	48°60780¢E	08/05/2008
MAD06	Madagascar	Nosy Be	Nosy Vorona	MGNW-04	*Chrysiptera*	*brownriggii*	Inner reef	2	3	13°70717¢S	48°60780¢E	08/05/2008
MAD06	Madagascar	Nosy Be	Nosy Vorona	MGNW-04	*Neopomacentrus*	*azysron*	Inner reef	2	3	13°70717¢S	48°60780¢E	08/05/2008
MAD06	Madagascar	Nosy Be	Nosy Vorona	MGNW-04	*Neopomacentrus*	*cyanomos*	Inner reef	2	3	13°70717¢S	48°60780¢E	08/05/2008
MAD06	Madagascar	Nosy Be	Nosy Vorona	MGNW-04	*Pomacentrus*	*pavo*	Inner reef	2	3	13°70717¢S	48°60780¢E	08/05/2008
MAD06	Madagascar	Nosy Be	Nosy Vorona	MGNW-04	*Pomacentrus*	*trilineatus*	Inner reef	2	3	13°70717¢S	48°60780¢E	08/05/2008
MAD06	Madagascar	Nosy Be	Nosy Vorona	MGNW-04	*Chrysiptera*	*unimaculata*	Inner reef	2	3	13°70717¢S	48°60780¢E	08/05/2008
MAD07	Madagascar	Nosy Be	Nosy Kivinji	MGNW-05	*Chaetodon*	*guttatissimus*	Outer slope	5	8	13°50389¢S	48°95167¢E	09/05/2008
MAD07	Madagascar	Nosy Be	Nosy Kivinji	MGNW-05	*Chaetodon*	*guttatissimus*	Outer slope	5	8	13°50389¢S	48°95167¢E	09/05/2008
MAD07	Madagascar	Nosy Be	Nosy Kivinji	MGNW-05	*Chaetodon*	*kleinii*	Outer slope	5	8	13°50389¢S	48°95167¢E	09/05/2008
MAD07	Madagascar	Nosy Be	Nosy Kivinji	MGNW-05	*Chaetodon*	*lunula*	Outer slope	5	8	13°50389¢S	48°95167¢E	09/05/2008
MAD07	Madagascar	Nosy Be	Nosy Kivinji	MGNW-05	*Chaetodon*	*melannotus*	Outer slope	5	8	13°50389¢S	48°95167¢E	09/05/2008
MAD07	Madagascar	Nosy Be	Nosy Kivinji	MGNW-05	*Chaetodon*	*meyeri*	Outer slope	5	8	13°50389¢S	48°95167¢E	09/05/2008
MAD07	Madagascar	Nosy Be	Nosy Kivinji	MGNW-05	*Chaetodon*	*vagabundus*	Outer slope	5	8	13°50389¢S	48°95167¢E	09/05/2008
MAD07	Madagascar	Nosy Be	Nosy Kivinji	MGNW-05	*Anampses*	*caeruleopunctatus*	Outer slope	5	8	13°50389¢S	48°95167¢E	09/05/2008
MAD07	Madagascar	Nosy Be	Nosy Kivinji	MGNW-05	*Bodianus*	*axillaris*	Outer slope	5	8	13°50389¢S	48°95167¢E	09/05/2008
MAD07	Madagascar	Nosy Be	Nosy Kivinji	MGNW-05	*Bodianus*	*diana*	Outer slope	5	8	13°50389¢S	48°95167¢E	09/05/2008
MAD07	Madagascar	Nosy Be	Nosy Kivinji	MGNW-05	*Coris*	*aygula*	Outer slope	5	8	13°50389¢S	48°95167¢E	09/05/2008
MAD07	Madagascar	Nosy Be	Nosy Kivinji	MGNW-05	*Coris*	*caudimacula*	Outer slope	5	8	13°50389¢S	48°95167¢E	09/05/2008
MAD07	Madagascar	Nosy Be	Nosy Kivinji	MGNW-05	*Coris*	*cuvieri*	Outer slope	5	8	13°50389¢S	48°95167¢E	09/05/2008
MAD07	Madagascar	Nosy Be	Nosy Kivinji	MGNW-05	*Coris*	*cuvieri*	Outer slope	5	8	13°50389¢S	48°95167¢E	09/05/2008
MAD07	Madagascar	Nosy Be	Nosy Kivinji	MGNW-05	*Coris*	*cuvieri*	Outer slope	5	8	13°50389¢S	48°95167¢E	09/05/2008
MAD07	Madagascar	Nosy Be	Nosy Kivinji	MGNW-05	*Halichoeres*	*cosmetus*	Outer slope	5	8	13°50389¢S	48°95167¢E	09/05/2008
MAD07	Madagascar	Nosy Be	Nosy Kivinji	MGNW-05	*Halichoeres*	*hortulanus*	Outer slope	5	8	13°50389¢S	48°95167¢E	09/05/2008
MAD07	Madagascar	Nosy Be	Nosy Kivinji	MGNW-05	*Hologymnosus*	*annulatus*	Outer slope	5	8	13°50389¢S	48°95167¢E	09/05/2008
MAD07	Madagascar	Nosy Be	Nosy Kivinji	MGNW-05	*Pseudodax*	*moluccanus*	Outer slope	5	8	13°50389¢S	48°95167¢E	09/05/2008
MAD07	Madagascar	Nosy Be	Nosy Kivinji	MGNW-05	*Pomacentrus*	*baenschi*	Outer slope	5	8	13°50389¢S	48°95167¢E	09/05/2008
MAD07	Madagascar	Nosy Be	Nosy Kivinji	MGNW-05	*Pomacentrus*	*caeruleus*	Outer slope	5	8	13°50389¢S	48°95167¢E	09/05/2008
MAD07	Madagascar	Nosy Be	Nosy Kivinji	MGNW-05	*Pomacentrus*	*caeruleus*	Outer slope	5	8	13°50389¢S	48°95167¢E	09/05/2008
MAD07	Madagascar	Nosy Be	Nosy Kivinji	MGNW-05	*Pomacentrus*	*trilineatus*	Outer slope	5	8	13°50389¢S	48°95167¢E	09/05/2008
MAD07	Madagascar	Nosy Be	Nosy Kivinji	MGNW-05	*Pomacentrus*	*trichrous*	Outer slope	5	8	13°50389¢S	48°95167¢E	09/05/2008
MAD07	Madagascar	Nosy Be	Nosy Kivinji	MGNW-05	*Chrysiptera*	*unimaculata*	Outer slope	5	8	13°50389¢S	48°95167¢E	09/05/2008
MAD07	Madagascar	Nosy Be	Nosy Kivinji	MGNW-06	*Pseudocheilinus*	*hexataenia*	Outer slope	5	8	13°50389¢S	48°95167¢E	09/05/2008
MAD07	Madagascar	Nosy Be	Nosy Kivinji	MGNW-06	*Thalassoma*	*amblycephalum*	Outer slope	5	8	13°50389¢S	48°95167¢E	09/05/2008
MAD07	Madagascar	Nosy Be	Nosy Kivinji	MGNW-06	*Chromis*	*caudalis*	Outer slope	5	8	13°50389¢S	48°95167¢E	09/05/2008
MAD07	Madagascar	Nosy Be	Nosy Kivinji	MGNW-06	*Chromis*	*dimidiata*	Outer slope	5	8	13°50389¢S	48°95167¢E	09/05/2008
MAD07	Madagascar	Nosy Be	Nosy Kivinji	MGNW-06	*Chromis*	*iomelas*	Outer slope	5	8	13°50389¢S	48°95167¢E	09/05/2008
MAD07	Madagascar	Nosy Be	Nosy Kivinji	MGNW-06	*Chromis*	*opercularis*	Outer slope	5	8	13°50389¢S	48°95167¢E	09/05/2008
MAD07	Madagascar	Nosy Be	Nosy Kivinji	MGNW-06	*Chromis*	*ternatensis*	Outer slope	5	8	13°50389¢S	48°95167¢E	09/05/2008
MAD07	Madagascar	Nosy Be	Nosy Kivinji	MGNW-06	*Chromis*	*xanthochira*	Outer slope	5	8	13°50389¢S	48°95167¢E	09/05/2008
MAD07	Madagascar	Nosy Be	Nosy Kivinji	MGNW-06	*Pomacentrus*	*caeruleus*	Outer slope	5	8	13°50389¢S	48°95167¢E	09/05/2008
POL01	French Polynesia	Moorea	Club med	MBIO-05	*Chaetodon*	*citrinellus*	Inner reef	0	2	17°50270¢S	149°92500¢W	12/03/2006
POL01	French Polynesia	Moorea	Club med	MBIO-05	*Chaetodon*	*lunula*	Inner reef	0	2	17°50270¢S	149°92500¢W	12/03/2006
POL01	French Polynesia	Moorea	Club med	MBIO-05	*Chaetodon*	*lunulatus*	Inner reef	0	2	17°50270¢S	149°92500¢W	12/03/2006
POL01	French Polynesia	Moorea	Club med	MBIO-05	*Chaetodon*	*pelewensis*	Inner reef	0	2	17°50270¢S	149°92500¢W	12/03/2006
POL01	French Polynesia	Moorea	Club med	MBIO-05	*Chaetodon*	*unimaculatus*	Inner reef	0	2	17°50270¢S	149°92500¢W	12/03/2006
POL01	French Polynesia	Moorea	Club med	MBIO-05	*Chaetodon*	*vagabundus*	Inner reef	0	2	17°50270¢S	149°92500¢W	12/03/2006
POL01	French Polynesia	Moorea	Club med	MBIO-05	*Heniochus*	*chrysostomus*	Inner reef	0	2	17°50270¢S	149°92500¢W	12/03/2006
POL01	French Polynesia	Moorea	Club med	MBIO-05	*Cheilinus*	*trilobatus*	Inner reef	0	2	17°50270¢S	149°92500¢W	12/03/2006
POL01	French Polynesia	Moorea	Club med	MBIO-05	*Coris*	*aygula*	Inner reef	0	2	17°50270¢S	149°92500¢W	12/03/2006
POL01	French Polynesia	Moorea	Club med	MBIO-05	*Epibulus*	*insidiator*	Inner reef	0	2	17°50270¢S	149°92500¢W	12/03/2006
POL01	French Polynesia	Moorea	Club med	MBIO-05	*Gomphosus*	*varius*	Inner reef	0	2	17°50270¢S	149°92500¢W	12/03/2006
POL01	French Polynesia	Moorea	Club med	MBIO-05	*Halichoeres*	*hortulanus*	Inner reef	0	2	17°50270¢S	149°92500¢W	12/03/2006
POL01	French Polynesia	Moorea	Club med	MBIO-05	*Halichoeres*	*margaritaceus*	Inner reef	0	2	17°50270¢S	149°92500¢W	12/03/2006
POL01	French Polynesia	Moorea	Club med	MBIO-05	*Halichoeres*	*trimaculatus*	Inner reef	0	2	17°50270¢S	149°92500¢W	12/03/2006
POL01	French Polynesia	Moorea	Club med	MBIO-05	*Labroides*	*bicolor*	Inner reef	0	2	17°50270¢S	149°92500¢W	12/03/2006
POL01	French Polynesia	Moorea	Club med	MBIO-05	*Labroides*	*dimidiatus*	Inner reef	0	2	17°50270¢S	149°92500¢W	12/03/2006
POL01	French Polynesia	Moorea	Club med	MBIO-05	*Pseudocheilinus*	*evanidus*	Inner reef	0	2	17°50270¢S	149°92500¢W	12/03/2006
POL01	French Polynesia	Moorea	Club med	MBIO-05	*Stethojulis*	*bandanensis*	Inner reef	0	2	17°50270¢S	149°92500¢W	12/03/2006
POL01	French Polynesia	Moorea	Club med	MBIO-05	*Thalassoma*	*hardwicke*	Inner reef	0	2	17°50270¢S	149°92500¢W	12/03/2006
POL01	French Polynesia	Moorea	Club med	MBIO-05	*Abudefduf*	*septemfasciatus*	Inner reef	0	2	17°50270¢S	149°92500¢W	12/03/2006
POL01	French Polynesia	Moorea	Club med	MBIO-05	*Chromis*	*viridis*	Inner reef	0	2	17°50270¢S	149°92500¢W	12/03/2006
POL01	French Polynesia	Moorea	Club med	MBIO-05	*Chrysiptera*	*brownriggii*	Inner reef	0	2	17°50270¢S	149°92500¢W	12/03/2006
POL01	French Polynesia	Moorea	Club med	MBIO-05	*Dascyllus*	*aruanus*	Inner reef	0	2	17°50270¢S	149°92500¢W	12/03/2006
POL01	French Polynesia	Moorea	Club med	MBIO-05	*Dascyllus*	*flavicaudus*	Inner reef	0	2	17°50270¢S	149°92500¢W	12/03/2006
POL01	French Polynesia	Moorea	Club med	MBIO-05	*Pomacentrus*	*pavo*	Inner reef	0	2	17°50270¢S	149°92500¢W	12/03/2006
POL01	French Polynesia	Moorea	Club med	MBIO-05	*Stegastes*	*albifasciatus*	Inner reef	0	2	17°50270¢S	149°92500¢W	12/03/2006
POL01	French Polynesia	Moorea	Paroa	MBIO-07	*Chaetodon*	*auriga*	Inner reef	0	2	17°60630¢S	149°83400¢W	15/03/2006
POL01	French Polynesia	Moorea	Paroa	MBIO-07	*Chaetodon*	*citrinellus*	Inner reef	0	2	17°60630¢S	149°83400¢W	15/03/2006
POL01	French Polynesia	Moorea	Paroa	MBIO-07	*Chaetodon*	*reticulatus*	Inner reef	0	2	17°60630¢S	149°83400¢W	15/03/2006
POL01	French Polynesia	Moorea	Paroa	MBIO-07	*Chaetodon*	*unimaculatus*	Inner reef	0	2	17°60630¢S	149°83400¢W	15/03/2006
POL01	French Polynesia	Moorea	Paroa	MBIO-07	*Heniochus*	*chrysostomus*	Inner reef	0	2	17°60630¢S	149°83400¢W	15/03/2006
POL01	French Polynesia	Moorea	Paroa	MBIO-07	*Coris*	*aygula*	Inner reef	0	2	17°60630¢S	149°83400¢W	15/03/2006
POL01	French Polynesia	Moorea	Paroa	MBIO-07	*Gomphosus*	*varius*	Inner reef	0	2	17°60630¢S	149°83400¢W	15/03/2006
POL01	French Polynesia	Moorea	Paroa	MBIO-07	*Halichoeres*	*hortulanus*	Inner reef	0	2	17°60630¢S	149°83400¢W	15/03/2006
POL01	French Polynesia	Moorea	Paroa	MBIO-07	*Halichoeres*	*trimaculatus*	Inner reef	0	2	17°60630¢S	149°83400¢W	15/03/2006
POL01	French Polynesia	Moorea	Paroa	MBIO-07	*Pseudocheilinus*	*hexataenia*	Inner reef	0	2	17°60630¢S	149°83400¢W	15/03/2006
POL01	French Polynesia	Moorea	Paroa	MBIO-07	*Stethojulis*	*bandanensis*	Inner reef	0	2	17°60630¢S	149°83400¢W	15/03/2006
POL01	French Polynesia	Moorea	Paroa	MBIO-07	*Thalassoma*	*hardwicke*	Inner reef	0	2	17°60630¢S	149°83400¢W	15/03/2006
POL01	French Polynesia	Moorea	Paroa	MBIO-07	*Thalassoma*	*lutescens*	Inner reef	0	2	17°60630¢S	149°83400¢W	15/03/2006
POL01	French Polynesia	Moorea	Paroa	MBIO-07	*Thalassoma*	*trilobatum*	Inner reef	0	2	17°60630¢S	149°83400¢W	15/03/2006
POL01	French Polynesia	Moorea	Paroa	MBIO-07	*Abudefduf*	*sexfasciatus*	Inner reef	0	2	17°60630¢S	149°83400¢W	15/03/2006
POL01	French Polynesia	Moorea	Paroa	MBIO-07	*Chromis*	*atripectoralis*	Inner reef	0	2	17°60630¢S	149°83400¢W	15/03/2006
POL01	French Polynesia	Moorea	Paroa	MBIO-07	*Chromis*	*viridis*	Inner reef	0	2	17°60630¢S	149°83400¢W	15/03/2006
POL01	French Polynesia	Moorea	Paroa	MBIO-07	*Chrysiptera*	*brownriggii*	Inner reef	0	2	17°60630¢S	149°83400¢W	15/03/2006
POL01	French Polynesia	Moorea	Paroa	MBIO-07	*Dascyllus*	*aruanus*	Inner reef	0	2	17°60630¢S	149°83400¢W	15/03/2006
POL01	French Polynesia	Moorea	Paroa	MBIO-07	*Dascyllus*	*flavicaudus*	Inner reef	0	2	17°60630¢S	149°83400¢W	15/03/2006
POL01	French Polynesia	Moorea	Paroa	MBIO-07	*Plectroglyphidodon*	*leucozonus*	Inner reef	0	2	17°60630¢S	149°83400¢W	15/03/2006
POL01	French Polynesia	Moorea	Paroa	MBIO-07	*Stegastes*	*albifasciatus*	Inner reef	0	2	17°60630¢S	149°83400¢W	15/03/2006
POL01	French Polynesia	Moorea	Haapiti	MBIO-08	*Chaetodon*	*lunula*	Inner reef	0	2	17°60630¢S	149°83400¢W	15/03/2006
POL01	French Polynesia	Moorea	Haapiti	MBIO-08	*Chaetodon*	*vagabundus*	Inner reef	0	2	17°60630¢S	149°83400¢W	15/03/2006
POL01	French Polynesia	Moorea	Haapiti	MBIO-08	*Abudefduf*	*septemfasciatus*	Inner reef	0	2	17°60630¢S	149°83400¢W	15/03/2006
POL01	French Polynesia	Moorea	Opunohu Bay	MBIO-14	*Heniochus*	*acuminatus*	Inner reef	0	3	17°51330¢S	149°84960¢W	19/03/2006
POL01	French Polynesia	Moorea	Opunohu Bay	MBIO-14	*Heniochus*	*monoceros*	Inner reef	0	3	17°51330¢S	149°84960¢W	19/03/2006
POL01	French Polynesia	Moorea	Opunohu Bay	MBIO-14	*Cheilinus*	*chlorourus*	Inner reef	0	3	17°51330¢S	149°84960¢W	19/03/2006
POL01	French Polynesia	Moorea	Opunohu Bay	MBIO-14	*Stethojulis*	*bandanensis*	Inner reef	0	3	17°51330¢S	149°84960¢W	19/03/2006
POL01	French Polynesia	Moorea	Beach comber	MBIO-20	*Chaetodon*	*ulietensis*	Inner reef	1	2	17°48550¢S	149°88910¢W	22/03/2006
POL01	French Polynesia	Moorea	Beach comber	MBIO-20	*Bodianus*	*axillaris*	Inner reef	1	2	17°48550¢S	149°88910¢W	22/03/2006
POL01	French Polynesia	Moorea	Beach comber	MBIO-20	*Halichoeres*	*marginatus*	Inner reef	1	2	17°48550¢S	149°88910¢W	22/03/2006
POL01	French Polynesia	Moorea	Haapiti Pass	MBIO-32	*Dascyllus*	*aruanus*	Inner reef	1	3	17°48530¢S	149°85860¢W	27/03/2006
POL01	French Polynesia	Moorea	Beachcomber hotel	MBIO-37	*Epibulus*	*insidiator*	Inner reef	1	8	17°49220¢S	149°92530¢W	28/03/2006
POL01	French Polynesia	Moorea	Beachcomber hotel	MBIO-37	*Labroides*	*bicolor*	Inner reef	1	8	17°49220¢S	149°92530¢W	28/03/2006
POL01	French Polynesia	Moorea	Beachcomber hotel	MBIO-37	*Wetmorella*	*nigropinnata*	Inner reef	1	8	17°49220¢S	149°92530¢W	28/03/2006
POL01	French Polynesia	Moorea	Beachcomber hotel	MBIO-37	*Pomacentrus*	*pavo*	Inner reef	1	8	17°49220¢S	149°92530¢W	28/03/2006
POL01	French Polynesia	Moorea	Temae	MBIO-39	*Chrysiptera*	*brownriggii*	Inner reef	1	3	17°49220¢S	149°92530¢W	28/03/2006
POL01	French Polynesia	Moorea	Temae	MBIO-39	*Chrysiptera*	*unimaculata*	Inner reef	1	3	17°49220¢S	149°92530¢W	28/03/2006
POL02	French Polynesia	Moorea	Matautia	MBIO-01	*Chaetodon*	*pelewensis*	Outer slope	12	18	17°48240¢S	149°88300¢W	10/03/2006
POL02	French Polynesia	Moorea	Matautia	MBIO-01	*Chaetodon*	*reticulatus*	Outer slope	12	18	17°48240¢S	149°88300¢W	10/03/2006
POL02	French Polynesia	Moorea	Matautia	MBIO-01	*Cirrhilabrus*	*scottorum*	Outer slope	12	18	17°48240¢S	149°88300¢W	10/03/2006
POL02	French Polynesia	Moorea	Matautia	MBIO-01	*Halichoeres*	*hortulanus*	Outer slope	12	18	17°48240¢S	149°88300¢W	10/03/2006
POL02	French Polynesia	Moorea	Matautia	MBIO-01	*Halichoeres*	*ornatissimus*	Outer slope	12	18	17°48240¢S	149°88300¢W	10/03/2006
POL02	French Polynesia	Moorea	Matautia	MBIO-01	*Pseudocheilinus*	*hexataenia*	Outer slope	12	18	17°48240¢S	149°88300¢W	10/03/2006
POL02	French Polynesia	Moorea	Matautia	MBIO-01	*Pseudocheilinus*	*octotaenia*	Outer slope	12	18	17°48240¢S	149°88300¢W	10/03/2006
POL02	French Polynesia	Moorea	Matautia	MBIO-01	*Pseudocheilinus*	*tetrataenia*	Outer slope	12	18	17°48240¢S	149°88300¢W	10/03/2006
POL02	French Polynesia	Moorea	Matautia	MBIO-01	*Wetmorella*	*nigropinnata*	Outer slope	12	18	17°48240¢S	149°88300¢W	10/03/2006
POL02	French Polynesia	Moorea	Matautia	MBIO-01	*Chromis*	*acares*	Outer slope	12	18	17°48240¢S	149°88300¢W	10/03/2006
POL02	French Polynesia	Moorea	Matautia	MBIO-01	*Chromis*	*iomelas*	Outer slope	12	18	17°48240¢S	149°88300¢W	10/03/2006
POL02	French Polynesia	Moorea	Matautia	MBIO-01	*Dascyllus*	*flavicaudus*	Outer slope	12	18	17°48240¢S	149°88300¢W	10/03/2006
POL02	French Polynesia	Moorea	Matautia	MBIO-01	*Plectroglyphidodon*	*johnstonianus*	Outer slope	12	18	17°48240¢S	149°88300¢W	10/03/2006
POL02	French Polynesia	Moorea	Matautia	MBIO-01	*Plectroglyphidodon*	*lacrymatus*	Outer slope	12	18	17°48240¢S	149°88300¢W	10/03/2006
POL02	French Polynesia	Moorea	Matautia	MBIO-04	*Chaetodon*	*ornatissimus*	Outer slope	2	4	17°48250¢S	149°88210¢W	11/03/2006
POL02	French Polynesia	Moorea	Matautia	MBIO-04	*Forcipiger*	*flavissimus*	Outer slope	2	4	17°48250¢S	149°88210¢W	11/03/2006
POL02	French Polynesia	Moorea	Matautia	MBIO-04	*Cirrhilabrus*	*scottorum*	Outer slope	2	4	17°48250¢S	149°88210¢W	11/03/2006
POL02	French Polynesia	Moorea	Matautia	MBIO-04	*Thalassoma*	*amblycephalum*	Outer slope	2	4	17°48250¢S	149°88210¢W	11/03/2006
POL02	French Polynesia	Moorea	Matautia	MBIO-04	*Thalassoma*	*quinquevittatum*	Outer slope	2	4	17°48250¢S	149°88210¢W	11/03/2006
POL02	French Polynesia	Moorea	Matautia	MBIO-04	*Thalassoma*	*trilobatum*	Outer slope	2	4	17°48250¢S	149°88210¢W	11/03/2006
POL02	French Polynesia	Moorea	Matautia	MBIO-04	*Chromis*	*vanderbilti*	Outer slope	2	4	17°48250¢S	149°88210¢W	11/03/2006
POL02	French Polynesia	Moorea	Matautia	MBIO-04	*Plectroglyphidodon*	*imparipennis*	Outer slope	2	4	17°48250¢S	149°88210¢W	11/03/2006
POL02	French Polynesia	Moorea	Matautia	MBIO-04	*Plectroglyphidodon*	*phoenixensis*	Outer slope	2	4	17°48250¢S	149°88210¢W	11/03/2006
POL02	French Polynesia	Moorea	Matautia	MBIO-04	*Chrysiptera*	*unimaculata*	Outer slope	2	4	17°48250¢S	149°88210¢W	11/03/2006
POL02	French Polynesia	Moorea	Opunohu	MBIO-06	*Chaetodon*	*mertensii*	Outer slope	3	34	17°49460¢S	149°86190¢W	14/03/2006
POL02	French Polynesia	Moorea	Opunohu	MBIO-06	*Chaetodon*	*trichrous*	Outer slope	3	34	17°49460¢S	149°86190¢W	14/03/2006
POL02	French Polynesia	Moorea	Opunohu	MBIO-06	*Forcipiger*	*flavissimus*	Outer slope	3	34	17°49460¢S	149°86190¢W	14/03/2006
POL02	French Polynesia	Moorea	Opunohu	MBIO-06	*Heniochus*	*acuminatus*	Outer slope	3	34	17°49460¢S	149°86190¢W	14/03/2006
POL02	French Polynesia	Moorea	Opunohu	MBIO-06	*Coris*	*aygula*	Outer slope	3	34	17°49460¢S	149°86190¢W	14/03/2006
POL02	French Polynesia	Moorea	Opunohu	MBIO-06	*Coris*	*gaimard*	Outer slope	3	34	17°49460¢S	149°86190¢W	14/03/2006
POL02	French Polynesia	Moorea	Opunohu	MBIO-06	*Epibulus*	*insidiator*	Outer slope	3	34	17°49460¢S	149°86190¢W	14/03/2006
POL02	French Polynesia	Moorea	Opunohu	MBIO-06	*Pseudocheilinus*	*evanidus*	Outer slope	3	34	17°49460¢S	149°86190¢W	14/03/2006
POL02	French Polynesia	Moorea	Opunohu	MBIO-06	*Thalassoma*	*quinquevittatum*	Outer slope	3	34	17°49460¢S	149°86190¢W	14/03/2006
POL02	French Polynesia	Moorea	Opunohu	MBIO-06	*Chromis*	*iomelas*	Outer slope	3	34	17°49460¢S	149°86190¢W	14/03/2006
POL02	French Polynesia	Moorea	Opunohu pass	MBIO-10	*Chaetodon*	*trichrous*	Outer slope	1	15	17°48900¢S	149°85800¢W	16/03/2006
POL02	French Polynesia	Moorea	Opunohu pass	MBIO-10	*Chaetodon*	*vagabundus*	Outer slope	1	15	17°48900¢S	149°85800¢W	16/03/2006
POL02	French Polynesia	Moorea	Papetoai	MBIO-11	*Chaetodon*	*quadrimaculatus*	Outer slope	5	35	17°48430¢S	149°86940¢W	17/03/2006
POL02	French Polynesia	Moorea	Papetoai	MBIO-11	*Chaetodon*	*trifascialis*	Outer slope	5	35	17°48430¢S	149°86940¢W	17/03/2006
POL02	French Polynesia	Moorea	Papetoai	MBIO-11	*Forcipiger*	*longirostris*	Outer slope	5	35	17°48430¢S	149°86940¢W	17/03/2006
POL02	French Polynesia	Moorea	Papetoai	MBIO-11	*Hemitaurichthys*	*polylepis*	Outer slope	5	35	17°48430¢S	149°86940¢W	17/03/2006
POL02	French Polynesia	Moorea	Papetoai	MBIO-11	*Hemigymnus*	*fasciatus*	Outer slope	5	35	17°48430¢S	149°86940¢W	17/03/2006
POL02	French Polynesia	Moorea	Papetoai	MBIO-11	*Chromis*	*alpha*	Outer slope	5	35	17°48430¢S	149°86940¢W	17/03/2006
POL02	French Polynesia	Moorea	Papetoai	MBIO-11	*Chromis*	*xanthura*	Outer slope	5	35	17°48430¢S	149°86940¢W	17/03/2006
POL02	French Polynesia	Moorea	Tiahura	MBIO-12	*Chaetodon*	*ornatissimus*	Outer slope	25	35	17°48260¢S	149°89990¢W	18/03/2006
POL02	French Polynesia	Moorea	Tiahura	MBIO-12	*Chaetodon*	*pelewensis*	Outer slope	25	35	17°48260¢S	149°89990¢W	18/03/2006
POL02	French Polynesia	Moorea	Tiahura	MBIO-12	*Chaetodon*	*reticulatus*	Outer slope	25	35	17°48260¢S	149°89990¢W	18/03/2006
POL02	French Polynesia	Moorea	Tiahura	MBIO-12	*Chaetodon*	*unimaculatus*	Outer slope	25	35	17°48260¢S	149°89990¢W	18/03/2006
POL02	French Polynesia	Moorea	Tiahura	MBIO-12	*Forcipiger*	*flavissimus*	Outer slope	25	35	17°48260¢S	149°89990¢W	18/03/2006
POL02	French Polynesia	Moorea	Tiahura	MBIO-12	*Cheilinus*	*oxycephalus*	Outer slope	25	35	17°48260¢S	149°89990¢W	18/03/2006
POL02	French Polynesia	Moorea	Tiahura	MBIO-12	*Cirrhilabrus*	*exquisitus*	Outer slope	25	35	17°48260¢S	149°89990¢W	18/03/2006
POL02	French Polynesia	Moorea	Tiahura	MBIO-12	*Cirrhilabrus*	*scottorum*	Outer slope	25	35	17°48260¢S	149°89990¢W	18/03/2006
POL02	French Polynesia	Moorea	Tiahura	MBIO-12	*Labroides*	*dimidiatus*	Outer slope	25	35	17°48260¢S	149°89990¢W	18/03/2006
POL02	French Polynesia	Moorea	Tiahura	MBIO-12	*Pseudocheilinus*	*evanidus*	Outer slope	25	35	17°48260¢S	149°89990¢W	18/03/2006
POL02	French Polynesia	Moorea	Tiahura	MBIO-12	*Pseudocheilinus*	*hexataenia*	Outer slope	25	35	17°48260¢S	149°89990¢W	18/03/2006
POL02	French Polynesia	Moorea	Tiahura	MBIO-12	*Pseudocheilinus*	*ocellatus*	Outer slope	25	35	17°48260¢S	149°89990¢W	18/03/2006
POL02	French Polynesia	Moorea	Tiahura	MBIO-12	*Pseudocheilinus*	*octotaenia*	Outer slope	25	35	17°48260¢S	149°89990¢W	18/03/2006
POL02	French Polynesia	Moorea	Tiahura	MBIO-12	*Thalassoma*	*amblycephalum*	Outer slope	25	35	17°48260¢S	149°89990¢W	18/03/2006
POL02	French Polynesia	Moorea	Tiahura	MBIO-12	*Thalassoma*	*quinquevittatum*	Outer slope	25	35	17°48260¢S	149°89990¢W	18/03/2006
POL02	French Polynesia	Moorea	Tiahura	MBIO-12	*Chromis*	*acares*	Outer slope	25	35	17°48260¢S	149°89990¢W	18/03/2006
POL02	French Polynesia	Moorea	Tiahura	MBIO-12	*Chromis*	*alpha*	Outer slope	25	35	17°48260¢S	149°89990¢W	18/03/2006
POL02	French Polynesia	Moorea	Tiahura	MBIO-12	*Chromis*	*iomelas*	Outer slope	25	35	17°48260¢S	149°89990¢W	18/03/2006
POL02	French Polynesia	Moorea	Tiahura	MBIO-12	*Chromis*	*vanderbilti*	Outer slope	25	35	17°48260¢S	149°89990¢W	18/03/2006
POL02	French Polynesia	Moorea	Tiahura	MBIO-12	*Dascyllus*	*flavicaudus*	Outer slope	25	35	17°48260¢S	149°89990¢W	18/03/2006
POL02	French Polynesia	Moorea	Tiahura	MBIO-12	*Dascyllus*	*trimaculatus*	Outer slope	25	35	17°48260¢S	149°89990¢W	18/03/2006
POL02	French Polynesia	Moorea	Tiahura	MBIO-12	*Plectroglyphidodon*	*johnstonianus*	Outer slope	25	35	17°48260¢S	149°89990¢W	18/03/2006
POL02	French Polynesia	Moorea	Tiahura	MBIO-12	*Pomachromis*	*fuscidorsalis*	Outer slope	25	35	17°48260¢S	149°89990¢W	18/03/2006
POL02	French Polynesia	Moorea	Opunoho West	MBIO-13	*Chaetodon*	*lunula*	Outer slope	3	30	17°50580¢S	149°85710¢W	19/03/2006
POL02	French Polynesia	Moorea	Taotai Pass	MBIO-15	*Forcipiger*	*longirostris*	Outer slope	3	45	17°48230¢S	149°89330¢W	20/03/2006
POL02	French Polynesia	Moorea	Taotai Pass	MBIO-15	*Labropsis*	*polynesica*	Outer slope	3	45	17°48230¢S	149°89330¢W	20/03/2006
POL02	French Polynesia	Moorea	Taotai Pass	MBIO-15	*Amphiprion*	*chrysopterus*	Outer slope	3	45	17°48230¢S	149°89330¢W	20/03/2006
POL02	French Polynesia	Moorea	Taotai Pass	MBIO-15	*Chromis*	*agilis*	Outer slope	3	45	17°48230¢S	149°89330¢W	20/03/2006
POL02	French Polynesia	Moorea	Taotai Pass	MBIO-15	*Chrysiptera*	*unimaculata*	Outer slope	3	45	17°48230¢S	149°89330¢W	20/03/2006
POL02	French Polynesia	Moorea	Taotai Pass	MBIO-15	*Stegastes*	*nigricans*	Outer slope	3	45	17°48230¢S	149°89330¢W	20/03/2006
POL02	French Polynesia	Moorea	Entre deux baies	MBIO-17	*Chaetodon*	*ephippium*	Outer slope	5	35	17°47530¢S	149°84080¢W	21/03/2006
POL02	French Polynesia	Moorea	Entre deux baies	MBIO-17	*Chaetodon*	*trifascialis*	Outer slope	5	35	17°47530¢S	149°84080¢W	21/03/2006
POL02	French Polynesia	Moorea	Entre deux baies	MBIO-17	*Heniochus*	*monoceros*	Outer slope	5	35	17°47530¢S	149°84080¢W	21/03/2006
POL02	French Polynesia	Moorea	Entre deux baies	MBIO-17	*Anampses*	*twistii*	Outer slope	5	35	17°47530¢S	149°84080¢W	21/03/2006
POL02	French Polynesia	Moorea	Entre deux baies	MBIO-17	*Gomphosus*	*varius*	Outer slope	5	35	17°47530¢S	149°84080¢W	21/03/2006
POL02	French Polynesia	Moorea	Entre deux baies	MBIO-17	*Hemigymnus*	*fasciatus*	Outer slope	5	35	17°47530¢S	149°84080¢W	21/03/2006
POL02	French Polynesia	Moorea	Entre deux baies	MBIO-17	*Oxycheilinus*	*unifasciatus*	Outer slope	5	35	17°47530¢S	149°84080¢W	21/03/2006
POL02	French Polynesia	Moorea	Entre deux baies	MBIO-17	*Chromis*	*xanthura*	Outer slope	5	35	17°47530¢S	149°84080¢W	21/03/2006
POL02	French Polynesia	Moorea	Entre deux baies	MBIO-17	*Dascyllus*	*trimaculatus*	Outer slope	5	35	17°47530¢S	149°84080¢W	21/03/2006
POL02	French Polynesia	Moorea	Entre deux baies	MBIO-17	*Plectroglyphidodon*	*lacrymatus*	Outer slope	5	35	17°47530¢S	149°84080¢W	21/03/2006
POL02	French Polynesia	Moorea	Club med	MBIO-19	*Chaetodon*	*ulietensis*	Outer slope	5	35	17°48440¢S	149°91580¢W	22/03/2006
POL02	French Polynesia	Moorea	Club med	MBIO-19	*Halichoeres*	*ornatissimus*	Outer slope	5	35	17°48440¢S	149°91580¢W	22/03/2006
POL02	French Polynesia	Moorea	Club med	MBIO-19	*Novaculichthys*	*taeniourus*	Outer slope	5	35	17°48440¢S	149°91580¢W	22/03/2006
POL02	French Polynesia	Moorea	Club med	MBIO-19	*Pseudojuloides*	*atavai*	Outer slope	5	35	17°48440¢S	149°91580¢W	22/03/2006
POL02	French Polynesia	Moorea	Motu Temae	MBIO-23	*Chaetodon*	*bennetti*	Outer slope	20	25	17°47140¢S	149°77280¢W	23/03/2006
POL02	French Polynesia	Moorea	Motu Temae	MBIO-23	*Oxycheilinus*	*unifasciatus*	Outer slope	20	25	17°47140¢S	149°77280¢W	23/03/2006
POL02	French Polynesia	Moorea	Motu Temae	MBIO-23	*Wetmorella*	*nigropinnata*	Outer slope	20	25	17°47140¢S	149°77280¢W	23/03/2006
POL02	French Polynesia	Moorea	Motu Temae	MBIO-24	*Hemitaurichthys*	*polylepis*	Outer slope	13	17	17°47940¢S	149°76390¢W	23/03/2006
POL02	French Polynesia	Moorea	Motu Temae	MBIO-24	*Hemitaurichthys*	*thompsoni*	Outer slope	13	17	17°47940¢S	149°76390¢W	23/03/2006
POL02	French Polynesia	Moorea	Motu Temae	MBIO-24	*Cirrhilabrus*	*scottorum*	Outer slope	13	17	17°47940¢S	149°76390¢W	23/03/2006
POL02	French Polynesia	Moorea	Motu Temae	MBIO-24	*Halichoeres*	*ornatissimus*	Outer slope	13	17	17°47940¢S	149°76390¢W	23/03/2006
POL02	French Polynesia	Moorea	Motu Temae	MBIO-24	*Hemigymnus*	*fasciatus*	Outer slope	13	17	17°47940¢S	149°76390¢W	23/03/2006
POL02	French Polynesia	Moorea	Motu Temae	MBIO-24	*Pseudocheilinus*	*octotaenia*	Outer slope	13	17	17°47940¢S	149°76390¢W	23/03/2006
POL02	French Polynesia	Moorea	Motu Temae	MBIO-24	*Wetmorella*	*nigropinnata*	Outer slope	13	17	17°47940¢S	149°76390¢W	23/03/2006
POL02	French Polynesia	Moorea	Papetoai	MBIO-26	*Pseudocheilinus*	*octotaenia*	Outer slope	15	18	17°47530¢S	149°84080¢W	24/03/2006
POL02	French Polynesia	Moorea	Papetoai	MBIO-26	*Pseudocheilinus*	*tetrataenia*	Outer slope	15	18	17°47530¢S	149°84080¢W	24/03/2006
POL02	French Polynesia	Moorea	Opunohu	MBIO-27	*Cheilio*	*inermis*	Outer slope	2	15	17°47530¢S	149°84080¢W	24/03/2006
POL02	French Polynesia	Moorea	Opunohu	MBIO-27	*Novaculichthys*	*taeniourus*	Outer slope	2	15	17°47530¢S	149°84080¢W	24/03/2006
POL02	French Polynesia	Moorea	Papetoai	MBIO-29	*Forcipiger*	*longirostris*	Outer slope	40	50	17°47530¢S	149°84080¢W	25/03/2006
POL02	French Polynesia	Moorea	Haapiti Pass	MBIO-30	*Chaetodon*	*lunulatus*	Outer slope	2	10	17°47530¢S	149°84080¢W	26/03/2006
POL02	French Polynesia	Moorea	Haapiti Pass	MBIO-30	*Anampses*	*twistii*	Outer slope	2	10	17°47530¢S	149°84080¢W	26/03/2006
POL02	French Polynesia	Moorea	Haapiti Pass	MBIO-30	*Cheilinus*	*chlorourus*	Outer slope	2	10	17°47530¢S	149°84080¢W	26/03/2006
POL02	French Polynesia	Moorea	Haapiti Pass	MBIO-30	*Oxycheilinus*	*unifasciatus*	Outer slope	2	10	17°47530¢S	149°84080¢W	26/03/2006
POL02	French Polynesia	Moorea	Haapiti Pass	MBIO-30	*Thalassoma*	*quinquevittatum*	Outer slope	2	10	17°47530¢S	149°84080¢W	26/03/2006
POL02	French Polynesia	Moorea	Haapiti Pass	MBIO-30	*Wetmorella*	*nigropinnata*	Outer slope	2	10	17°47530¢S	149°84080¢W	26/03/2006
POL02	French Polynesia	Moorea	Haapiti Pass	MBIO-30	*Dascyllus*	*trimaculatus*	Outer slope	2	10	17°47530¢S	149°84080¢W	26/03/2006
POL02	French Polynesia	Moorea	Haapiti Pass	MBIO-30	*Stegastes*	*albifasciatus*	Outer slope	2	10	17°47530¢S	149°84080¢W	26/03/2006
POL02	French Polynesia	Moorea	Haapiti Pass	MBIO-31	*Anampses*	*twistii*	Outer slope	20	30	17°47530¢S	149°84080¢W	26/03/2006
POL02	French Polynesia	Moorea	Haapiti Pass	MBIO-31	*Halichoeres*	*ornatissimus*	Outer slope	20	30	17°47530¢S	149°84080¢W	26/03/2006
POL02	French Polynesia	Moorea	Haapiti Pass	MBIO-31	*Labroides*	*dimidiatus*	Outer slope	20	30	17°47530¢S	149°84080¢W	26/03/2006
POL02	French Polynesia	Moorea	Haapiti Pass	MBIO-31	*Pseudojuloides*	*atavai*	Outer slope	20	30	17°47530¢S	149°84080¢W	26/03/2006
POL02	French Polynesia	Moorea	Haapiti Pass	MBIO-31	*Stethojulis*	*bandanensis*	Outer slope	20	30	17°47530¢S	149°84080¢W	26/03/2006
POL02	French Polynesia	Moorea	Haapiti Pass	MBIO-31	*Chromis*	*acares*	Outer slope	20	30	17°47530¢S	149°84080¢W	26/03/2006
POL02	French Polynesia	Moorea	Haapiti Pass	MBIO-31	*Chromis*	*agilis*	Outer slope	20	30	17°47530¢S	149°84080¢W	26/03/2006
POL02	French Polynesia	Moorea	Opunohu Pass	MBIO-33	*Oxycheilinus*	*arenatus*	Outer slope	3	69	17°48530¢S	149°85860¢W	27/03/2006
POL02	French Polynesia	Moorea	Club Med	MBIO-35	*Pseudocoris*	*aurantiofasciata*	Outer slope	30	35	17°49220¢S	149°92530¢W	28/03/2006
POL02	French Polynesia	Moorea	Club Med	MBIO-36	*Abudefduf*	*sordidus*	Outer slope	1	3	17°49220¢S	149°92530¢W	28/03/2006
POL02	French Polynesia	Moorea	Club Med	MBIO-36	*Plectroglyphidodon*	*imparipennis*	Outer slope	1	3	17°49220¢S	149°92530¢W	28/03/2006
POL02	French Polynesia	Moorea	Club Med	MBIO-36	*Plectroglyphidodon*	*leucozonus*	Outer slope	1	3	17°49220¢S	149°92530¢W	28/03/2006
POL02	French Polynesia	Moorea	Club Med	MBIO-36	*Plectroglyphidodon*	*phoenixensis*	Outer slope	1	3	17°49220¢S	149°92530¢W	28/03/2006
POL02	French Polynesia	Moorea	Club Med	MBIO-36	*Chrysiptera*	*unimaculata*	Outer slope	1	3	17°49220¢S	149°92530¢W	28/03/2006
POL02	French Polynesia	Moorea	Vaiare Pass	MBIO-41	*Labropsis*	*polynesica*	Outer slope	25	35	17°49220¢S	149°92530¢W	30/03/2006
POL02	French Polynesia	Moorea	Vaiare	MBIO-42	*Labroides*	*bicolor*	Outer slope	2	10	17°49220¢S	149°92530¢W	30/03/2006
POL02	French Polynesia	Moorea	Vaiare	MBIO-42	*Amphiprion*	*chrysopterus*	Outer slope	2	10	17°49220¢S	149°92530¢W	30/03/2006
POL02	French Polynesia	Moorea	Vaiare	MBIO-42	*Pomacentrus*	*pavo*	Outer slope	2	10	17°49220¢S	149°92530¢W	30/03/2006
POL02	French Polynesia	Moorea	Papetoai	MBIO-45	*Chaetodon*	*mertensii*	Outer slope	5	30	17°49220¢S	149°92530¢W	30/03/2006
POL02	French Polynesia	Moorea	Opunohu Pass	MBIO-46	*Anampses*	*caeruleopunctatus*	Outer slope	5	40	17°49220¢S	149°92530¢W	30/03/2006
POL02	French Polynesia	Moorea	Opunohu Pass	MBIO-46	*Macropharyngodon*	*meleagris*	Outer slope	5	40	17°49220¢S	149°92530¢W	30/03/2006
POL02	French Polynesia	Moorea	Tiahura	MBIO-50	*Polylepion*	*russelli*	Outer slope	20	50	17°49220¢S	149°92530¢W	31/03/2006
REU01	Réunion	Hermitage	Canyon	SWIO-13	*Chaetodon*	*blackburni*	Outer slope	6	18	21°05768¢S	55°21333¢E	12/08/2007
REU01	Réunion	Hermitage	Canyon	SWIO-13	*Chaetodon*	*guttatissimus*	Outer slope	6	18	21°05768¢S	55°21333¢E	12/08/2007
REU01	Réunion	Hermitage	Canyon	SWIO-13	*Chaetodon*	*kleinii*	Outer slope	6	18	21°05768¢S	55°21333¢E	12/08/2007
REU01	Réunion	Hermitage	Canyon	SWIO-13	*Chaetodon*	*lunula*	Outer slope	6	18	21°05768¢S	55°21333¢E	12/08/2007
REU01	Réunion	Hermitage	Canyon	SWIO-13	*Chaetodon*	*meyeri*	Outer slope	6	18	21°05768¢S	55°21333¢E	12/08/2007
REU01	Réunion	Hermitage	Canyon	SWIO-13	*Chaetodon*	*trifascialis*	Outer slope	6	18	21°05768¢S	55°21333¢E	12/08/2007
REU01	Réunion	Hermitage	Canyon	SWIO-13	*Chaetodon*	*trifasciatus*	Outer slope	6	18	21°05768¢S	55°21333¢E	12/08/2007
REU01	Réunion	Hermitage	Canyon	SWIO-13	*Chaetodon*	*unimaculatus*	Outer slope	6	18	21°05768¢S	55°21333¢E	12/08/2007
REU01	Réunion	Hermitage	Canyon	SWIO-13	*Chaetodon*	*vagabundus*	Outer slope	6	18	21°05768¢S	55°21333¢E	12/08/2007
REU01	Réunion	Hermitage	Canyon	SWIO-13	*Chaetodon*	*xanthurus*	Outer slope	6	18	21°05768¢S	55°21333¢E	12/08/2007
REU01	Réunion	Hermitage	Canyon	SWIO-13	*Forcipiger*	*flavissimus*	Outer slope	6	18	21°05768¢S	55°21333¢E	12/08/2007
REU01	Réunion	Hermitage	Canyon	SWIO-13	*Hemitaurichthys*	*zoster*	Outer slope	6	18	21°05768¢S	55°21333¢E	12/08/2007
REU01	Réunion	Hermitage	Canyon	SWIO-13	*Heniochus*	*acuminatus*	Outer slope	6	18	21°05768¢S	55°21333¢E	12/08/2007
REU01	Réunion	Hermitage	Canyon	SWIO-13	*Heniochus*	*diphreutes*	Outer slope	6	18	21°05768¢S	55°21333¢E	12/08/2007
REU01	Réunion	Hermitage	Canyon	SWIO-13	*Anampses*	*caeruleopunctatus*	Outer slope	6	18	21°05768¢S	55°21333¢E	12/08/2007
REU01	Réunion	Hermitage	Canyon	SWIO-13	*Bodianus*	*anthioïdes*	Outer slope	6	18	21°05768¢S	55°21333¢E	12/08/2007
REU01	Réunion	Hermitage	Canyon	SWIO-13	*Bodianus*	*axillaris*	Outer slope	6	18	21°05768¢S	55°21333¢E	12/08/2007
REU01	Réunion	Hermitage	Canyon	SWIO-13	*Cheilinus*	*trilobatus*	Outer slope	6	18	21°05768¢S	55°21333¢E	12/08/2007
REU01	Réunion	Hermitage	Canyon	SWIO-13	*Coris*	*aygula*	Outer slope	6	18	21°05768¢S	55°21333¢E	12/08/2007
REU01	Réunion	Hermitage	Canyon	SWIO-13	*Coris*	*caudimacula*	Outer slope	6	18	21°05768¢S	55°21333¢E	12/08/2007
REU01	Réunion	Hermitage	Canyon	SWIO-13	*Coris*	*cuvieri*	Outer slope	6	18	21°05768¢S	55°21333¢E	12/08/2007
REU01	Réunion	Hermitage	Canyon	SWIO-13	*Gomphosus*	*caeruleus*	Outer slope	6	18	21°05768¢S	55°21333¢E	12/08/2007
REU01	Réunion	Hermitage	Canyon	SWIO-13	*Halichoeres*	*cosmetus*	Outer slope	6	18	21°05768¢S	55°21333¢E	12/08/2007
REU01	Réunion	Hermitage	Canyon	SWIO-13	*Halichoeres*	*hortulanus*	Outer slope	6	18	21°05768¢S	55°21333¢E	12/08/2007
REU01	Réunion	Hermitage	Canyon	SWIO-13	*Halichoeres*	*marginatus*	Outer slope	6	18	21°05768¢S	55°21333¢E	12/08/2007
REU01	Réunion	Hermitage	Canyon	SWIO-13	*Hemigymnus*	*fasciatus*	Outer slope	6	18	21°05768¢S	55°21333¢E	12/08/2007
REU01	Réunion	Hermitage	Canyon	SWIO-13	*Labroides*	*bicolor*	Outer slope	6	18	21°05768¢S	55°21333¢E	12/08/2007
REU01	Réunion	Hermitage	Canyon	SWIO-13	*Pseudocheilinus*	*octotaenia*	Outer slope	6	18	21°05768¢S	55°21333¢E	12/08/2007
REU01	Réunion	Hermitage	Canyon	SWIO-13	*Thalassoma*	*genivittatum*	Outer slope	6	18	21°05768¢S	55°21333¢E	12/08/2007
REU01	Réunion	Hermitage	Canyon	SWIO-13	*Amphiprion*	*chrysogaster*	Outer slope	6	18	21°05768¢S	55°21333¢E	12/08/2007
REU01	Réunion	Hermitage	Canyon	SWIO-13	*Chromis*	*chrysura*	Outer slope	6	18	21°05768¢S	55°21333¢E	12/08/2007
REU01	Réunion	Hermitage	Canyon	SWIO-13	*Chromis*	*dimidiata*	Outer slope	6	18	21°05768¢S	55°21333¢E	12/08/2007
REU01	Réunion	Hermitage	Canyon	SWIO-13	*Chromis*	*xanthochira*	Outer slope	6	18	21°05768¢S	55°21333¢E	12/08/2007
REU01	Réunion	Hermitage	Canyon	SWIO-13	*Plectroglyphidodon*	*dickii*	Outer slope	6	18	21°05768¢S	55°21333¢E	12/08/2007
REU01	Réunion	Hermitage	Canyon	SWIO-13	*Plectroglyphidodon*	*johnstonianus*	Outer slope	6	18	21°05768¢S	55°21333¢E	12/08/2007
REU01	Réunion	Hermitage	Canyon	SWIO-13	*Pomacentrus*	*agassizi*	Outer slope	6	18	21°05768¢S	55°21333¢E	12/08/2007
REU01	Réunion	Hermitage	Canyon	SWIO-13	*Pomacentrus*	*caeruleus*	Outer slope	6	18	21°05768¢S	55°21333¢E	12/08/2007
REU01	Réunion	Hermitage	Canyon	SWIO-13	*Stegastes*	*pelicieri*	Outer slope	6	18	21°05768¢S	55°21333¢E	12/08/2007
REU02	Réunion	St Leu	Sac jaune	SWIO-15	*Chaetodon*	*auriga*	Outer slope	12	19	21°15500¢S	55°28112¢E	13/08/2007
REU02	Réunion	St Leu	Sac jaune	SWIO-15	*Chaetodon*	*meyeri*	Outer slope	12	19	21°15500¢S	55°28112¢E	13/08/2007
REU02	Réunion	St Leu	Sac jaune	SWIO-15	*Heniochus*	*monoceros*	Outer slope	12	19	21°15500¢S	55°28112¢E	13/08/2007
REU02	Réunion	St Leu	Sac jaune	SWIO-15	*Anampses*	*caeruleopunctatus*	Outer slope	12	19	21°15500¢S	55°28112¢E	13/08/2007
REU02	Réunion	St Leu	Sac jaune	SWIO-15	*Anampses*	*lineatus*	Outer slope	12	19	21°15500¢S	55°28112¢E	13/08/2007
REU02	Réunion	St Leu	Sac jaune	SWIO-15	*Macropharyngodon*	*bipartitus*	Outer slope	12	19	21°15500¢S	55°28112¢E	13/08/2007
REU02	Réunion	St Leu	Sac jaune	SWIO-15	*Pseudocheilinus*	*octotaenia*	Outer slope	12	19	21°15500¢S	55°28112¢E	13/08/2007
REU02	Réunion	St Leu	Sac jaune	SWIO-15	*Thalassoma*	*genivittatum*	Outer slope	12	19	21°15500¢S	55°28112¢E	13/08/2007
REU02	Réunion	St Leu	Sac jaune	SWIO-15	*Abudefduf*	*margariteus*	Outer slope	12	19	21°15500¢S	55°28112¢E	13/08/2007
REU02	Réunion	St Leu	Sac jaune	SWIO-15	*Chromis*	*dimidiata*	Outer slope	12	19	21°15500¢S	55°28112¢E	13/08/2007
REU02	Réunion	St Leu	Sac jaune	SWIO-15	*Dascyllus*	*trimaculatus*	Outer slope	12	19	21°15500¢S	55°28112¢E	13/08/2007
REU02	Réunion	St Leu	Sac jaune	SWIO-15	*Plectroglyphidodon*	*dickii*	Outer slope	12	19	21°15500¢S	55°28112¢E	13/08/2007
REU02	Réunion	St Leu	Sac jaune	SWIO-15	*Pomacentrus*	*agassizi*	Outer slope	12	19	21°15500¢S	55°28112¢E	13/08/2007
REU02	Réunion	St Leu	Sac jaune	SWIO-15	*Pomacentrus*	*caeruleus*	Outer slope	12	19	21°15500¢S	55°28112¢E	13/08/2007
REU02	Réunion	St Leu	Maison verte/Cimetière	SWIO-16	*Chaetodon*	*guttatissimus*	Outer slope	8	16	21°19358¢S	55°28243¢E	13/08/2007
REU02	Réunion	St Leu	Maison verte/Cimetière	SWIO-16	*Chaetodon*	*kleinii*	Outer slope	8	16	21°19358¢S	55°28243¢E	13/08/2007
REU02	Réunion	St Leu	Maison verte/Cimetière	SWIO-16	*Chaetodon*	*trifasciatus*	Outer slope	8	16	21°19358¢S	55°28243¢E	13/08/2007
REU02	Réunion	St Leu	Maison verte/Cimetière	SWIO-16	*Forcipiger*	*flavissimus*	Outer slope	8	16	21°19358¢S	55°28243¢E	13/08/2007
REU02	Réunion	St Leu	Maison verte/Cimetière	SWIO-16	*Bodianus*	*axillaris*	Outer slope	8	16	21°19358¢S	55°28243¢E	13/08/2007
REU02	Réunion	St Leu	Maison verte/Cimetière	SWIO-16	*Gomphosus*	*caeruleus*	Outer slope	8	16	21°19358¢S	55°28243¢E	13/08/2007
REU02	Réunion	St Leu	Maison verte/Cimetière	SWIO-16	*Halichoeres*	*cosmetus*	Outer slope	8	16	21°19358¢S	55°28243¢E	13/08/2007
REU02	Réunion	St Leu	Maison verte/Cimetière	SWIO-16	*Halichoeres*	*marginatus*	Outer slope	8	16	21°19358¢S	55°28243¢E	13/08/2007
REU02	Réunion	St Leu	Maison verte/Cimetière	SWIO-16	*Pseudocheilinus*	*octotaenia*	Outer slope	8	16	21°19358¢S	55°28243¢E	13/08/2007
REU02	Réunion	St Leu	Maison verte/Cimetière	SWIO-16	*Stethojulis*	*albovittata*	Outer slope	8	16	21°19358¢S	55°28243¢E	13/08/2007
REU02	Réunion	St Leu	Maison verte/Cimetière	SWIO-16	*Abudefduf*	*margariteus*	Outer slope	8	16	21°19358¢S	55°28243¢E	13/08/2007
REU02	Réunion	St Leu	Maison verte/Cimetière	SWIO-16	*Abudefduf*	*sparoides*	Outer slope	8	16	21°19358¢S	55°28243¢E	13/08/2007
REU02	Réunion	St Leu	Maison verte/Cimetière	SWIO-16	*Chromis*	*chrysura*	Outer slope	8	16	21°19358¢S	55°28243¢E	13/08/2007
REU02	Réunion	St Leu	Maison verte/Cimetière	SWIO-16	*Chromis*	*dimidiata*	Outer slope	8	16	21°19358¢S	55°28243¢E	13/08/2007
REU02	Réunion	St Leu	Maison verte/Cimetière	SWIO-16	*Chromis*	*nigrura*	Outer slope	8	16	21°19358¢S	55°28243¢E	13/08/2007
REU02	Réunion	St Leu	Maison verte/Cimetière	SWIO-16	*Dascyllus*	*trimaculatus*	Outer slope	8	16	21°19358¢S	55°28243¢E	13/08/2007
REU02	Réunion	St Leu	Maison verte/Cimetière	SWIO-16	*Plectroglyphidodon*	*johnstonianus*	Outer slope	8	16	21°19358¢S	55°28243¢E	13/08/2007
REU02	Réunion	St Leu	Maison verte/Cimetière	SWIO-16	*Pomacentrus*	*agassizi*	Outer slope	8	16	21°19358¢S	55°28243¢E	13/08/2007
REU02	Réunion	St Leu	Maison verte/Cimetière	SWIO-16	*Stegastes*	*pelicieri*	Outer slope	8	16	21°19358¢S	55°28243¢E	13/08/2007
REU02	Réunion	St Leu	Sac jaune	SWIO-23	*Chaetodon*	*lunula*	Outer slope	12	19	21°15500¢S	55°28112¢E	13/08/2007
REU02	Réunion	St Leu	Sac jaune	SWIO-23	*Chaetodon*	*meyeri*	Outer slope	12	19	21°15500¢S	55°28112¢E	13/08/2007
REU02	Réunion	St Leu	Sac jaune	SWIO-23	*Chaetodon*	*trifasciatus*	Outer slope	12	19	21°15500¢S	55°28112¢E	13/08/2007
REU02	Réunion	St Leu	Sac jaune	SWIO-23	*Chaetodon*	*xanthurus*	Outer slope	12	19	21°15500¢S	55°28112¢E	13/08/2007
REU02	Réunion	St Leu	Sac jaune	SWIO-23	*Bodianus*	*axillaris*	Outer slope	12	19	21°15500¢S	55°28112¢E	13/08/2007
REU02	Réunion	St Leu	Sac jaune	SWIO-23	*Labroides*	*dimidiatus*	Outer slope	12	19	21°15500¢S	55°28112¢E	13/08/2007
REU02	Réunion	St Leu	Sac jaune	SWIO-23	*Thalassoma*	*genivittatum*	Outer slope	12	19	21°15500¢S	55°28112¢E	13/08/2007
REU03	Réunion	Hermitage	Varangue du Lagon	SWIO-02	*Chaetodon*	*auriga*	Inner reef	0	2	21°07473¢S	55°22018¢E	10/08/2007
REU03	Réunion	Hermitage	Varangue du Lagon	SWIO-02	*Chaetodon*	*guttatissimus*	Inner reef	0	2	21°07473¢S	55°22018¢E	10/08/2007
REU03	Réunion	Hermitage	Varangue du Lagon	SWIO-02	*Chaetodon*	*lunula*	Inner reef	0	2	21°07473¢S	55°22018¢E	10/08/2007
REU03	Réunion	Hermitage	Varangue du Lagon	SWIO-02	*Chaetodon*	*melannotus*	Inner reef	0	2	21°07473¢S	55°22018¢E	10/08/2007
REU03	Réunion	Hermitage	Varangue du Lagon	SWIO-02	*Gomphosus*	*caeruleus*	Inner reef	0	2	21°07473¢S	55°22018¢E	10/08/2007
REU03	Réunion	Hermitage	Varangue du Lagon	SWIO-02	*Thalassoma*	*hardwicke*	Inner reef	0	2	21°07473¢S	55°22018¢E	10/08/2007
REU03	Réunion	Hermitage	Varangue du Lagon	SWIO-02	*Chromis*	*viridis*	Inner reef	0	2	21°07473¢S	55°22018¢E	10/08/2007
REU03	Réunion	Hermitage	Trou d'eau	SWIO-06	*Chaetodon*	*melannotus*	Inner reef	0	2	21°10082¢S	55°24372¢E	08/08/2007
REU03	Réunion	Hermitage	unknown	SWIO-21	*Thalassoma*	*purpureum*	Inner reef	0	2	21°36451¢S	55°76776¢E	20/07/2007
REU03	Réunion	Hermitage	unknown	SWIO-21	*Abudefduf*	*sordidus*	Inner reef	0	2	21°36451¢S	55°76776¢E	20/07/2007
REU03	Réunion	Hermitage	Planche Alizée	SWIO-29	*Chaetodon*	*lunula*	Inner reef	0	2	21°09408¢S	55°23472¢E	20/07/2007
REU03	Réunion	Hermitage	Planche Alizée	SWIO-29	*Chaetodon*	*trifasciatus*	Inner reef	0	2	21°09408¢S	55°23472¢E	20/07/2007
REU03	Réunion	Hermitage	Planche Alizée	SWIO-29	*Halichoeres*	*scapularis*	Inner reef	0	2	21°09408¢S	55°23472¢E	20/07/2007
REU03	Réunion	Hermitage	Planche Alizée	SWIO-29	*Thalassoma*	*genivittatum*	Inner reef	0	2	21°09408¢S	55°23472¢E	20/07/2007
REU03	Réunion	Hermitage	Planche Alizée	SWIO-29	*Chromis*	*viridis*	Inner reef	0	2	21°09408¢S	55°23472¢E	20/07/2007
REU03	Réunion	Hermitage	Planche Alizée	SWIO-29	*Chrysiptera*	*glauca*	Inner reef	0	2	21°09408¢S	55°23472¢E	20/07/2007
REU03	Réunion	Hermitage	Planche Alizée	SWIO-29	*Dascyllus*	*aruanus*	Inner reef	0	2	21°09408¢S	55°23472¢E	20/07/2007
REU03	Réunion	Hermitage	Planche Alizée	SWIO-29	*Chrysiptera*	*unimaculata*	Inner reef	0	2	21°09408¢S	55°23472¢E	20/07/2007
REU03	Réunion	Hermitage	Planche Alizée	SWIO-29	*Stegastes*	*limbatus*	Inner reef	0	2	21°09408¢S	55°23472¢E	20/07/2007
REU03	Réunion	Hermitage	Planche Alizée	SWIO-29	*Stegastes*	*lividus*	Inner reef	0	2	21°09408¢S	55°23472¢E	20/07/2007
REU03	Réunion	Hermitage	Planche Alizée	SWIO-29	*Stegastes*	*nigricans*	Inner reef	0	2	21°09408¢S	55°23472¢E	20/07/2007
REU03	Réunion	Hermitage	Chez Go	SWIO-32	*Chaetodon*	*auriga*	Inner reef	0	2	21°07875¢S	55°22555¢E	24/07/2007
REU03	Réunion	Hermitage	Chez Go	SWIO-32	*Chaetodon*	*guttatissimus*	Inner reef	0	2	21°07875¢S	55°22555¢E	24/07/2007
REU03	Réunion	Hermitage	Chez Go	SWIO-32	*Chaetodon*	*kleinii*	Inner reef	0	2	21°07875¢S	55°22555¢E	24/07/2007
REU03	Réunion	Hermitage	Chez Go	SWIO-32	*Chaetodon*	*trifascialis*	Inner reef	0	2	21°07875¢S	55°22555¢E	24/07/2007
REU03	Réunion	Hermitage	Chez Go	SWIO-32	*Chaetodon*	*vagabundus*	Inner reef	0	2	21°07875¢S	55°22555¢E	24/07/2007
REU03	Réunion	Hermitage	Chez Go	SWIO-32	*Heniochus*	*monoceros*	Inner reef	0	2	21°07875¢S	55°22555¢E	24/07/2007
REU03	Réunion	Hermitage	Chez Go	SWIO-32	*Cheilinus*	*chlorourus*	Inner reef	0	2	21°07875¢S	55°22555¢E	24/07/2007
REU03	Réunion	Hermitage	Chez Go	SWIO-32	*Cheilinus*	*trilobatus*	Inner reef	0	2	21°07875¢S	55°22555¢E	24/07/2007
REU03	Réunion	Hermitage	Chez Go	SWIO-32	*Halichoeres*	*marginatus*	Inner reef	0	2	21°07875¢S	55°22555¢E	24/07/2007
REU03	Réunion	Hermitage	Chez Go	SWIO-32	*Stethojulis*	*albovittata*	Inner reef	0	2	21°07875¢S	55°22555¢E	24/07/2007
REU03	Réunion	Hermitage	Chez Go	SWIO-32	*Thalassoma*	*genivittatum*	Inner reef	0	2	21°07875¢S	55°22555¢E	24/07/2007
REU03	Réunion	Hermitage	Chez Go	SWIO-32	*Plectroglyphidodon*	*dickii*	Inner reef	0	2	21°07875¢S	55°22555¢E	24/07/2007
REU03	Réunion	Hermitage	Chez Go	SWIO-32	*Plectroglyphidodon*	*johnstonianus*	Inner reef	0	2	21°07875¢S	55°22555¢E	24/07/2007
REU03	Réunion	Hermitage	Chez Go	SWIO-32	*Stegastes*	*nigricans*	Inner reef	0	2	21°07875¢S	55°22555¢E	24/07/2007
REU03	Réunion	Hermitage	Snack 46	SWIO-33	*Chaetodon*	*trifascialis*	Inner reef	0	2	21°17693¢S	55°28730¢E	26/07/2007
REU03	Réunion	Hermitage	Snack 46	SWIO-33	*Chaetodon*	*unimaculatus*	Inner reef	0	2	21°17693¢S	55°28730¢E	26/07/2007
REU03	Réunion	Hermitage	Snack 46	SWIO-33	*Gomphosus*	*caeruleus*	Inner reef	0	2	21°17693¢S	55°28730¢E	26/07/2007
REU03	Réunion	Hermitage	Snack 46	SWIO-33	*Halichoeres*	*marginatus*	Inner reef	0	2	21°17693¢S	55°28730¢E	26/07/2007
REU03	Réunion	Hermitage	Snack 46	SWIO-33	*Stethojulis*	*albovittata*	Inner reef	0	2	21°17693¢S	55°28730¢E	26/07/2007
REU03	Réunion	Hermitage	Snack 46	SWIO-33	*Abudefduf*	*sparoides*	Inner reef	0	2	21°17693¢S	55°28730¢E	26/07/2007
REU03	Réunion	Hermitage	Snack 46	SWIO-33	*Chrysiptera*	*glauca*	Inner reef	0	2	21°17693¢S	55°28730¢E	26/07/2007
REU03	Réunion	Hermitage	Snack 46	SWIO-33	*Chrysiptera*	*unimaculata*	Inner reef	0	2	21°17693¢S	55°28730¢E	26/07/2007
REU03	Réunion	Hermitage	Trou d'eau	SWIO-34	*Chaetodon*	*guttatissimus*	Inner reef	0	2	21°10082¢S	55°24372¢E	27.07/2007
REU03	Réunion	Hermitage	Trou d'eau	SWIO-34	*Chaetodon*	*lunula*	Inner reef	0	2	21°10082¢S	55°24372¢E	27.07/2007
REU03	Réunion	Hermitage	Trou d'eau	SWIO-34	*Chaetodon*	*melannotus*	Inner reef	0	2	21°10082¢S	55°24372¢E	27.07/2007
REU03	Réunion	Hermitage	Trou d'eau	SWIO-34	*Chaetodon*	*trifascialis*	Inner reef	0	2	21°10082¢S	55°24372¢E	27.07/2007
REU03	Réunion	Hermitage	Trou d'eau	SWIO-34	*Chaetodon*	*vagabundus*	Inner reef	0	2	21°10082¢S	55°24372¢E	27.07/2007
REU03	Réunion	Hermitage	Trou d'eau	SWIO-34	*Heniochus*	*monoceros*	Inner reef	0	2	21°10082¢S	55°24372¢E	27.07/2007
REU03	Réunion	Hermitage	Trou d'eau	SWIO-34	*Abudefduf*	*sparoides*	Inner reef	0	2	21°10082¢S	55°24372¢E	27.07/2007
REU03	Réunion	Hermitage	Trou d'eau	SWIO-34	*Chromis*	*viridis*	Inner reef	0	2	21°10082¢S	55°24372¢E	27.07/2007
REU03	Réunion	Hermitage	Trou d'eau	SWIO-34	*Pomacentrus*	*agassizi*	Inner reef	0	2	21°10082¢S	55°24372¢E	27.07/2007
REU03	Réunion	Hermitage	Trou d'eau	SWIO-34	*Stegastes*	*pelicieri*	Inner reef	0	2	21°10082¢S	55°24372¢E	27.07/2007
REU03	Réunion	Hermitage	Les villas du Lagon	SWIO-35	*Chaetodon*	*auriga*	Inner reef	0	2	21°08950¢S	55°23116¢E	01/08/2007
REU03	Réunion	Hermitage	Les villas du Lagon	SWIO-35	*Halichoeres*	*marginatus*	Inner reef	0	2	21°08950¢S	55°23116¢E	01/08/2007
REU03	Réunion	Hermitage	Les villas du Lagon	SWIO-35	*Stethojulis*	*albovittata*	Inner reef	0	2	21°08950¢S	55°23116¢E	01/08/2007
REU03	Réunion	Hermitage	Toboggan	SWIO-36	*Chaetodon*	*kleinii*	Inner reef	0	2	21°07875¢S	55°22555¢E	24/07/2007
REU03	Réunion	Hermitage	Toboggan	SWIO-36	*Heniochus*	*monoceros*	Inner reef	0	2	21°07875¢S	55°22555¢E	24/07/2007
REU03	Réunion	Hermitage	Toboggan	SWIO-36	*Halichoeres*	*scapularis*	Inner reef	0	2	21°07875¢S	55°22555¢E	24/07/2007
REU03	Réunion	Hermitage	Toboggan	SWIO-36	*Stethojulis*	*albovittata*	Inner reef	0	2	21°07875¢S	55°22555¢E	24/07/2007
REU03	Réunion	Hermitage	Toboggan	SWIO-36	*Chrysiptera*	*glauca*	Inner reef	0	2	21°07875¢S	55°22555¢E	24/07/2007
REU03	Réunion	Hermitage	Trou d'eau	SWIO-40	*Halichoeres*	*marginatus*	Inner reef	0	2	21°10082¢S	55°24372¢E	26/07/2007
REU03	Réunion	Hermitage	Trou d'eau	SWIO-40	*Stethojulis*	*albovittata*	Inner reef	0	2	21°10082¢S	55°24372¢E	26/07/2007
REU03	Réunion	Hermitage	Trou d'eau	SWIO-40	*Stethojulis*	*albovittata*	Inner reef	0	2	21°10082¢S	55°24372¢E	26/07/2007
REU03	Réunion	Hermitage	Trou d'eau	SWIO-40	*Thalassoma*	*genivittatum*	Inner reef	0	2	21°10082¢S	55°24372¢E	26/07/2007
REU03	Réunion	Hermitage	Trou d'eau	SWIO-40	*Chrysiptera*	*brownriggii*	Inner reef	0	2	21°10082¢S	55°24372¢E	26/07/2007
REU03	Réunion	Hermitage	Trou d'eau	SWIO-40	*Chrysiptera*	*glauca*	Inner reef	0	2	21°10082¢S	55°24372¢E	26/07/2007
REU03	Réunion	Hermitage	Trou d'eau	SWIO-40	*Dascyllus*	*aruanus*	Inner reef	0	2	21°10082¢S	55°24372¢E	26/07/2007
REU03	Réunion	Hermitage	Trou d'eau	SWIO-40	*Plectroglyphidodon*	*imparipennis*	Inner reef	0	2	21°10082¢S	55°24372¢E	26/07/2007
REU03	Réunion	Hermitage	Trou d'eau	SWIO-40	*Plectroglyphidodon*	*randalli*	Inner reef	0	2	21°10082¢S	55°24372¢E	26/07/2007
REU03	Réunion	Hermitage	Trou d'eau	SWIO-40	*Chrysiptera*	*unimaculata*	Inner reef	0	2	21°10082¢S	55°24372¢E	26/07/2007
REU03	Réunion	Hermitage	Trou d'eau	SWIO-40	*Stegastes*	*limbatus*	Inner reef	0	2	21°10082¢S	55°24372¢E	26/07/2007
REU03	Réunion	Hermitage	Trou d'eau	SWIO-40	*Stegastes*	*lividus*	Inner reef	0	2	21°10082¢S	55°24372¢E	26/07/2007
REU04	Réunion	St Philippe	Vincendo	SWIO-30	*Halichoeres*	*marginatus*	Inner reef	0	2	21°36451¢S	55°76776¢E	20/07/2007
REU04	Réunion	St Philippe	Vincendo	SWIO-30	*Thalassoma*	*genivittatum*	Inner reef	0	2	21°36451¢S	55°76776¢E	20/07/2007
REU04	Réunion	St Philippe	Vincendo	SWIO-30	*Thalassoma*	*purpureum*	Inner reef	0	2	21°36451¢S	55°76776¢E	20/07/2007
REU04	Réunion	St Philippe	Vincendo	SWIO-30	*Abudefduf*	*sordidus*	Inner reef	0	2	21°36451¢S	55°76776¢E	20/07/2007
REU04	Réunion	St Philippe	Vincendo	SWIO-30	*Abudefduf*	*vaigiensis*	Inner reef	0	2	21°36451¢S	55°76776¢E	20/07/2007
REU04	Réunion	St Philippe	Vincendo	SWIO-30	*Plectroglyphidodon*	*imparipennis*	Inner reef	0	2	21°36451¢S	55°76776¢E	20/07/2007
REU04	Réunion	St Philippe	Vincendo	SWIO-30	*Plectroglyphidodon*	*phoenixensis*	Inner reef	0	2	21°36451¢S	55°76776¢E	20/07/2007
REU04	Réunion	St Philippe	Vincendo	SWIO-30	*Plectroglyphidodon*	*randalli*	Inner reef	0	2	21°36451¢S	55°76776¢E	20/07/2007
REU05	Réunion	St Leu	Kiosque	SWIO-41	*Chaetodon*	*trifascialis*	Inner reef	0	2	21°18323¢S	55°01127¢E	14/11/2007
REU05	Réunion	St Leu	Kiosque	SWIO-41	*Chaetodon*	*trifasciatus*	Inner reef	0	2	21°18323¢S	55°01127¢E	14/11/2007
REU05	Réunion	St Leu	Kiosque	SWIO-41	*Chaetodon*	*unimaculatus*	Inner reef	0	2	21°18323¢S	55°01127¢E	14/11/2007
REU05	Réunion	St Leu	Kiosque	SWIO-41	*Epibulus*	*insidiator*	Inner reef	0	2	21°18323¢S	55°01127¢E	14/11/2007
REU05	Réunion	St Leu	Kiosque	SWIO-41	*Gomphosus*	*caeruleus*	Inner reef	0	2	21°18323¢S	55°01127¢E	14/11/2007
REU05	Réunion	St Leu	Kiosque	SWIO-41	*Halichoeres*	*scapularis*	Inner reef	0	2	21°18323¢S	55°01127¢E	14/11/2007
REU05	Réunion	St Leu	Kiosque	SWIO-41	*Thalassoma*	*genivittatum*	Inner reef	0	2	21°18323¢S	55°01127¢E	14/11/2007
REU05	Réunion	St Leu	Kiosque	SWIO-41	*Thalassoma*	*hardwicke*	Inner reef	0	2	21°18323¢S	55°01127¢E	14/11/2007
REU05	Réunion	St Leu	Kiosque	SWIO-41	*Thalassoma*	*hardwicke*	Inner reef	0	2	21°18323¢S	55°01127¢E	14/11/2007
REU05	Réunion	St Leu	Kiosque	SWIO-41	*Chromis*	*viridis*	Inner reef	0	2	21°18323¢S	55°01127¢E	14/11/2007
REU05	Réunion	St Leu	Kiosque	SWIO-41	*Chrysiptera*	*glauca*	Inner reef	0	2	21°18323¢S	55°01127¢E	14/11/2007
REU05	Réunion	St Leu	Kiosque	SWIO-41	*Dascyllus*	*aruanus*	Inner reef	0	2	21°18323¢S	55°01127¢E	14/11/2007
REU05	Réunion	St Leu	Kiosque	SWIO-41	*Plectroglyphidodon*	*randalli*	Inner reef	0	2	21°18323¢S	55°01127¢E	14/11/2007
REU05	Réunion	St Leu	Kiosque	SWIO-41	*Stegastes*	*limbatus*	Inner reef	0	2	21°18323¢S	55°01127¢E	14/11/2007
REU05	Réunion	St Leu	Kiosque	SWIO-41	*Stegastes*	*lividus*	Inner reef	0	2	21°18323¢S	55°01127¢E	14/11/2007
REU05	Réunion	St Leu	Kiosque	SWIO-41	*Stegastes*	*nigricans*	Inner reef	0	2	21°18323¢S	55°01127¢E	14/11/2007

**Appendix 2:** Species of Chaetodontidae, Pomacentridae, and Labridae included in the present study with their respective outgroups used in phylogenetic analyses and associated GenBank and Barcode of Life Database (BOLD) accession numbers

Family	Genus	Species	Site	Sample ID	Process ID (BOLD)	COI	16S	Cytb
Lutjanidae	*Lutjanus*	*sebae*	–	–	–	NC012736	NC012736	NC012736
Lutjanidae	*Lutjanus*	*malabaricus*	–	–	–	NC012737	NC012737	NC012737
Serranidae	*Anyperodon*	*leucogrammicus*	-	-	-	NC012709	NC012709	NC012709
Serranidae	*Epinephelus*	*akaara*	-	-	-	NC011113	NC011113	NC011113
Cichlidae	*Hypselecara*	*temporalis*	-	-	-	NC011168	NC011168	NC011168
Cichlidae	*Tropheus*	*duboisi*	-	-	-	AP006015	AP006015	AP006015
Cichlidae	*Astronotus*	*ocellatus*	–	–	–	NC009058	NC009058	NC009058
Cichlidae	*Neolamprologus*	*brichardi*	–	–	–	NC009062	NC009062	NC009062
Cichlidae	*Tylochromis*	*polylepis*	–	–	–	AP009509	AP009509	AP009509
Cichlidae	*Paratilapia*	*polleni*	–	–	–	AP009508	AP009508	AP009508
Cichlidae	*Ptychochromoides*	*katria*	–	–	–	AP009507	AP009507	AP009507
Cichlidae	*Etroplus*	*maculatus*	–	–	–	AP009505	AP009505	AP009505
Cichlidae	*Paretroplus*	*maculatus*	–	–	–	AP009504	AP009504	AP009504
Embiotocidae	*Ditrema*	*temminckii*	–	–	–	NC009060	NC009060	NC009060
Embiotocidae	*Cymatogaster*	*aggregata*	–	–	–	NC009059	NC009059	NC009059
Pseudochromidae	*Labracinus*	*cyclophtlamus*	–	–	–	NC009054	NC009054	NC009054
Pomacanthidae	*Centropyge*	*loricula*	–	–	–	NC009872	NC009872	NC009872
Pomacanthidae	*Chaetodontoplus*	*septentrionalis*	–	–	–	NC009873	NC009873	NC009873
Pomacentridae	*Abudefduf*	*margariteus**	SWIO-15	REU0925	IPCOM423-10	JF434705	JF457203	JF457873
Pomacentridae	*Abudefduf*	*margariteus*	SWIO-16	REU0981	IPCOM437-10	JF434704	JF457202	JF457872
Pomacentridae	*Abudefduf*	*septemfasciatus*	MGNW-01	NBE0017	IPCOM095-10	JF434706	JF457204	JF457874
Pomacentridae	*Abudefduf*	*sexfasciatus*	MGNW-03	NBE0184	IPCOM142-10	JF434708	JF457206	JF457876
Pomacentridae	*Abudefduf*	*sexfasciatus*	MGNW-03	NBE0185	IPCOM143-10	JF434707	JF457205	JF457875
Pomacentridae	*Abudefduf*	*sordidus**	MGNW-10	NBE0669	IPCOM258-10	JF434713	JF457211	JF457881
Pomacentridae	*Abudefduf*	*sordidus*	MGNW-10	NBE0670	IPCOM259-10	JF434712	JF457210	JF457880
Pomacentridae	*Abudefduf*	*sordidus*	MGNW-10	NBE0671	IPCOM260-10	JF434711	JF457209	JF457879
Pomacentridae	*Abudefduf*	*sordidus*	SWIO-21	REU1682	IPCOM470-10	JF434710	JF457208	JF457878
Pomacentridae	*Abudefduf*	*sordidus*	SWIO-30	REU1686	IPCOM473-10	JF434709	JF457207	JF457877
Pomacentridae	*Abudefduf*	*sparoides*	MGNW-03	NBE0198	IPCOM149-10	JF434716	JF457214	JF457884
Pomacentridae	*Abudefduf*	*sparoides*	MGNW-10	NBE0640	IPCOM246-10	JF434715	JF457213	JF457883
Pomacentridae	*Abudefduf*	*sparoides*	SWIO-16	REU0969	IPCOM434-10	JF434719	JF457217	JF457887
Pomacentridae	*Abudefduf*	*sparoides*	SWIO-16	REU0970	IPCOM435-10	JF434718	JF457216	JF457886
Pomacentridae	*Abudefduf*	*sparoides*	SWIO-16	REU0971	IPCOM436-10	JF434717	JF457215	JF457885
Pomacentridae	*Abudefduf*	*sparoides*	SWIO-33	REU195-1	IPCOM487-10	JF434714	JF457212	JF457882
Pomacentridae	*Abudefduf*	*vaigiensis*	MGNW-04	NBE0251	IPCOM168-10	JF434721	JF457219	JF457889
Pomacentridae	*Abudefduf*	*vaigiensis*	SWIO-30	REU1687	IPCOM474-10	JF434720	JF457218	JF457888
Pomacentridae	*Amblyglyphidodon*	*indicus**	MGNW-01	NBE0025	IPCOM098-10	JF434725	JF457223	JF457893
Pomacentridae	*Amblyglyphidodon*	*indicus*	MGNW-10	NBE0601	IPCOM231-10	JF434724	JF457222	JF457892
Pomacentridae	*Amblyglyphidodon*	*indicus*	MGNW-10	NBE0602	IPCOM232-10	JF434723	JF457221	JF457891
Pomacentridae	*Amblyglyphidodon*	*indicus*	MGNW-20	NBE1321	IPCOM319-10	JF434722	JF457220	JF457890
Pomacentridae	*Amphiprion*	*akallopisos**	MGNW-12	NBE1011	IPCOM264-10	JF434730	JF457228	JF457898
Pomacentridae	*Amphiprion*	*akallopisos*	MGNW-12	NBE1012	IPCOM265-10	JF434729	JF457227	JF457897
Pomacentridae	*Amphiprion*	*akallopisos*	MGNW-12	NBE1013	IPCOM266-10	JF434728	JF457226	JF457896
Pomacentridae	*Amphiprion*	*akallopisos*	MGNW-13	NBE1036	IPCOM272-10	JF434727	JF457225	JF457895
Pomacentridae	*Amphiprion*	*akallopisos*	MGNW-13	NBE1037	IPCOM273-10	JF434726	JF457224	JF457894
Pomacentridae	*Amphiprion*	*chrysogaster*	SWIO-13	REU0702	IPCOM356-10	JF434731	JF457229	JF457899
Pomacentridae	*Amphiprion*	*chrysopterus*	MBIO-15	MBIO1219	IPCOM068-10	JF434733	JF457231	JF457901
Pomacentridae	*Amphiprion*	*chrysopterus*	MBIO-15	MBIO1220	IPCOM069-10	JF434732	JF457230	JF457900
Pomacentridae	*Amphiprion*	*latifasciatus*	MGNW-03	NBE0191	IPCOM147-10	JF434737	JF457235	JF457905
Pomacentridae	*Amphiprion*	*latifasciatus*	MGNW-16	NBE1138	IPCOM291-10	JF434736	JF457234	JF457904
Pomacentridae	*Amphiprion*	*latifasciatus*	MGNW-16	NBE1139	IPCOM292-10	JF434735	JF457233	JF457903
Pomacentridae	*Amphiprion*	*latifasciatus*	MGNW-19	NBE1272	IPCOM307-10	JF434734	JF457232	JF457902
Labridae	*Anampses*	*caeruleopunctatus**	MGNW-10	NBE0639	IPCOM245-10	JF434738	JF457236	JF457906
Labridae	*Anampses*	*caeruleopunctatus*	SWIO-15	REU0919	IPCOM419-10	JF434739	JF457237	-
Labridae	*Anampses*	*lineatus*	SWIO-15	REU0922	IPCOM422-10	JF434740	JF457238	JF457908
Labridae	*Anampses*	*twistii*	MGNW-01	NBE0034	IPCOM104-10	-	JF457244	JF457914
Labridae	*Anampses*	*twistii*	MGNW-10	NBE0637	IPCOM244-10	JF434745	JF457243	JF457913
Labridae	*Anampses*	*meleagrides*	MGNW-16	NBE1125	IPCOM289-10	JF434744	JF457242	JF457912
Labridae	*Anampses*	*meleagrides*	MGNW-19	NBE1286	IPCOM312-10	JF434743	JF457241	JF457911
Labridae	*Anampses*	*twistii*	MGNW-19	NBE1287	IPCOM313-10	JF434742	JF457240	JF457910
Labridae	*Anampses*	*meleagrides*	MGNW-19	NBE1288	IPCOM314-10	JF434741	JF457239	JF457909
Labridae	*Bodianus*	*anthioïdes**	MGNW-16	NBE1113	IPCOM280-10	JF434747	JF457246	JF457916
Labridae	*Bodianus*	*anthioïdes*	MGNW-16	NBE1114	IPCOM281-10	JF434746	JF457245	JF457915
Labridae	*Bodianus*	*axillaris*	MGNW-01	NBE0074	IPCOM127-10	JF434754	JF457253	JF457923
Labridae	*Bodianus*	*axillaris*	MGNW-01	NBE0075	IPCOM128-10	JF434753	JF457252	JF457922
Labridae	*Bodianus*	*axillaris*	MGNW-05	NBE0356	IPCOM185-10	JF434752	JF457251	JF457921
Labridae	*Bodianus*	*axillaris*	SWIO-13	REU0735	IPCOM377-10	JF434751	JF457250	JF457920
Labridae	*Bodianus*	*axillaris*	SWIO-13	REU0775	IPCOM404-10	JF434750	JF457249	JF457919
Labridae	*Bodianus*	*axillaris*	SWIO-16	REU0995	IPCOM441-10	JF434749	JF457248	JF457918
Labridae	*Bodianus*	*axillaris*	SWIO-23	REU1617	IPCOM465-10	JF434748	JF457247	JF457917
Labridae	*Bodianus*	*diana**	MGNW-05	NBE0365	IPCOM191-10	JF434759	JF457258	JF457928
Labridae	*Bodianus*	*diana*	MGNW-16	NBE1115	IPCOM282-10	JF434758	JF457257	JF457927
Labridae	*Bodianus*	*diana*	MGNW-16	NBE1116	IPCOM283-10	JF434757	JF457256	JF457926
Labridae	*Bodianus*	*diana*	MGNW-16	NBE1117	IPCOM284-10	JF434756	JF457255	JF457925
Labridae	*Bodianus*	*diana*	MGNW-16	NBE1118	IPCOM285-10	JF434755	JF457254	JF457924
Labridae	*Bodianus*	*perditio*	MGNW-16	NBE1140	IPCOM293-10	JF434760	JF457259	JF457929
Chaetodontidae	*Chaetodon*	*auriga*	MGNW-04	NBE0211	IPCOM154-10	JF434764	JF457263	JF457933
Chaetodontidae	*Chaetodon*	*auriga*	SWIO-02	REU0112	IPCOM340-10	JF434763	JF457262	JF457932
Chaetodontidae	*Chaetodon*	*auriga*	SWIO-15	REU0903	IPCOM414-10	JF434765	JF457264	JF457934
Chaetodontidae	*Chaetodon*	*auriga**	SWIO-32	REU151-1	IPCOM457-10	JF434762	JF457261	JF457931
Chaetodontidae	*Chaetodon*	*auriga*	SWIO-35	REU301-1	IPCOM513-10	JF434761	JF457260	JF457930
Chaetodontidae	*Chaetodon*	*bennetti*	MBIO-23	MBIO1310	IPCOM082-10	JF434768	JF457267	-
Chaetodontidae	*Chaetodon*	*bennetti**	MGNW-07	NBE0453	IPCOM216-10	JF434767	JF457266	JF457936
Chaetodontidae	*Chaetodon*	*bennetti*	MGNW-07	NBE0454	IPCOM217-10	JF434766	JF457265	JF457935
Chaetodontidae	*Chaetodon*	*blackburni**	SWIO-13	REU0754	IPCOM392-10	JF434770	JF457269	JF457938
Chaetodontidae	*Chaetodon*	*blackburni*	SWIO-13	REU0755	IPCOM393-10	JF434769	JF457268	JF457937
Chaetodontidae	*Chaetodon*	*citrinellus*	MBIO-05	MBIO0513	IPCOM024-10	JF434772	JF457271	JF457940
Chaetodontidae	*Chaetodon*	*citrinellus*	MBIO-07	MBIO0856	IPCOM049-10	JF434771	JF457270	JF457939
Chaetodontidae	*Chaetodon*	*ephippium*	MBIO-17	MBIO1261	IPCOM073-10	JF434773	JF457272	-
Chaetodontidae	*Chaetodon*	*falcula*	MGNW-01	NBE0042	IPCOM111-10	JF434776	JF457275	JF457943
Chaetodontidae	*Chaetodon*	*falcula*	MGNW-07	NBE0455	IPCOM218-10	JF434775	JF457274	JF457942
Chaetodontidae	*Chaetodon*	*falcula*	MGNW-39	NBE2035	IPCOM328-10	JF434774	JF457273	JF457941
Chaetodontidae	*Chaetodon*	*guttatissimus*	MGNW-05	NBE0354	IPCOM183-10	JF434782	JF457281	JF457949
Chaetodontidae	*Chaetodon*	*guttatissimus*	MGNW-05	NBE0355	IPCOM184-10	JF434781	JF457280	JF457948
Chaetodontidae	*Chaetodon*	*guttatissimus*	SWIO-02	REU0502	IPCOM355-10	JF434780	JF457279	JF457947
Chaetodontidae	*Chaetodon*	*guttatissimus*	SWIO-13	REU0759	IPCOM396-10	JF434779	JF457278	JF457946
Chaetodontidae	*Chaetodon*	*guttatissimus*	SWIO-32	REU102-1	IPCOM448-10	JF434778	JF457277	JF457945
Chaetodontidae	*Chaetodon*	*guttatissimus*	SWIO-34	REU213-1	IPCOM494-10	JF434777	JF457276	JF457944
Chaetodontidae	*Chaetodon*	*kleinii*	MGNW-05	NBE0359	IPCOM186-10	JF434785	JF457284	JF457952
Chaetodontidae	*Chaetodon*	*kleinii*	SWIO-13	REU0758	IPCOM395-10	JF434784	JF457283	JF457951
Chaetodontidae	*Chaetodon*	*kleinii*	SWIO-16	REU0940	IPCOM432-10	JF434786	JF457285	JF457953
Chaetodontidae	*Chaetodon*	*kleinii*	SWIO-32	REU104-1	IPCOM451-10	JF434783	JF457282	JF457950
Chaetodontidae	*Chaetodon*	*lunula*	MGNW-05	NBE0347	IPCOM178-10	JF434792	JF457291	JF457959
Chaetodontidae	*Chaetodon*	*lunula*	MGNW-10	NBE0654	IPCOM256-10	JF434791	JF457290	JF457958
Chaetodontidae	*Chaetodon*	*lunula*	SWIO-02	REU0501	IPCOM354-10	JF434790	JF457289	JF457957
Chaetodontidae	*Chaetodon*	*lunula*	SWIO-13	REU0721	IPCOM374-10	JF434789	JF457288	JF457956
Chaetodontidae	*Chaetodon*	*lunula*	SWIO-23	REU1611	IPCOM461-10	JF434788	JF457287	JF457955
Chaetodontidae	*Chaetodon*	*lunula*	SWIO-34	REU209-1	IPCOM491-10	JF434787	JF457286	JF457954
Chaetodontidae	*Chaetodon*	*lunulatus*	MBIO-05	MBIO0519	IPCOM025-10	JF434794	JF457293	JF457961
Chaetodontidae	*Chaetodon*	*lunulatus*	MBIO-05	MBIO0520	IPCOM026-10	JF434793	JF457292	JF457960
Chaetodontidae	*Chaetodon*	*melannotus*	MGNW-04	NBE0212	IPCOM155-10	JF434800	JF457299	JF457967
Chaetodontidae	*Chaetodon*	*melannotus*	MGNW-05	NBE0352	IPCOM182-10	JF434799	JF457298	JF457966
Chaetodontidae	*Chaetodon*	*melannotus*	SWIO-02	REU0113	IPCOM341-10	JF434798	JF457297	JF457965
Chaetodontidae	*Chaetodon*	*melannotus*	SWIO-02	REU0114	IPCOM342-10	JF434797	JF457296	JF457964
Chaetodontidae	*Chaetodon*	*melannotus*	SWIO-06	REU0294	IPCOM353-10	JF434796	JF457295	JF457963
Chaetodontidae	*Chaetodon*	*melannotus*	SWIO-34	REU211-1	IPCOM492-10	JF434795	JF457294	JF457962
Chaetodontidae	*Chaetodon*	*mertensii*	MBIO-06	MBIO0663	IPCOM038-10	JF434802	JF457301	JF457969
Chaetodontidae	*Chaetodon*	*mertensii*	MBIO-06	MBIO0664	IPCOM039-10	JF434801	JF457300	JF457968
Chaetodontidae	*Chaetodon*	*meyeri*	MGNW-05	NBE0350	IPCOM181-10	JF434804	JF457303	JF457971
Chaetodontidae	*Chaetodon*	*meyeri*	SWIO-13	REU0790	IPCOM410-10	JF434806	JF457305	JF457973
Chaetodontidae	*Chaetodon*	*meyeri*	SWIO-15	REU0904	IPCOM415-10	JF434805	JF457304	JF457972
Chaetodontidae	*Chaetodon*	*meyeri*	SWIO-23	REU1614	IPCOM463-10	JF434803	JF457302	JF457970
Chaetodontidae	*Chaetodon*	*ornatissimus*	MBIO-04	MBIO0257	IPCOM017-10	JF434807	JF457306	JF457974
Chaetodontidae	*Chaetodon*	*pelewensis*	MBIO-01	MBIO0084	IPCOM007-10	JF434809	JF457308	JF457976
Chaetodontidae	*Chaetodon*	*pelewensis*	MBIO-01	MBIO0085	IPCOM008-10	JF434808	JF457307	JF457975
Chaetodontidae	*Chaetodon*	*quadrimaculatus*	MBIO-11	MBIO0940	IPCOM055-10	JF434811	JF457310	JF457978
Chaetodontidae	*Chaetodon*	*quadrimaculatus*	MBIO-11	MBIO0941	IPCOM056-10	JF434810	JF457309	JF457977
Chaetodontidae	*Chaetodon*	*reticulatus*	MBIO-01	MBIO0049	IPCOM006-10	JF434813	JF457312	JF457980
Chaetodontidae	*Chaetodon*	*reticulatus*	MBIO-07	MBIO0847	IPCOM048-10	JF434812	JF457311	JF457979
Chaetodontidae	*Chaetodon*	*trichrous*	MBIO-06	MBIO0683	IPCOM041-10	JF434815	JF457314	JF457982
Chaetodontidae	*Chaetodon*	*trichrous*	MBIO-10	MBIO0898	IPCOM050-10	JF434814	JF457313	JF457981
Chaetodontidae	*Chaetodon*	*trifascialis*	MGNW-10	NBE0643	IPCOM249-10	JF434822	JF457321	JF457989
Chaetodontidae	*Chaetodon*	*trifascialis*	MGNW-10	NBE0644	IPCOM250-10	JF434821	JF457320	JF457988
Chaetodontidae	*Chaetodon*	*trifascialis*	MGNW-19	NBE1292	IPCOM315-10	JF434820	JF457319	JF457987
Chaetodontidae	*Chaetodon*	*trifascialis*	SWIO-13	REU0745	IPCOM385-10	JF434819	JF457318	JF457986
Chaetodontidae	*Chaetodon*	*trifascialis*	SWIO-13	REU0746	IPCOM386-10	JF434818	JF457317	JF457985
Chaetodontidae	*Chaetodon*	*trifascialis*	SWIO-32	REU103-1	IPCOM450-10	JF434817	JF457316	JF457984
Chaetodontidae	*Chaetodon*	*trifascialis*	SWIO-33	REU186-1	IPCOM485-10	JF434816	JF457315	JF457983
Chaetodontidae	*Chaetodon*	*trifasciatus*	MGNW-01	NBE0045	IPCOM113-10	JF434830	JF457329	JF457997
Chaetodontidae	*Chaetodon*	*trifasciatus*	MGNW-01	NBE0046	IPCOM114-10	JF434829	JF457328	JF457996
Chaetodontidae	*Chaetodon*	*trifasciatus*	MGNW-10	NBE0573	IPCOM225-10	JF434827	JF457326	JF457994
Chaetodontidae	*Chaetodon*	*trifasciatus*	MGNW-12	NBE1010	IPCOM263-10	JF434826	JF457325	JF457993
Chaetodontidae	*Chaetodon*	*trifasciatus*	SWIO-13	REU0718	IPCOM371-10	JF434825	JF457324	JF457992
Chaetodontidae	*Chaetodon*	*trifasciatus*	SWIO-13	REU0719	IPCOM372-10	JF434824	JF457323	JF457991
Chaetodontidae	*Chaetodon*	*trifasciatus*	SWIO-13	REU0720	IPCOM373-10	JF434823	JF457322	JF457990
Chaetodontidae	*Chaetodon*	*trifasciatus*	SWIO-16	REU0942	IPCOM433-10	JF434828	JF457327	JF457995
Chaetodontidae	*Chaetodon*	*ulietiensis*	MBIO-19	MBIO1286	IPCOM078-10	JF434832	JF457331	JF457999
Chaetodontidae	*Chaetodon*	*ulietiensis*	MBIO-20	MBIO1295	IPCOM081-10	JF434831	JF457330	JF457998
Chaetodontidae	*Chaetodon*	*unimaculatus*	MBIO-05	MBIO0522	IPCOM027-10	JF434836	JF457335	JF458003
Chaetodontidae	*Chaetodon*	*unimaculatus*	SWIO-13	REU0760	IPCOM397-10	JF434835	JF457334	JF458002
Chaetodontidae	*Chaetodon*	*unimaculatus*	SWIO-33	REU185-1	IPCOM484-10	JF434834	JF457333	JF458001
Chaetodontidae	*Chaetodon*	*unimaculatus**	SWIO-41	REU2631	IPCOM499-10	JF434833	JF457332	JF458000
Chaetodontidae	*Chaetodon*	*vagabundus*	MGNW-05	NBE0348	IPCOM179-10	JF434842	JF457341	JF458009
Chaetodontidae	*Chaetodon*	*vagabundus*	MGNW-05	NBE0349	IPCOM180-10	JF434841	JF457340	JF458008
Chaetodontidae	*Chaetodon*	*vagabundus*	SWIO-13	REU0772	IPCOM403-10	JF434840	JF457339	JF458007
Chaetodontidae	*Chaetodon*	*vagabundus*	SWIO-32	REU153-1	IPCOM459-10	JF434839	JF457338	JF458006
Chaetodontidae	*Chaetodon*	*vagabundus*	SWIO-34	REU212-1	IPCOM493-10	JF434838	JF457337	JF458005
Chaetodontidae	*Chaetodon*	*vagabundus*	SWIO-34	REU277-1	IPCOM507-10	JF434837	JF457336	JF458004
Chaetodontidae	*Chaetodon*	*xanthurus*	SWIO-13	REU0748	IPCOM387-10	JF434845	JF457344	JF458012
Chaetodontidae	*Chaetodon*	*xanthurus*	SWIO-13	REU0749	IPCOM388-10	JF434844	JF457343	JF458011
Chaetodontidae	*Chaetodon*	*xanthurus*	SWIO-23	REU1613	IPCOM462-10	JF434843	JF457342	JF458010
Chaetodontidae	*Chaetodon*	*zanzibariensis*	MGNW-39	BMADA0046	IPCOM001-10	JF434846	JF457345	JF458013
Labridae	*Cheilinus*	*chlorourus*	MBIO-14	MBIO1189	IPCOM066-10	JF434849	JF457348	-
Labridae	*Cheilinus*	*chlorourus*	MBIO-14	MBIO1190	IPCOM067-10	JF434848	JF457347	-
Labridae	*Cheilinus*	*chlorourus*	SWIO-32	REU146-2	IPCOM456-10	JF434847	JF457346	JF458014
Labridae	*Cheilinus*	*fasciatus**	MGNW-03	NBE0203	IPCOM152-10	JF434856	JF457355	JF458021
Labridae	*Cheilinus*	*fasciatus*	MGNW-08	NBE0478	IPCOM221-10	JF434855	JF457354	JF458020
Labridae	*Cheilinus*	*fasciatus*	MGNW-08	NBE0479	IPCOM222-10	JF434854	JF457353	JF458019
Labridae	*Cheilinus*	*fasciatus*	MGNW-08	NBE0480	IPCOM223-10	JF434853	JF457352	JF458018
Labridae	*Cheilinus*	*fasciatus*	MGNW-10	NBE0625	IPCOM238-10	JF434852	JF457351	JF458017
Labridae	*Cheilinus*	*fasciatus*	MGNW-10	NBE0626	IPCOM239-10	JF434851	JF457350	JF458016
Labridae	*Cheilinus*	*fasciatus*	MGNW-12	NBE1017	IPCOM268-10	JF434850	JF457349	JF458015
Labridae	*Cheilinus*	*oxycephalus*	MGNW-01	NBE0049	IPCOM117-10	JF434858	JF457357	JF458023
Labridae	*Cheilinus*	*oxycephalus*	MGNW-01	NBE0050	IPCOM118-10	JF434857	JF457356	JF458022
Labridae	*Cheilinus*	*trilobatus*	SWIO-13	REU0736	IPCOM378-10	JF434860	JF457359	JF458025
Labridae	*Cheilinus*	*trilobatus*	SWIO-32	REU146-1	IPCOM455-10	JF434859	JF457358	JF458024
Labridae	*Cheilio*	*inermis**	MGNW-01	NBE0048	IPCOM116-10	JF434863	JF457362	JF458028
Labridae	*Cheilio*	*inermis*	MGNW-23	NBE1424	IPCOM322-10	JF434862	JF457361	JF458027
Labridae	*Cheilio*	*inermis*	MGNW-23	NBE1425	IPCOM323-10	JF434861	JF457360	JF458026
Pomacentridae	*Chromis*	*acares*	MBIO-01	MBIO0025	IPCOM002-10	JF434865	JF457364	JF458030
Pomacentridae	*Chromis*	*acares*	MBIO-01	MBIO0026	IPCOM003-10	JF434864	JF457363	JF458029
Pomacentridae	*Chromis*	*agilis*	MBIO-15	MBIO1224	IPCOM070-10	JF434866	JF457365	-
Pomacentridae	*Chromis*	*alpha*	MBIO-11	MBIO0946	IPCOM058-10	JF434868	-	-
Pomacentridae	*Chromis*	*alpha*	MBIO-11	MBIO0947	IPCOM059-10	JF434867	-	-
Pomacentridae	*Chromis*	*atripectoralis*	MGNW-01	NBE0023	IPCOM096-10	JF434872	JF457369	JF458034
Pomacentridae	*Chromis*	*atripectoralis*	MGNW-01	NBE0024	IPCOM097-10	JF434871	JF457368	JF458033
Pomacentridae	*Chromis*	*atripectoralis**	MGNW-12	NBE1019	IPCOM270-10	JF434870	JF457367	JF458032
Pomacentridae	*Chromis*	*atripectoralis*	MGNW-15	NBE1076	IPCOM278-10	JF434869	JF457366	JF458031
Pomacentridae	*Chromis*	*chrysura*	SWIO-13	REU0710	IPCOM363-10	JF434876	JF457373	JF458038
Pomacentridae	*Chromis*	*chrysura*	SWIO-13	REU0711	IPCOM364-10	JF434875	JF457372	JF458037
Pomacentridae	*Chromis*	*chrysura*	SWIO-13	REU0712	IPCOM365-10	JF434874	JF457371	JF458036
Pomacentridae	*Chromis*	*chrysura*	SWIO-16	REU0986	IPCOM439-10	JF434873	JF457370	JF458035
Pomacentridae	*Chromis*	*dimidiata*	MBIO-13	MBIO1128	IPCOM064-10	JF434886	JF457383	JF458048
Pomacentridae	*Chromis*	*dimidiata*	MBIO-13	MBIO1129	IPCOM065-10	JF434885	JF457382	JF458047
Pomacentridae	*Chromis*	*dimidiata*	MGNW-01	NBE0056	IPCOM121-10	JF434884	JF457381	JF458046
Pomacentridae	*Chromis*	*dimidiata*	MGNW-06	NBE0444	IPCOM213-10	JF434882	JF457379	JF458044
Pomacentridae	*Chromis*	*dimidiata*	MGNW-06	NBE0447	IPCOM214-10	JF434881	JF457378	JF458043
Pomacentridae	*Chromis*	*dimidiata*	MGNW-06	NBE0448	IPCOM215-10	JF434880	JF457377	JF458042
Pomacentridae	*Chromis*	*dimidiata**	SWIO-13	REU0706	IPCOM359-10	JF434879	JF457376	JF458041
Pomacentridae	*Chromis*	*dimidiata*	SWIO-13	REU0707	IPCOM360-10	JF434878	JF457375	JF458040
Pomacentridae	*Chromis*	*dimidiata*	SWIO-13	REU0708	IPCOM361-10	JF434877	JF457374	JF458039
Pomacentridae	*Chromis*	*dimidiata*	SWIO-15	REU0927	IPCOM425-10	JF434883	JF457380	JF458045
Pomacentridae	*Chromis*	*iomelas*	MBIO-01	MBIO0095	IPCOM010-10	JF434887	JF457384	JF458049
Pomacentridae	*Chromis*	*nigrura*	SWIO-16	REU0996	IPCOM442-10	JF434889	JF457386	JF458051
Pomacentridae	*Chromis*	*nigrura*	SWIO-16	REU0997	IPCOM443-10	JF434888	JF457385	JF458050
Pomacentridae	*Chromis*	*opercularis*	MGNW-06	NBE0430	IPCOM206-10	JF434891	JF457388	JF458053
Pomacentridae	*Chromis*	*opercularis*	MGNW-06	NBE0431	IPCOM207-10	JF434890	JF457387	JF458052
Pomacentridae	*Chromis*	*ternatensis*	MGNW-01	NBE0097	IPCOM136-10	JF434897	-	JF458059
Pomacentridae	*Chromis*	*ternatensis*	MGNW-01	NBE0098	IPCOM137-10	JF434896	-	JF458058
Pomacentridae	*Chromis*	*ternatensis*	MGNW-06	NBE0440	IPCOM210-10	JF434895	-	JF458057
Pomacentridae	*Chromis*	*ternatensis*	MGNW-06	NBE0441	IPCOM211-10	JF434894	-	JF458056
Pomacentridae	*Chromis*	*ternatensis*	MGNW-06	NBE0442	IPCOM212-10	JF434893	-	JF458055
Pomacentridae	*Chromis*	*ternatensis*	MGNW-10	NBE0649	IPCOM254-10	JF434892	-	JF458054
Pomacentridae	*Chromis*	*vanderbilti*	MBIO-04	MBIO0280	IPCOM020-10	JF434899	JF457390	JF458061
Pomacentridae	*Chromis*	*vanderbilti*	MBIO-04	MBIO0281	IPCOM021-10	JF434898	JF457389	JF458060
Pomacentridae	*Chromis*	*viridis*	MGNW-15	NBE1072	IPCOM274-10	JF434906	JF457397	JF458068
Pomacentridae	*Chromis*	*viridis*	MGNW-15	NBE1073	IPCOM275-10	JF434905	JF457396	JF458067
Pomacentridae	*Chromis*	*viridis*	MGNW-15	NBE1074	IPCOM276-10	JF434904	JF457395	JF458066
Pomacentridae	*Chromis*	*viridis*	MGNW-15	NBE1075	IPCOM277-10	JF434903	JF457394	JF458065
Pomacentridae	*Chromis*	*viridis*	SWIO-02	REU0118	IPCOM343-10	JF434902	JF457393	JF458064
Pomacentridae	*Chromis*	*viridis*	SWIO-29	REU077-1	IPCOM402-10	JF434901	JF457392	JF458063
Pomacentridae	*Chromis*	*viridis*	SWIO-34	REU269-1	IPCOM501-10	JF434900	JF457391	JF458062
Pomacentridae	*Chromis*	*weberi*	MGNW-17	NBE1238	IPCOM299-10	JF434908	-	JF458070
Pomacentridae	*Chromis*	*weberi*	MGNW-17	NBE1239	IPCOM300-10	JF434907	-	JF458069
Pomacentridae	*Chromis*	*xanthochira*	MGNW-01	NBE0105	IPCOM138-10	JF434914	JF457403	JF458076
Pomacentridae	*Chromis*	*xanthochira*	MGNW-01	NBE0112	IPCOM139-10	JF434913	JF457402	JF458075
Pomacentridae	*Chromis*	*xanthochira*	MGNW-03	NBE0196	IPCOM148-10	JF434912	JF457401	JF458074
Pomacentridae	*Chromis*	*xanthochira*	MGNW-06	NBE0439	IPCOM209-10	JF434911	JF457400	JF458073
Pomacentridae	*Chromis*	*xanthochira*	MGNW-10	NBE0595	IPCOM227-10	JF434910	JF457399	JF458072
Pomacentridae	*Chromis*	*xanthochira**	SWIO-13	REU0709	IPCOM362-10	JF434909	JF457398	JF458071
Pomacentridae	*Chromis*	*xanthura*	MBIO-11	MBIO0945	IPCOM057-10	JF434916	JF457405	JF458078
Pomacentridae	*Chromis*	*xanthura*	MBIO-17	MBIO1264	IPCOM074-10	JF434915	JF457404	JF458077
Pomacentridae	*Chrysiptera*	*annulata*	MGNW-10	NBE0633	IPCOM240-10	JF434918	JF457407	JF458080
Pomacentridae	*Chrysiptera*	*annulata*	MGNW-10	NBE0634	IPCOM241-10	JF434917	JF457406	JF458079
Pomacentridae	*Chrysiptera*	*brownriggii*	MBIO-05	MBIO0493	IPCOM022-10	JF434930	JF457419	-
Pomacentridae	*Chrysiptera*	*brownriggii*	MBIO-05	MBIO0494	IPCOM023-10	JF434929	JF457418	-
Pomacentridae	*Chrysiptera*	*brownriggii**	MGNW-01	NBE0032	IPCOM102-10	JF434928	JF457417	JF458090
Pomacentridae	*Chrysiptera*	*brownriggii*	MGNW-01	NBE0035	IPCOM105-10	JF434927	JF457416	JF458089
Pomacentridae	*Chrysiptera*	*brownriggii*	MGNW-04	NBE0296	IPCOM175-10	JF434926	JF457415	JF458088
Pomacentridae	*Chrysiptera*	*brownriggii*	MGNW-04	NBE0297	IPCOM176-10	JF434925	JF457414	JF458087
Pomacentridae	*Chrysiptera*	*brownriggii*	MGNW-04	NBE0298	IPCOM177-10	JF434924	JF457413	JF458086
Pomacentridae	*Chrysiptera*	*brownriggii*	MGNW-10	NBE0650	IPCOM255-10	JF434923	JF457412	JF458085
Pomacentridae	*Chrysiptera*	*brownriggii*	SWIO-05	REU0136	IPCOM345-10	JF434922	JF457411	JF458084
Pomacentridae	*Chrysiptera*	*brownriggii*	SWIO-05	REU0137	IPCOM346-10	JF434921	JF457410	JF458083
Pomacentridae	*Chrysiptera*	*brownriggii*	SWIO-05	REU0138	IPCOM347-10	JF434920	JF457409	JF458082
Pomacentridae	*Chrysiptera*	*brownriggii*	SWIO-05	REU0139	IPCOM348-10	JF434919	JF457408	JF458081
Pomacentridae	*Chrysiptera*	*glauca*	SWIO-29	REU003-1	IPCOM333-10	JF434934	JF457423	JF458094
Pomacentridae	*Chrysiptera*	*glauca*	SWIO-29	REU003-2	IPCOM334-10	JF434933	JF457422	JF458093
Pomacentridae	*Chrysiptera*	*glauca*	SWIO-33	REU197-1	IPCOM488-10	JF434932	JF457421	JF458092
Pomacentridae	*Chrysiptera*	*glauca*	SWIO-33	REU197-2	IPCOM489-10	JF434931	JF457420	JF458091
Pomacentridae	*Chrysiptera*	*unimaculata*	SWIO-29	REU1659	IPCOM469-10	JF435153	JF457637	-
Labridae	*Cirrhilabrus*	*exquisitus*	MBIO-12	MBIO0991	IPCOM063-10	JF434935	JF457424	-
Labridae	*Cirrhilabrus*	*scottorum*	MBIO-04	MBIO0275	IPCOM018-10	JF434937	JF457426	JF458096
Labridae	*Cirrhilabrus*	*scottorum*	MBIO-04	MBIO0276	IPCOM019-10	JF434936	JF457425	JF458095
Labridae	*Coris*	*aygula**	MGNW-05	NBE0362	IPCOM188-10	JF434939	JF457428	JF458098
Labridae	*Coris*	*aygula*	SWIO-13	REU0765	IPCOM399-10	JF434938	JF457427	JF458097
Labridae	*Coris*	*caudimacula*	MGNW-05	NBE0366	IPCOM192-10	JF434943	JF457432	JF458102
Labridae	*Coris*	*caudimacula*	MGNW-05	NBE0367	IPCOM193-10	JF434942	JF457431	JF458101
Labridae	*Coris*	*caudimacula*	MGNW-19	NBE1285	IPCOM311-10	JF434941	JF457430	JF458100
Labridae	*Coris*	*caudimacula*	SWIO-13	REU0784	IPCOM405-10	JF434940	JF457429	JF458099
Labridae	*Coris*	*cuvieri**	MGNW-05	NBE0379	IPCOM198-10	JF434947	JF457436	JF458106
Labridae	*Coris*	*cuvieri*	MGNW-05	NBE0380	IPCOM199-10	JF434946	JF457435	JF458105
Labridae	*Coris*	*cuvieri*	MGNW-05	NBE0381	IPCOM200-10	JF434945	JF457434	JF458104
Labridae	*Coris*	*cuvieri*	SWIO-13	REU0770	IPCOM401-10	JF434944	JF457433	JF458103
Labridae	*Coris*	*gaimard*	MBIO-06	MBIO0665	IPCOM040-10	JF434948	JF457437	JF458107
Pomacentridae	*Dascyllus*	*aruanus*	MGNW-03	NBE0187	IPCOM144-10	JF434955	JF457444	JF458114
Pomacentridae	*Dascyllus*	*aruanus*	MGNW-03	NBE0188	IPCOM145-10	JF434954	JF457443	JF458113
Pomacentridae	*Dascyllus*	*aruanus*	MGNW-03	NBE0189	IPCOM146-10	JF434953	JF457442	JF458112
Pomacentridae	*Dascyllus*	*aruanus**	SWIO-29	REU004-1	IPCOM335-10	JF434952	JF457441	JF458111
Pomacentridae	*Dascyllus*	*aruanus*	SWIO-29	REU004-2	IPCOM336-10	JF434951	JF457440	JF458110
Pomacentridae	*Dascyllus*	*aruanus*	SWIO-41	REU2764	IPCOM505-10	JF434950	JF457439	JF458109
Pomacentridae	*Dascyllus*	*aruanus*	SWIO-41	REU2765	IPCOM506-10	JF434949	JF457438	JF458108
Pomacentridae	*Dascyllus*	*carneus*	MGNW-03	NBE0199	IPCOM150-10	JF434959	JF457448	JF458118
Pomacentridae	*Dascyllus*	*carneus*	MGNW-10	NBE0597	IPCOM228-10	JF434958	JF457447	JF458117
Pomacentridae	*Dascyllus*	*carneus*	MGNW-10	NBE0598	IPCOM229-10	JF434957	JF457446	JF458116
Pomacentridae	*Dascyllus*	*carneus*	MGNW-10	NBE0599	IPCOM230-10	JF434956	JF457445	JF458115
Pomacentridae	*Dascyllus*	*flavicaudus*	MBIO-01	MBIO0045	IPCOM004-10	JF434961	JF457450	JF458120
Pomacentridae	*Dascyllus*	*flavicaudus*	MBIO-01	MBIO0046	IPCOM005-10	JF434960	JF457449	JF458119
Pomacentridae	*Dascyllus*	*trimaculatus*	SWIO-15	REU0931	IPCOM428-10	JF434964	-	JF458123
Pomacentridae	*Dascyllus*	*trimaculatus*	SWIO-15	REU0932	IPCOM429-10	JF434963	-	JF458122
Pomacentridae	*Dascyllus*	*trimaculatus*	SWIO-16	REU0988	IPCOM440-10	JF434962	-	JF458121
Labridae	*Epibulus*	*insidiator*	MGNW-01	NBE0027	IPCOM100-10	JF434969	JF457455	JF458128
Labridae	*Epibulus*	*insidiator*	MGNW-01	NBE0028	IPCOM101-10	JF434968	JF457454	JF458127
Labridae	*Epibulus*	*insidiator**	MGNW-01	NBE0076	IPCOM129-10	JF434967	JF457453	JF458126
Labridae	*Epibulus*	*insidiator*	MGNW-12	NBE1018	IPCOM269-10	JF434966	JF457452	JF458125
Labridae	*Epibulus*	*insidiator*	SWIO-41	REU2619	IPCOM495-10	JF434965	JF457451	JF458124
Chaetodontidae	*Forcipiger*	*flavissimus*	SWIO-13	REU0737	IPCOM379-10	JF434973	JF457459	-
Chaetodontidae	*Forcipiger*	*flavissimus**	SWIO-13	REU0738	IPCOM380-10	JF434972	JF457458	-
Chaetodontidae	*Forcipiger*	*flavissimus*	SWIO-13	REU0756	IPCOM394-10	JF434971	JF457457	-
Chaetodontidae	*Forcipiger*	*flavissimus*	SWIO-16	REU1695	IPCOM475-10	JF434970	JF457456	-
Chaetodontidae	*Forcipiger*	*longirostris*	MBIO-11	MBIO0927	IPCOM051-10	JF434975	JF457461	-
Chaetodontidae	*Forcipiger*	*longirostris*	MBIO-11	MBIO0928	IPCOM052-10	JF434974	JF457460	-
Labridae	*Gomphosus*	*caeruleus*	MGNW-01	NBE0043	IPCOM112-10	JF434979	JF457465	JF458133
Labridae	*Gomphosus*	*caeruleus*	SWIO-02	REU0110	IPCOM338-10	JF434978	JF457464	-
Labridae	*Gomphosus*	*caeruleus*	SWIO-13	REU0750	IPCOM389-10	JF434977	JF457463	-
Labridae	*Gomphosus*	*caeruleus*	SWIO-13	REU0751	IPCOM390-10	JF434976	JF457462	-
Labridae	*Gomphosus*	*varius*	MBIO-07	MBIO0784	IPCOM045-10	JF434981	JF457467	JF458135
Labridae	*Gomphosus*	*varius*	MBIO-07	MBIO0785	IPCOM046-10	JF434980	JF457466	JF458134
Labridae	*Halichoeres*	*cosmetus*	MGNW-05	NBE0363	IPCOM189-10	JF434984	JF457470	JF458138
Labridae	*Halichoeres*	*cosmetus*	MGNW-05	NBE0364	IPCOM190-10	JF434983	JF457469	JF458137
Labridae	*Halichoeres*	*cosmetus**	SWIO-13	REU0787	IPCOM408-10	JF434986	JF457472	JF458140
Labridae	*Halichoeres*	*cosmetus*	SWIO-13	REU0788	IPCOM409-10	JF434985	JF457471	JF458139
Labridae	*Halichoeres*	*cosmetus*	SWIO-16	REU1021	IPCOM447-10	JF434982	JF457468	JF458136
Labridae	*Halichoeres*	*hortulanus*	MGNW-01	NBE0047	IPCOM115-10	JF434990	JF457476	JF458144
Labridae	*Halichoeres*	*hortulanus*	MGNW-05	NBE0361	IPCOM187-10	JF434989	JF457475	JF458143
Labridae	*Halichoeres*	*hortulanus*	MGNW-10	NBE0647	IPCOM252-10	JF434988	JF457474	JF458142
Labridae	*Halichoeres*	*hortulanus*	SWIO-13	REU0767	IPCOM400-10	JF434987	JF457473	JF458141
Labridae	*Halichoeres*	*margitaceus*	MBIO-05	MBIO0588	IPCOM035-10	JF434992	JF457478	-
Labridae	*Halichoeres*	*margitaceus*	MBIO-05	MBIO0589	IPCOM036-10	JF434991	JF457477	-
Labridae	*Halichoeres*	*marginatus*	MGNW-10	NBE0648	IPCOM253-10	JF434994	JF457480	JF458146
Labridae	*Halichoeres*	*marginatus*	SWIO-13	REU0793	IPCOM413-10	JF434995	JF457481	JF458147
Labridae	*Halichoeres*	*marginatus*	SWIO-16	REU1023	IPCOM449-10	JF434993	JF457479	JF458145
Labridae	*Halichoeres*	*nebulosus*	MGNW-04	NBE0235	IPCOM157-10	JF434998	JF457484	-
Labridae	*Halichoeres*	*nebulosus*	MGNW-20	NBE1323	IPCOM320-10	JF434997	JF457483	-
Labridae	*Halichoeres*	*nebulosus*	MGNW-20	NBE1325	IPCOM321-10	JF434996	JF457482	-
Labridae	*Halichoeres*	*nigrescens*	MGNW-04	NBE0236	IPCOM158-10	JF435002	JF457488	-
Labridae	*Halichoeres*	*nigrescens*	MGNW-04	NBE0237	IPCOM159-10	JF435001	JF457487	-
Labridae	*Halichoeres*	*nigrescens*	MGNW-04	NBE0238	IPCOM160-10	JF435000	JF457486	-
Labridae	*Halichoeres*	*nigrescens*	MGNW-13	NBE1030	IPCOM271-10	JF434999	JF457485	-
Labridae	*Halichoeres*	*ornatissimus*	MBIO-19	MBIO1288	IPCOM079-10	JF435004	JF457490	-
Labridae	*Halichoeres*	*ornatissimus*	MBIO-24	MBIO1382	IPCOM086-10	JF435003	JF457489	-
Labridae	*Halichoeres*	*scapularis*	MGNW-19	NBE1266	IPCOM301-10	JF435010	JF457496	-
Labridae	*Halichoeres*	*scapularis*	MGNW-19	NBE1267	IPCOM302-10	JF435009	JF457495	-
Labridae	*Halichoeres*	*scapularis*	MGNW-19	NBE1268	IPCOM303-10	JF435008	JF457494	JF458148
Labridae	*Halichoeres*	*scapularis*	SWIO-29	REU013-1	IPCOM344-10	JF435007	JF457493	-
Labridae	*Halichoeres*	*scapularis*	SWIO-29	REU074-1	IPCOM381-10	JF435006	JF457492	-
Labridae	*Halichoeres*	*scapularis*	SWIO-29	REU074-2	IPCOM382-10	JF435005	JF457491	-
Labridae	*Halichoeres*	*trimaculatus*	MBIO-05	MBIO0561	IPCOM031-10	JF435012	JF457498	-
Labridae	*Halichoeres*	*trimaculatus*	MBIO-05	MBIO0562	IPCOM032-10	JF435011	JF457497	-
Labridae	*Hemigymnus*	*fasciatus**	MGNW-03	NBE0204	IPCOM153-10	JF435016	JF457502	JF458152
Labridae	*Hemigymnus*	*fasciatus*	MGNW-19	NBE1299	IPCOM316-10	JF435015	JF457501	JF458151
Labridae	*Hemigymnus*	*fasciatus*	SWIO-13	REU0743	IPCOM383-10	JF435014	JF457500	JF458150
Labridae	*Hemigymnus*	*fasciatus*	SWIO-13	REU0744	IPCOM384-10	JF435013	JF457499	JF458149
Labridae	*Hemigymnus*	*melanopterus*	MGNW-07	NBE0458	IPCOM219-10	JF435018	JF457504	JF458154
Labridae	*Hemigymnus*	*melanopterus*	MGNW-07	NBE0459	IPCOM220-10	JF435017	JF457503	JF458153
Chaetodontidae	*Hemitaurichthys*	*polylepis*	MBIO-11	MBIO0936	IPCOM053-10	JF435020	JF457506	JF458156
Chaetodontidae	*Hemitaurichthys*	*polylepis*	MBIO-11	MBIO0937	IPCOM054-10	JF435019	JF457505	JF458155
Chaetodontidae	*Hemitaurichthys*	*thompsoni*	MBIO-24	MBIO1378	IPCOM084-10	JF435022	JF457508	JF458158
Chaetodontidae	*Hemitaurichthys*	*thompsoni*	MBIO-24	MBIO1379	IPCOM085-10	JF435021	JF457507	JF458157
Chaetodontidae	*Hemitaurichthys*	*zoster**	SWIO-13	REU0791	IPCOM411-10	JF435024	JF457510	JF458160
Chaetodontidae	*Hemitaurichthys*	*zoster*	SWIO-13	REU0792	IPCOM412-10	JF435023	JF457509	JF458159
Chaetodontidae	*Heniochus*	*acuminatus*	SWIO-13	REU0730	IPCOM376-10	JF435025	JF457511	JF458161
Chaetodontidae	*Heniochus*	*chrysostomus*	MBIO-05	MBIO0526	IPCOM028-10	JF435027	JF457513	JF458163
Chaetodontidae	*Heniochus*	*chrysostomus*	MBIO-05	MBIO0527	IPCOM029-10	JF435026	JF457512	JF458162
Chaetodontidae	*Heniochus*	*diphreutes**	MGNW-01	NBE0041	IPCOM110-10	JF435031	JF457517	JF458167
Chaetodontidae	*Heniochus*	*diphreutes*	MGNW-03	NBE0180	IPCOM141-10	JF435030	JF457516	JF458166
Chaetodontidae	*Heniochus*	*diphreutes*	MGNW-10	NBE0616	IPCOM234-10	JF435029	JF457515	JF458165
Chaetodontidae	*Heniochus*	*diphreutes*	SWIO-13	REU0729	IPCOM375-10	JF435028	JF457514	JF458164
Chaetodontidae	*Heniochus*	*monoceros*	SWIO-15	REU0905	IPCOM416-10	JF435035	JF457521	JF458171
Chaetodontidae	*Heniochus*	*monoceros*	SWIO-15	REU0906	IPCOM417-10	JF435034	JF457520	JF458170
Chaetodontidae	*Heniochus*	*monoceros*	SWIO-32	REU152-1	IPCOM458-10	JF435033	JF457519	JF458169
Chaetodontidae	*Heniochus*	*monoceros*	SWIO-34	REU276-1	IPCOM504-10	JF435032	JF457518	JF458168
Labridae	*Hologymnosus*	*annulatus*	MGNW-05	NBE0377	IPCOM197-10	JF435038	JF457524	JF458174
Labridae	*Hologymnosus*	*annulatus*	MGNW-19	NBE1309	IPCOM317-10	JF435037	JF457523	JF458173
Labridae	*Hologymnosus*	*annulatus*	MGNW-19	NBE1310	IPCOM318-10	JF435036	JF457522	JF458172
Labridae	*Labroides*	*bicolor*	MBIO-05	MBIO0592	IPCOM037-10	JF435041	JF457527	-
Labridae	*Labroides*	*bicolor*	MGNW-16	NBE1133	IPCOM290-10	JF435039	JF457525	-
Labridae	*Labroides*	*bicolor*	SWIO-13	REU0786	IPCOM407-10	JF435040	JF457526	-
Labridae	*Labroides*	*dimidiatus**	MGNW-04	NBE0226	IPCOM156-10	JF435043	JF457529	JF458176
Labridae	*Labroides*	*dimidiatus*	SWIO-23	REU1616	IPCOM464-10	JF435042	JF457528	JF458175
Labridae	*Labropsis*	*polynesica*	MBIO-15	MBIO1226	IPCOM071-10	JF435045	JF457531	-
Labridae	*Labropsis*	*polynesica*	MBIO-19	MBIO1765	IPCOM075-10	JF435044	JF457530	-
Labridae	*Macropharyngodon*	*bipartitus*	MGNW-16	NBE1122	IPCOM286-10	JF435048	JF457534	-
Labridae	*Macropharyngodon*	*bipartitus*	MGNW-16	NBE1123	IPCOM287-10	JF435047	JF457533	-
Labridae	*Macropharyngodon*	*bipartitus*	MGNW-16	NBE1124	IPCOM288-10	JF435046	JF457532	-
Labridae	*Macropharyngodon*	*bipartitus*	SWIO-15	REU0918	IPCOM418-10	JF435049	JF457535	JF457907
Labridae	*Macropharyngodon*	*meleagris*	MBIO-46	MBIO1847	IPCOM091-10	JF435051	JF457537	-
Labridae	*Macropharyngodon*	*meleagris*	MBIO-46	MBIO1848	IPCOM092-10	JF435050	JF457536	-
Pomacentridae	*Neoglyphidodon*	*melas*	MGNW-01	NBE0015	IPCOM094-10	JF435053	JF457539	JF458178
Pomacentridae	*Neoglyphidodon*	*melas*	MGNW-10	NBE0660	IPCOM257-10	JF435052	JF457538	JF458177
Pomacentridae	*Neopomacentrus*	*azysron*	MGNW-04	NBE0245	IPCOM165-10	JF435056	JF457542	JF458181
Pomacentridae	*Neopomacentrus*	*azysron*	MGNW-04	NBE0246	IPCOM166-10	JF435055	JF457541	JF458180
Pomacentridae	*Neopomacentrus*	*azysron*	MGNW-04	NBE0247	IPCOM167-10	JF435054	JF457540	JF458179
Pomacentridae	*Neopomacentrus*	*cyanomos*	MGNW-04	NBE0240	IPCOM162-10	JF435059	JF457545	JF458184
Pomacentridae	*Neopomacentrus*	*cyanomos*	MGNW-04	NBE0241	IPCOM163-10	JF435058	JF457544	JF458183
Pomacentridae	*Neopomacentrus*	*cyanomos*	MGNW-04	NBE0242	IPCOM164-10	JF435057	JF457543	JF458182
Labridae	*Novaculichthys*	*taeniourus*	MBIO-19	MBIO1282	IPCOM076-10	JF435061	JF457547	-
Labridae	*Novaculichthys*	*taeniourus*	MBIO-19	MBIO1283	IPCOM077-10	JF435060	JF457546	-
Labridae	*Oxycheilinus*	*arenatus*	MBIO-33	MBIO1614	IPCOM088-10	JF435062	JF457548	-
Labridae	*Oxycheilinus*	*digramma**	MGNW-01	NBE0026	IPCOM099-10	JF435066	JF457552	JF458188
Labridae	*Oxycheilinus*	*digramma*	MGNW-01	NBE0077	IPCOM130-10	JF435065	JF457551	JF458187
Labridae	*Oxycheilinus*	*digramma*	MGNW-03	NBE0202	IPCOM151-10	JF435064	JF457550	JF458186
Labridae	*Oxycheilinus*	*digramma*	MGNW-10	NBE0615	IPCOM233-10	JF435063	JF457549	JF458185
Labridae	*Oxycheilinus*	*unifasciatus*	MBIO-17	MBIO1257	IPCOM072-10	JF435068	JF457554	JF458190
Labridae	*Oxycheilinus*	*unifasciatus*	MBIO-23	MBIO1345	IPCOM083-10	JF435067	JF457553	JF458189
Pomacentridae	*Plectroglyphidodon*	*dickii**	SWIO-13	REU0715	IPCOM368-10	JF435069	JF457555	JF458191
Pomacentridae	*Plectroglyphidodon*	*dickii*	SWIO-15	REU0926	IPCOM424-10	JF435070	JF457556	JF458192
Pomacentridae	*Plectroglyphidodon*	*imparipennis*	SWIO-30	REU1685	IPCOM472-10	JF435074	JF457560	JF458196
Pomacentridae	*Plectroglyphidodon*	*imparipennis*	SWIO-30	REU1746	IPCOM477-10	JF435073	JF457559	JF458195
Pomacentridae	*Plectroglyphidodon*	*imparipennis*	SWIO-30	REU1747	IPCOM478-10	JF435072	JF457558	JF458194
Pomacentridae	*Plectroglyphidodon*	*imparipennis*	SWIO-30	REU1803	IPCOM481-10	JF435071	JF457557	JF458193
Pomacentridae	*Plectroglyphidodon*	*johnstonianus*	MBIO-01	MBIO0087	IPCOM009-10	JF435079	JF457565	-
Pomacentridae	*Plectroglyphidodon*	*johnstonianus*	SWIO-13	REU0716	IPCOM369-10	JF435078	JF457564	-
Pomacentridae	*Plectroglyphidodon*	*johnstonianus*	SWIO-13	REU0717	IPCOM370-10	JF435077	JF457563	-
Pomacentridae	*Plectroglyphidodon*	*johnstonianus*	SWIO-32	REU109-1	IPCOM452-10	JF435076	JF457562	-
Pomacentridae	*Plectroglyphidodon*	*johnstonianus*	SWIO-32	REU156-2	IPCOM460-10	JF435075	JF457561	-
Pomacentridae	*Plectroglyphidodon*	*lacrymatus**	MGNW-01	NBE0036	IPCOM106-10	JF435084	JF457570	JF458201
Pomacentridae	*Plectroglyphidodon*	*lacrymatus*	MGNW-01	NBE0037	IPCOM107-10	JF435083	JF457569	JF458200
Pomacentridae	*Plectroglyphidodon*	*lacrymatus*	MGNW-01	NBE0038	IPCOM108-10	JF435082	JF457568	JF458199
Pomacentridae	*Plectroglyphidodon*	*lacrymatus*	MGNW-01	NBE0039	IPCOM109-10	JF435081	JF457567	JF458198
Pomacentridae	*Plectroglyphidodon*	*lacrymatus*	MGNW-19	NBE1270	IPCOM305-10	JF435080	JF457566	JF458197
Pomacentridae	*Plectroglyphidodon*	*leucozonus*	MGNW-10	NBE0635	IPCOM242-10	JF435086	-	-
Pomacentridae	*Plectroglyphidodon*	*leucozonus*	MGNW-10	NBE0636	IPCOM243-10	JF435085	-	-
Pomacentridae	*Plectroglyphidodon*	*phoenixensis*	MBIO-36	MBIO1652	IPCOM090-10	JF435088	JF457572	-
Pomacentridae	*Plectroglyphidodon*	*phoenixensis*	SWIO-30	REU1745	IPCOM476-10	JF435087	JF457571	JF458202
Pomacentridae	*Plectroglyphidodon*	*randalli*	SWIO-05	REU0140	IPCOM349-10	JF435092	JF457576	JF458206
Pomacentridae	*Plectroglyphidodon*	*randalli*	SWIO-05	REU0141	IPCOM350-10	JF435091	JF457575	JF458205
Pomacentridae	*Plectroglyphidodon*	*randalli*	SWIO-05	REU0142	IPCOM351-10	JF435090	JF457574	JF458204
Pomacentridae	*Plectroglyphidodon*	*randalli*	SWIO-05	REU0143	IPCOM352-10	JF435089	JF457573	JF458203
Labridae	*Polylepion*	*russelli*	MBIO-50	MBIO1871	IPCOM093-10	JF435093	JF457577	-
Pomacentridae	*Pomacentrus*	*agassizi**	SWIO-13	REU0713	IPCOM366-10	JF435096	JF457580	JF458209
Pomacentridae	*Pomacentrus*	*agassizi*	SWIO-13	REU0714	IPCOM367-10	JF435095	JF457579	JF458208
Pomacentridae	*Pomacentrus*	*agassizi*	SWIO-15	REU0933	IPCOM430-10	JF435098	JF457582	JF458211
Pomacentridae	*Pomacentrus*	*agassizi*	SWIO-15	REU0934	IPCOM431-10	JF435097	JF457581	JF458210
Pomacentridae	*Pomacentrus*	*agassizi*	SWIO-16	REU0983	IPCOM438-10	JF435094	JF457578	JF458207
Pomacentridae	*Pomacentrus*	*arabicus*	MGNW-24	NBE1556	IPCOM325-10	JF435099	JF457583	JF458212
Pomacentridae	*Pomacentrus*	*baenschi**	MGNW-01	NBE0057	IPCOM122-10	JF435108	JF457592	JF458221
Pomacentridae	*Pomacentrus*	*baenschi*	MGNW-01	NBE0060	IPCOM123-10	JF435107	JF457591	JF458220
Pomacentridae	*Pomacentrus*	*baenschi*	MGNW-01	NBE0061	IPCOM124-10	JF435106	JF457590	JF458219
Pomacentridae	*Pomacentrus*	*baenschi*	MGNW-01	NBE0062	IPCOM125-10	JF435105	JF457589	JF458218
Pomacentridae	*Pomacentrus*	*baenschi*	MGNW-01	NBE0063	IPCOM126-10	JF435104	JF457588	JF458217
Pomacentridae	*Pomacentrus*	*baenschi*	MGNW-05	NBE0373	IPCOM195-10	JF435103	JF457587	JF458216
Pomacentridae	*Pomacentrus*	*baenschi*	MGNW-05	NBE0374	IPCOM196-10	JF435102	JF457586	JF458215
Pomacentridae	*Pomacentrus*	*baenschi*	MGNW-05	NBE1719	IPCOM326-10	JF435101	JF457585	JF458214
Pomacentridae	*Pomacentrus*	*baenschi*	MGNW-05	NBE1720	IPCOM327-10	JF435100	JF457584	JF458213
Pomacentridae	*Pomacentrus*	*caeruleus*	MGNW-01	NBE0055	IPCOM120-10	JF435115	JF457599	JF458228
Pomacentridae	*Pomacentrus*	*caeruleus*	MGNW-05	NBE0372	IPCOM194-10	JF435112	JF457596	JF458225
Pomacentridae	*Pomacentrus*	*caeruleus*	MGNW-05	NBE0388	IPCOM203-10	JF435111	JF457595	JF458224
Pomacentridae	*Pomacentrus*	*caeruleus*	MGNW-17	NBE1197	IPCOM296-10	JF435110	JF457594	JF458223
Pomacentridae	*Pomacentrus*	*caeruleus*	SWIO-13	REU0704	IPCOM358-10	JF435109	JF457593	JF458222
Pomacentridae	*Pomacentrus*	*caeruleus*	SWIO-15	REU0928	IPCOM426-10	JF435114	JF457598	JF458227
Pomacentridae	*Pomacentrus*	*caeruleus*	SWIO-15	REU0929	IPCOM427-10	JF435113	JF457597	JF458226
Pomacentridae	*Pomacentrus*	*pavo*	MBIO-05	MBIO0565	IPCOM033-10	JF435124	JF457608	-
Pomacentridae	*Pomacentrus*	*pavo*	MBIO-05	MBIO0566	IPCOM034-10	JF435123	JF457607	-
Pomacentridae	*Pomacentrus*	*pavo**	MGNW-04	NBE0256	IPCOM169-10	JF435122	JF457606	JF458235
Pomacentridae	*Pomacentrus*	*pavo*	MGNW-04	NBE0257	IPCOM170-10	JF435121	JF457605	JF458234
Pomacentridae	*Pomacentrus*	*pavo*	MGNW-04	NBE0258	IPCOM171-10	JF435120	JF457604	JF458233
Pomacentridae	*Pomacentrus*	*pavo*	MGNW-04	NBE0259	IPCOM172-10	JF435119	JF457603	JF458232
Pomacentridae	*Pomacentrus*	*pavo*	MGNW-10	NBE0622	IPCOM235-10	JF435118	JF457602	JF458231
Pomacentridae	*Pomacentrus*	*pavo*	MGNW-10	NBE0623	IPCOM236-10	JF435117	JF457601	JF458230
Pomacentridae	*Pomacentrus*	*pavo*	MGNW-10	NBE0624	IPCOM237-10	JF435116	JF457600	JF458229
Pomacentridae	*Pomacentrus*	*sulfureus*	MGNW-01	NBE0092	IPCOM133-10	JF435128	JF457612	JF458239
Pomacentridae	*Pomacentrus*	*sulfureus*	MGNW-01	NBE0093	IPCOM134-10	JF435127	JF457611	JF458238
Pomacentridae	*Pomacentrus*	*sulfureus*	MGNW-01	NBE0094	IPCOM135-10	JF435126	JF457610	JF458237
Pomacentridae	*Pomacentrus*	*sulfureus*	MGNW-10	NBE0594	IPCOM226-10	JF435125	JF457609	JF458236
Pomacentridae	*Pomacentrus*	*trichrous*	MGNW-01	NBE0089	IPCOM131-10	JF435130	JF457614	JF458241
Pomacentridae	*Pomacentrus*	*trichrous*	MGNW-01	NBE0090	IPCOM132-10	JF435129	JF457613	JF458240
Pomacentridae	*Pomacentrus*	*trilineatus*	MGNW-04	NBE0264	IPCOM173-10	JF435136	JF457620	JF458247
Pomacentridae	*Pomacentrus*	*trilineatus*	MGNW-04	NBE0265	IPCOM174-10	JF435135	JF457619	JF458246
Pomacentridae	*Pomacentrus*	*trilineatus*	MGNW-05	NBE0383	IPCOM202-10	JF435134	JF457618	JF458245
Pomacentridae	*Pomacentrus*	*trilineatus*	MGNW-08	NBE0497	IPCOM224-10	JF435133	JF457617	JF458244
Pomacentridae	*Pomacentrus*	*trilineatus*	MGNW-12	NBE1015	IPCOM267-10	JF435132	JF457616	JF458243
Pomacentridae	*Pomacentrus*	*trilineatus*	MGNW-19	NBE1271	IPCOM306-10	JF435131	JF457615	JF458242
Pomacentridae	*Pomachromis*	*fuscidorsalis*	MBIO-12	MBIO0957	IPCOM060-10	JF435138	JF457622	-
Pomacentridae	*Pomachromis*	*fuscidorsalis*	MBIO-12	MBIO0958	IPCOM061-10	JF435137	JF457621	-
Labridae	*Pseudocheilinus*	*evadinus*	MBIO-05	MBIO0560	IPCOM030-10	JF435139	JF457623	-
Labridae	*Pseudocheilinus*	*hexataenia*	MGNW-06	NBE0412	IPCOM204-10	JF435141	JF457625	-
Labridae	*Pseudocheilinus*	*hexataenia*	MGNW-06	NBE0414	IPCOM205-10	JF435140	JF457624	-
Labridae	*Pseudocheilinus*	*ocellatus*	MBIO-12	MBIO0989	IPCOM062-10	JF435142	JF457626	-
Labridae	*Pseudocheilinus*	*octotaenia*	SWIO-13	REU0785	IPCOM406-10	JF435144	JF457628	-
Labridae	*Pseudocheilinus*	*octotaenia*	SWIO-15	REU0920	IPCOM420-10	JF435145	JF457629	-
Labridae	*Pseudocheilinus*	*octotaenia*	SWIO-16	REU1015	IPCOM446-10	JF435143	JF457627	-
Labridae	*Pseudocheilinus*	*tetrataenia*	MBIO-01	MBIO0106	IPCOM011-10	JF435147	JF457631	-
Labridae	*Pseudocheilinus*	*tetrataenia*	MBIO-01	MBIO0107	IPCOM012-10	JF435146	JF457630	-
Labridae	*Pseudocoris*	*aurantiofasciata*	MBIO-35	MBIO1626	IPCOM089-10	JF435148	JF457632	-
Labridae	*Pseudodax*	*moluccanus**	MGNW-05	NBE0382	IPCOM201-10	JF435149	JF457633	JF458248
Labridae	*Pseudojuloides*	*atavai*	MBIO-19	MBIO1289	IPCOM080-10	JF435151	JF457635	-
Labridae	*Pseudojuloides*	*atavai*	MBIO-31	MBIO1549	IPCOM087-10	JF435150	JF457634	-
Pomacentridae	*Stegastes*	*albifasciatus*	MBIO-07	MBIO0732	IPCOM042-10	JF435152	JF457636	-
Pomacentridae	*Stegastes*	*limbatus**	SWIO-29	REU002-1	IPCOM331-10	JF435160	JF457644	JF458255
Pomacentridae	*Stegastes*	*limbatus*	SWIO-29	REU002-2	IPCOM332-10	JF435159	JF457643	JF458254
Pomacentridae	*Stegastes*	*limbatus*	SWIO-29	REU1654	IPCOM468-10	JF435158	JF457642	JF458253
Pomacentridae	*Stegastes*	*limbatus*	SWIO-41	REU2774	IPCOM508-10	JF435157	JF457641	JF458252
Pomacentridae	*Stegastes*	*limbatus*	SWIO-41	REU2775	IPCOM509-10	JF435156	JF457640	JF458251
Pomacentridae	*Stegastes*	*limbatus*	SWIO-41	REU2776	IPCOM510-10	JF435155	JF457639	JF458250
Pomacentridae	*Stegastes*	*limbatus*	SWIO-41	REU2777	IPCOM511-10	JF435154	JF457638	JF458249
Pomacentridae	*Stegastes*	*lividus*	SWIO-29	REU001-1	IPCOM329-10	JF435164	JF457648	JF458259
Pomacentridae	*Stegastes*	*lividus*	SWIO-29	REU001-2	IPCOM330-10	JF435163	JF457647	JF458258
Pomacentridae	*Stegastes*	*lividus*	SWIO-41	REU2754	IPCOM502-10	JF435162	JF457646	JF458257
Pomacentridae	*Stegastes*	*lividus*	SWIO-41	REU2755	IPCOM503-10	JF435161	JF457645	JF458256
Pomacentridae	*Stegastes*	*nigricans*	MGNW-15	NBE1085	IPCOM279-10	JF435170	JF457654	JF458265
Pomacentridae	*Stegastes*	*nigricans*	MGNW-19	NBE1269	IPCOM304-10	JF435169	JF457653	JF458264
Pomacentridae	*Stegastes*	*nigricans*	SWIO-29	REU005-1	IPCOM337-10	JF435168	JF457652	JF458263
Pomacentridae	*Stegastes*	*nigricans*	SWIO-32	REU111-1	IPCOM453-10	JF435167	JF457651	JF458262
Pomacentridae	*Stegastes*	*nigricans*	SWIO-41	REU2661	IPCOM500-10	JF435166	JF457650	JF458261
Pomacentridae	*Stegastes*	*nigricans*	SWIO-41	REU2784	IPCOM512-10	JF435165	JF457649	JF458260
Pomacentridae	*Stegastes*	*pelicieri*	SWIO-13	REU0703	IPCOM357-10	JF435173	JF457657	JF458268
Pomacentridae	*Stegastes*	*pelicieri*	SWIO-16	REU1909	IPCOM486-10	JF435172	JF457656	JF458267
Pomacentridae	*Stegastes*	*pelicieri*	SWIO-34	REU207-1	IPCOM490-10	JF435171	JF457655	JF458266
Labridae	*Stethojulis*	*albovittata*	SWIO-16	REU1011	IPCOM444-10	JF435176	JF457660	JF458271
Labridae	*Stethojulis*	*albovittata*	SWIO-16	REU1012	IPCOM445-10	JF435175	JF457659	JF458270
Labridae	*Stethojulis*	*albovittata*	SWIO-32	145-1	IPCOM454-10	JF435174	JF457658	JF458269
Labridae	*Stethojulis*	*bandanensis*	MBIO-07	MBIO0792	IPCOM047-10	JF435177	JF457661	JF458272
Labridae	*Stethojulis*	*strigiventer*	MGNW-12	NBE1002	IPCOM262-10	JF435179	JF457663	JF458274
Labridae	*Stethojulis*	*strigiventer*	MGNW-23	NBE-1426	IPCOM324-10	JF435178	JF457662	JF458273
Labridae	*Thalassoma*	*amblycephalum*	MGNW-06	NBE0435	IPCOM208-10	JF435183	JF457667	-
Labridae	*Thalassoma*	*amblycephalum*	MGNW-10	NBE0673	IPCOM261-10	JF435182	JF457666	-
Labridae	*Thalassoma*	*amblycephalum*	MGNW-17	NBE1181	IPCOM294-10	JF435181	JF457665	-
Labridae	*Thalassoma*	*amblycephalum*	MGNW-17	NBE1182	IPCOM295-10	JF435180	JF457664	-
Labridae	*Thalassoma*	*genivittatum*	SWIO-29	075-1	IPCOM391-10	JF435188	JF457672	JF458278
Labridae	*Thalassoma*	*genivittatum*	SWIO-13	REU0761	IPCOM398-10	JF435187	JF457671	JF458277
Labridae	*Thalassoma*	*genivittatum*	SWIO-15	REU0921	IPCOM421-10	JF435189	JF457673	JF458279
Labridae	*Thalassoma*	*genivittatum*	SWIO-23	REU1618	IPCOM466-10	JF435186	JF457670	JF458276
Labridae	*Thalassoma*	*genivittatum*	SWIO-23	REU1619	IPCOM467-10	JF435185	JF457669	JF458275
Labridae	*Thalassoma*	*genivittatum*	SWIO-41	REU2627	IPCOM498-10	JF435184	JF457668	-
Labridae	*Thalassoma*	*hardwicke*	MGNW-10	NBE0646	IPCOM251-10	JF435196	JF457680	-
Labridae	*Thalassoma*	*hardwicke*	MGNW-19	NBE1277	IPCOM308-10	JF435195	JF457679	-
Labridae	*Thalassoma*	*hardwicke*	MGNW-19	NBE1278	IPCOM309-10	JF435194	JF457678	-
Labridae	*Thalassoma*	*hardwicke*	MGNW-19	NBE1279	IPCOM310-10	JF435193	JF457677	-
Labridae	*Thalassoma*	*hardwicke*	SWIO-02	REU0111	IPCOM339-10	JF435192	JF457676	-
Labridae	*Thalassoma*	*hardwicke*	SWIO-41	REU2624	IPCOM496-10	JF435191	JF457675	-
Labridae	*Thalassoma*	*hardwicke*	SWIO-41	REU2625	IPCOM497-10	JF435190	JF457674	-
Labridae	*Thalassoma*	*hebraicum*	MGNW-01	NBE0051	IPCOM119-10	JF435199	JF457683	JF458281
Labridae	*Thalassoma*	*hebraicum*	MGNW-10	NBE0641	IPCOM247-10	JF435198	JF457682	-
Labridae	*Thalassoma*	*hebraicum*	MGNW-10	NBE0642	IPCOM248-10	JF435197	JF457681	JF458280
Labridae	*Thalassoma*	*lunare*	MGNW-01	NBE0033	IPCOM103-10	JF435203	JF457687	-
Labridae	*Thalassoma*	*lunare*	MGNW-04	NBE0239	IPCOM161-10	JF435202	JF457686	-
Labridae	*Thalassoma*	*lunare*	MGNW-17	NBE1233	IPCOM297-10	JF435201	JF457685	-
Labridae	*Thalassoma*	*lunare*	MGNW-17	NBE1234	IPCOM298-10	JF435200	JF457684	-
Labridae	*Thalassoma*	*lutescens*	MBIO-07	MBIO0733	IPCOM043-10	JF435204	JF457688	-
Labridae	*Thalassoma*	*purpureum*	SWIO-21	REU1683	IPCOM471-10	JF435209	JF457693	JF458286
Labridae	*Thalassoma*	*purpureum*	SWIO-30	REU1755	IPCOM479-10	JF435208	JF457692	JF458285
Labridae	*Thalassoma*	*purpureum*	SWIO-30	REU1759	IPCOM480-10	JF435207	JF457691	JF458284
Labridae	*Thalassoma*	*purpureum*	SWIO-30	REU1814	IPCOM482-10	JF435206	JF457690	JF458283
Labridae	*Thalassoma*	*purpureum*	SWIO-30	REU1815	IPCOM483-10	JF435205	JF457689	JF458282
Labridae	*Thalassoma*	*quinquevittatum*	MBIO-04	MBIO0215	IPCOM015-10	JF435210	JF457694	-
Labridae	*Thalassoma*	*trilobatum*	MBIO-04	MBIO0221	IPCOM016-10	JF435212	JF457696	-
Labridae	*Thalassoma*	*trilobatum*	MBIO-07	MBIO0746	IPCOM044-10	JF435211	JF457695	-
Labridae	*Wetmorella*	*nigropinnata**	MBIO-01	MBIO0110	IPCOM013-10	JF435215	JF457699	JF458289
Labridae	*Wetmorella*	*nigropinnata*	MBIO-01	MBIO0199	IPCOM014-10	JF435214	JF457698	JF458288
Labridae	*Wetmorella*	*nigropinnata*	MGNW-01	NBE0116	IPCOM140-10	JF435213	JF457697	JF458287

* Also used as outgroups.
